# Optimization of a High-Throughput 384-Well Plate-Based Screening Platform with *Staphylococcus aureus* ATCC 25923 and *Pseudomonas aeruginosa* ATCC 15442 Biofilms

**DOI:** 10.3390/ijms21093034

**Published:** 2020-04-25

**Authors:** Shella Gilbert-Girard, Kirsi Savijoki, Jari Yli-Kauhaluoma, Adyary Fallarero

**Affiliations:** 1 Drug Research Program, Division of Pharmaceutical Biosciences, Faculty of Pharmacy, University of Helsinki, FI-00014 Helsinki, Finland; kirsi.savijoki@helsinki.fi (K.S.); adyary.fallarero@helsinki.fi (A.F.); 2 Drug Research Program, Division of Pharmaceutical Chemistry and Technology, Faculty of Pharmacy, University of Helsinki, FI-00014 Helsinki, Finland; jari.yli-kauhaluoma@helsinki.fi

**Keywords:** bacteria, biofilms, crystal violet, resazurin, screening, *Staphylococcus aureus*, *Pseudomonas aeruginosa*

## Abstract

In recent years, bacterial infections have become a main concern following the spread of antimicrobial resistance. In addition, bacterial biofilms are known for their high tolerance to antimicrobials and they are regarded as a main cause of recalcitrant infections in humans. Many efforts have been deployed in order to find new antibacterial therapeutic options and the high-throughput screening (HTS) of large libraries of compounds is one of the utilized strategies. However, HTS efforts for anti-biofilm discovery remain uncommon. Here, we miniaturized a 96-well plate (96WP) screening platform, into a 384-well plate (384WP) format, based on a sequential viability and biomass measurements for the assessment of anti-biofilm activity. During the assay optimization process, different parameters were evaluated while using *Staphylococcus aureus* and *Pseudomonas aeruginosa* as the bacterial models. We compared the performance of the optimized 384WP platform to our previously established 96WP-based platform by carrying out a pilot screening of 100 compounds, followed by the screening of a library of 2000 compounds to identify new repurposed anti-biofilm agents. Our results show that the optimized 384WP platform is well-suited for screening purposes, allowing for the rapid screening of a higher number of compounds in a run in a reliable manner.

## 1. Introduction

Biofilms are multicellular communities of bacteria that are attached to a surface and embedded in a self-produced polymeric matrix that provide them with protection in various environments, including in the human body. Biofilms are well known for their greater tolerance, not only to the host immune system, but also to antimicrobials; they can withstand 10- to 1000-fold higher concentrations than cells in planktonic form [1,2,3]. These features make biofilms very difficult to treat and, as a result, they are responsible for the majority of recalcitrant infections, often leading to chronic infections and resulting in a high cost to the healthcare system as well as significant morbidity and mortality [3,4,5,6]. Antibiotic treatments can eliminate planktonic cells, but they fail to eradicate biofilms. This has been attributed to a combination of many mechanisms, including the physical protection given by the biofilm matrix, heterogeneity in the metabolic activity of the cells residing within the biofilm, and specific gene expression changes leading to a biofilm-specific phenotype [3,7,8]. In many cases, the only possible treatment of a persistent biofilm infection is to surgically remove the biofilm. In the case of medical device-related infections, the removal and replacement of the implant is often needed [9]. In addition to a biofilm’s intrinsic tolerance to antimicrobials, the development of antibiotic resistance is also a major problem, as increasing amounts of pathogens are multi-resistant to the currently used antibiotics [10,11]. For all of these reasons, finding new therapeutic options that are effective against biofilms is crucial, and intensive research is now ongoing for discovering new antimicrobials [12].

One approach for tackling this challenge is conducting high-throughput screening (HTS) of compound libraries using assays that test the antimicrobial activity of compounds on biofilms grown in 96-well plates (WP) [13,14]. This approach is very commonly used in early drug discovery, as it allows the rapid testing of a large number of compounds in a single run, using very small amount of the tested compounds [4,15,16,17]. The scientific community has extensively used microplates to grow bacterial biofilm [13,18,19,20,21,22,23], and it has been the preferred sample format used by our group in several studies. For instance, our previous efforts led to the optimization of a phased-screening platform in 96WP, which allowed for monitoring the anti-biofilm activity against *Staphylococcus aureus* by measuring the viability, biomass, and the biofilm matrix [14,15,24]. A primary screening for effects on viable cells and biofilm biomass was conceived, so that two assays could be sequentially performed in a single plate, in order to reduce the time and the amount of materials and compounds required for the analysis. A follow-up assay, involving the fluorescence staining of the poly-β-1,6-*N*-acetyl-D-glucosamine (PNAG) component, was further optimized for measuring the specific effects of active compounds on the biofilms matrix of *S. aureus* [24].

For the initial screening analysis, assays that were based on resazurin and crystal violet staining were first optimized to measure the viability and biofilm biomass of *S. aureus*, respectively. Resazurin is a blue non-fluorescent dye that is only reduced in metabolically active cells into resorufin, a pink fluorescent product that can then be measured. The resazurin staining assay is widely used to evaluate the viability of bacterial cells, as it is fast, shows good repeatability, and has the advantage of discriminating live and dead cells [13]. However, it requires high cell density and living but non-growing cells within biofilms cannot be detected by this method [15]. On the other hand, crystal violet is a widely used basic purple dye that stains negatively charged surfaces (e.g., cell membranes and polysaccharides in the biofilm matrix) [25]. It does not differentiate live from dead cells, but it measures a biofilm’s overall biomass, which makes it a useful complementary assay to resazurin staining [24]. Despite the benefits of these staining methods, screening larger libraries using the 96WP-based platform is still labor-intensive and it requires a significant quantity of the tested compounds.

In view of this, we miniaturized both the resazurin and crystal violet assays for microtiter plates with 384 wells (384WP) to test the applicability of this platform to screen larger number of compounds within a same plate in a shorter time and requiring less reagents per well. The optimization was performed with *S. aureus* ATCC 25,923 and *Pseudomonas aeruginosa* ATCC 15442, two representative model organisms for gram-positive and gram-negative bacteria, respectively. Parameters, such as the working volume, the concentration of the bacterial inoculum (CFU mL^−1^), as well as the resazurin and crystal violet staining conditions, were optimized. Throughout the optimization process, the screening window coefficient known as the Z’ factor (Z’), a statistical parameter monitoring the reliability of the screening assay and assay performance, was measured. The signal-to-noise (S/N) ratio was also monitored. The inhibition of biofilm formation by well-known antibiotics was compared in both 384WP and 96WP to validate the miniaturized model. A proof-of-concept pilot screen of 100 compounds was also performed against *S. aureus* biofilms in both platforms in order to evaluate the correlation of results between 384WP and 96WP. Based upon these results, a screening campaign against *S. aureus* biofilms was performed while using a library of 2000 structurally diverse compounds, including approved drugs, natural products, as well as biologically relevant compounds that have been reported in peer-reviewed publications. The main goal here was to identify repurposed anti-biofilm compounds because this library was composed by approximately 60% of FDA-approved or internationally marketed drugs.

Different methods for quantifying the effect of antibacterial compounds have earlier been miniaturized for 384WP, but such efforts have been particularly uncommon for anti-biofilm screening. For example, an optical density (OD)-based minimum inhibitory concentration (MIC) assay has been used with *S. aureus* and *P. aeruginosa*; however, it does not allow for biofilm quantification [26,27]. Redox-based assays, such as those based on resazurin, have been optimized in 384WP format for various bacterial species, including *Klebsiella pneumoniae*, *Helicobacter pylori*, *Streptococcus pneumoniae*, *Neisseria* species, *P. aeruginosa*, and *S. aureus*, but these analyses have only involved planktonic cells [28,29,30,31,32]. A combination of an image-based method and the redox-based 2,3-bis(2-methoxy-4-nitro-5-sulfophenyl)-2*H*-tetrazolium-5-carboxanilide (XTT) assay to measure the inhibition of *P. aeruginosa* biofilm formation and dispersion has been developed in 384WP [33]. However, this platform is based on more expensive instrumentation, and it requires the use of a mutant GFP (green fluorescent protein)-tagged bacterial strain, which limits the application of this assay for studying different bacterial strains and species.

By contrast, the 384WP anti-biofilm screening platform that has been miniaturized here involves a cost-friendly combination of sequential staining with resazurin and crystal violet, which can be applied to different bacterial strains and species. For cases where assays conditions that are described here are not directly applicable, all of the optimization steps are described in detail and can be used as guidance. Moreover, both of the assays are fast, easy-to-use and amenable to automation. Thus far, the resazurin assay has been optimized in 384WP for anti-biofilm activity assessment on fungi [34]. In one contribution [35], a combined resazurin and crystal violet-based platform has been used for anti-biofilm screening while using *Staphylococcus epidermidis.* However, the optimization process was not reported, and a comparative performance study between the 384WP and the conventional 96WP platform was not carried out, either. Our experiments demonstrate that the 384WP format can be successfully used for fluorescence- and absorbance-based anti-biofilm HTS with large compound libraries. As demonstrated here, 384WP provide reliable as well as reproducible screening results that are comparable to the 96WP-based platform. To the best of our knowledge, this is the first validation of a platform combining both resazurin and crystal violet staining assays in 384WP on *S. aureus* and *P. aeruginosa*. Our optimization efforts were culminated by a screening campaign that involved 2000 compounds, where 77 hits were identified, for a 3.8% success rate.

## 2. Results and Discussion

### 2.1. Optimizing the Working Volume and Initial Bacterial Concentration for 384WP

When optimizing assays that were aimed at screening a large number of compounds, it is important that these assays are simple to execute, cost-friendly, rapid, and that they require low amounts of materials. These are features shared by both the resazurin and crystal violet staining assays. Since multiple replicates of tested compounds are not typically included, the assays must perform robustly and consistently. To that end, the screening assays must be carefully optimized, and their performance needs to be rigorously monitored, also, throughout screening campaigns, using appropriate screening parameters, such as the screening window coefficient - Z’, which was originally published by [36].

The screening method was optimized in terms of the assay time and the amount of material needed, and the quality of the method was validated by calculating the Z’ factor (Z’). We first optimized the bacterial concentration and working volume for the 384WP-based platform. For these initial experiments, the resazurin and crystal violet assays were performed while using the conditions that were previously described by our group [24]. Five starting concentrations of planktonic bacteria, including the cell concentration routinely used in 96WP for *S. aureus*, were tested. The first of the concentrations was the same as the one used previously in 96WP and the second concentration was proportionally reduced from the first concentration to match the smaller growth area at the bottom of the 384WP and reach a similar initial concentration per surface (CFU cm^−2^) concentration. The tested concentrations for *S. aureus* were 4.00 × 10^6^ CFU ml^−1^, 7.00 × 10^5^ CFU mL^−1^, 2.50 × 10^6^ CFU mL^−1^, 7.00 × 10^4^ CFU mL^−1^, and 7.00 × 10^3^ CFU mL^−1^. In the case of *P. aeruginosa*, the following five cell concentrations were tested: 1.00 × 10^7^ CFU mL^−1^, 6.25 × 10^6^ CFU mL^−1^, 1.75 × 10^6^ CFU mL^−1^, 1.75 × 10^5^ CFU mL^−1^, and 1.75 × 10^4^ CFU mL^−1^.

Each bacterial concentration was tested in 384WP while using two different working volumes (that is, total volume in the wells, 40 µL and 50 µL) and two different growth periods (18 h that is routinely used in pre-exposure experiments in 96WP and 42 h that is used in post-exposure experiments in 96WP). For evaluating the assay performance, the Z’ factor was calculated and then compared between each condition. The Z’ evaluates the separation between the maximal signal and the background signal of an assay and it is the most appropriate and widely used statistical parameter to assess the performance of a screening method [36,37]. It ranges between 0 and 1. A value of 0 signifies no distinction between the maximal and minimal signal, whereas a value of 1 indicates that the signal separation is ideal and infinite (an ideal screening assay). Consequently, a Z’ value that is close to 0 indicates that the assay does not perform well and it requires more optimization. Generally, a Z’ value above 0.5 indicates a well-performing assay, although, in the case of cell-based assays, a Z’ above 0.35 is already considered to be acceptable [38]. The difference between the maximal and minimal signals obtained in each condition was also compared. Parameters that resulted in the best Z’ factor were selected as being optimal. The correlation between the Z’ factor and the signal-to-noise ratio (S/N), as defined by [36], obtained for both assays, during the optimization stage, was also monitored (Supplementary Figure S1a). The two statistical parameters showed a consistent correlation, where a good Z’ value (above 0.5) generally equaled an S/N above six.

**Figure 1:**
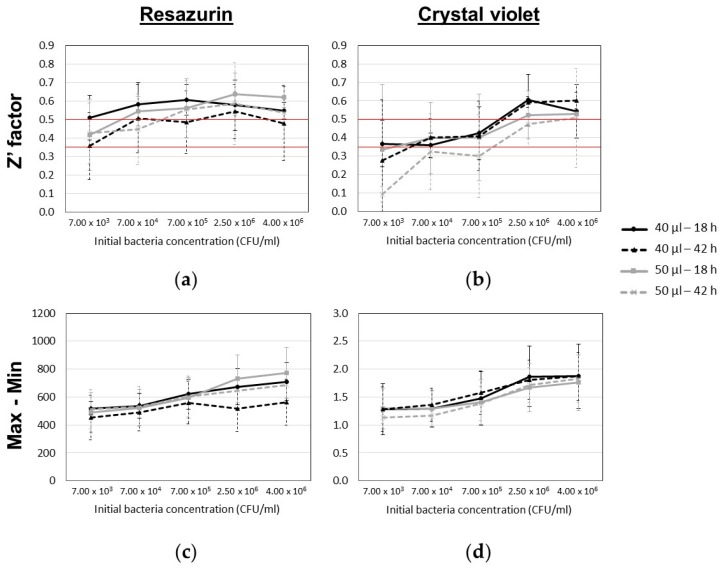
Effect of different initial *S. aureus* concentrations and working volumes on the performance of a miniaturized biofilm screening assay in 384WP. Assay performance is measured using the Z’ factor (**a**,**b**) and the maximum minus minimum signal (**c**,**d**) of the resazurin and crystal violet staining of *S. aureus* biofilms grown for 18 h or 42 h in a volume of 40 µL or 50 µL with different initial bacterial concentration in 384WP. Maximum minus minimum results are expressed in relative fluorescence units (RFUs) for resazurin or relative absorbance units (RAUs) for crystal violet. The results are based on 2–4 independent biological replica experiments, each plate containing 28–40 technical replicates for each condition and 30 media control replicates. The red lines in panels a and b represent the thresholds for determination of the quality of the assay: acceptable (Z = 0.35) and good (Z = 0.5).

When testing the platform with *S. aureus* using both the resazurin and crystal violet on 18 h-old or 42 h-old biofilms (Figure 1), the two different working volumes (40 and 50 µL) did not significantly affect the Z’ values (*p* ≥ 0.05) or the maximum minus the minimum signals. With a working volume as low as 40 µL, it was possible to obtain high quality data and this working volume was therefore selected for further experiments. A lower working volume (25 µL) was also tested, but the wells fully dried out during incubation, so we concluded that so small working volumes in 384WP assays involving longer incubation times would not be feasible due to evaporation issues. The initial *S. aureus* concentration did not significantly impact the Z’ values regarding the resazurin assay; the majority of the tested *S. aureus* concentrations reached a biofilm state with similar metabolic activity after 18 or 42 h of incubation. The 7.00 × 10^4^ CFU mL^−1^ was the lowest cell concentration, resulting in a good Z’ value (≥ 0.5) for both the young (18 h) and more mature (42 h) biofilms. A slightly increased difference between the maximal and minimal signals was observed with increased *S. aureus* concentrations, even though it did not translate into a higher Z’ value.

The crystal violet assay was more influenced by the initial *S. aureus* concentrations, and a lower cell concentration was usually related to a lower Z’ value and a smaller difference between the maximum and minimum signals. This staining method generally resulted in a lower performance than resazurin; it requires more steps that can introduce variability and is more laborious, which translates into a lower reproducibility and a poorer dynamic range [39]. For this assay, all of the cell concentrations from 2.50 × 10^6^ CFU mL^−1^ and higher still resulted in a good Z’ value (≥ 0.5). As our findings indicated that the resazurin staining produces more reliable results than the crystal violet staining, and since both assays are performed sequentially in the same plate, the initial bacterial concentration that was selected for the subsequent assays was the compromise of both staining methods (7.00 × 10^4^ CFU mL^−1^), which performed well with the resazurin staining while still giving an acceptable performance with crystal violet.

**Figure 2:**
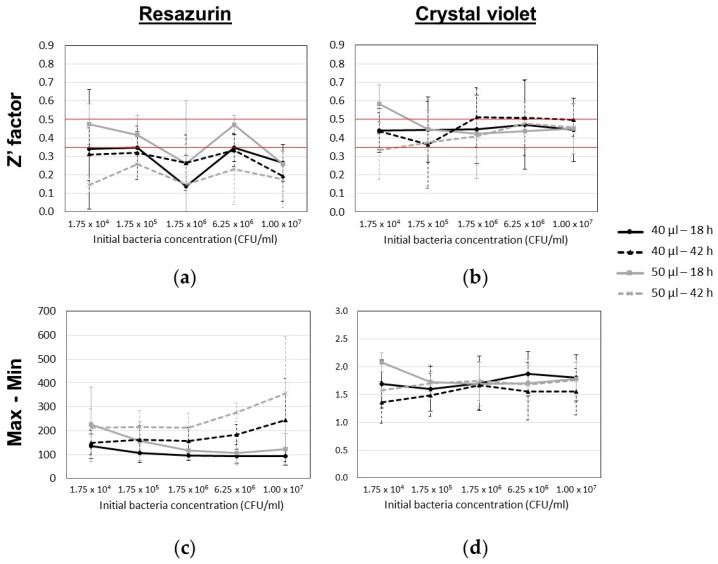
The effect of different initial *P. aeruginosa* concentrations and working volumes on the performance of a miniaturized biofilm screening assay in 384WP. Assay performance is measured using the Z’ factor (**a**,**b**) and the maximum minus minimum signal (**c**,**d**) of the resazurin and crystal violet staining of *P. aeruginosa* biofilms grown for 18 h or 42 h in a volume of 40 µL or 50 µL with different initial bacterial concentration in 384WP. Maximum minus minimum results are expressed in RFUs for resazurin or RAUs for crystal violet. Results are based on 3–4 independent biological replica experiments, each plate containing 28–40 technical replicates for each condition and 30 media control replicates. The red lines in panels a and b represent the thresholds for the determination of the quality of the assay: acceptable (Z = 0.35) and good (Z = 0.5).

The optimized screening platform was also tested with gram-negative *P. aeruginosa* biofilms (Figure 2). With this strain, neither the volume nor the initial bacterial concentration affected the Z’ values for both staining assays (*p* ≥ 0.05). The resazurin assay signals varied significantly, but the position of the biofilm controls (maximal signal) within the plate affected more strongly the metabolic activity of the biofilms than the initial quantity of bacteria. Wells on the edges of the plate showed more varying results, which was also found between different replica plates. While this effect is well known in 96WP, it was particularly noticeable in 384WP, probably because of the smaller size of the wells and smaller working volumes. The resazurin assay did not perform as well with *P. aeruginosa* as it did with *S. aureus* and it rarely resulted in Z’ values above 0.35, indicating the need for optimizing other assay parameters or more accurate optimization of the tested parameters for this gram-negative model. As the conditions for this assay were not fully optimized, these observations were considered to be normal at this point. The crystal violet staining results did not vary much with respect to the working volume or the initial cell concentration. For further experiments, a bacterial concentration of 1.75 × 10^5^ CFU mL^−1^ was selected, as it resulted in lower intra-plate variability when using resazurin as the staining method. For the next part of the optimization, different parameters of the resazurin and crystal violet staining methods were adjusted in order to select the best conditions for both bacterial species.

### 2.2. Optimizing the Conditions for Resazurin Staining

Our pre-existing screening platform developed in-house [24] is based on staining the biofilms first with resazurin and then by crystal violet in the same 96WP. Using both assays has the advantage of giving more information of the activity of an antibacterial compound and its mechanism of action (MoA) and doing it in a single plate allows for correlating the results of each assay to a single biofilm. When evaluating the anti-biofilm potential of a compound, it is important to make a distinction between the compounds that eliminate living cells (i.e., prevent resazurin reduction), and those that prevent biofilm formation/disrupt the biofilm (i.e., reduce crystal violet staining). Analyzing the effects of the compounds on biofilms using both staining methods is of particular importance, because, if only living cells are eliminated, the remaining biofilm matrix becomes an ideal environment for a quick recolonization, leading to a recurrence of the infection [18,40]. The non-invasive nature of resazurin [24,41] allows for the subsequent performance of additional staining assays within the same plate, which minimizes the plate-to-plate variations when using multiple staining methods during the screening process. The correlation between the Z’ factor and the S/N obtained for the optimization of the conditions of both the resazurin and crystal violet assays was monitored (Supplementary Material Figure S1b). Similar to the optimization of working volume and bacteria concentration, there was a clear correlation between the Z’ and the S/N and a good Z’ value (above 0.5) translated into an S/N above 6.

The resazurin assay measures the metabolic activity of the cells, which can greatly vary between different strains, indicating that the method has to be optimized for each tested species or strain [24,42]. Here, the resazurin staining conditions were optimized using 40 µL as the working volume with an initial cell concentration of 7.00 × 10^4^ CFU mL^−1^ for *S. aureus* and 1.75 × 10^5^ CFU mL^−1^ for *P. aeruginosa*. Different resazurin concentrations within the range of 20 µM to 200 µM were tested and fluorescence was measured at different time points (from 5 to 80 min.).

**Figure 3:**
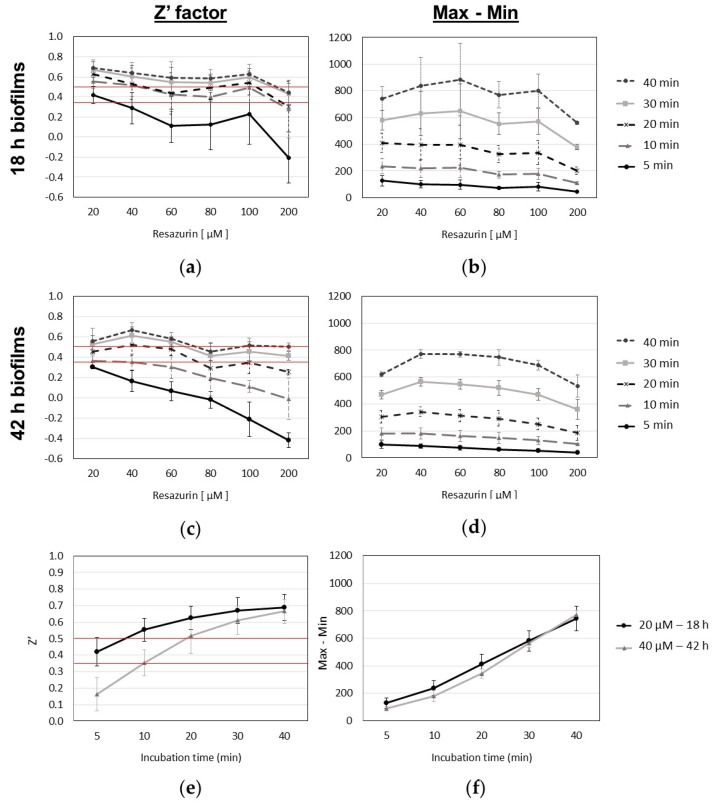
Effect of different resazurin concentrations and incubation times with the dye on the performance of a miniaturized biofilm screening assay in 384WP with *S. aureus*. Assay performance is measured using Z’ factor (**a**,**c**) and maximum minus minimum signal (**b**,**d**) of the resazurin staining of *S. aureus* biofilms grown for 18 h or 42 h in a volume of 40 µL with a concentration of the bacterial inoculum of 7.00 × 10^4^ (CFU) mL^−1^. (**e**,**f**) show the Z’ factor and the maximum minus minimum signal obtained with the best resazurin concentration over time. Maximum minus minimum results are expressed in RFUs. Results are based on 2–4 independent biological replica experiments performed at room temperature (RT), each plate containing from 12 to 42 technical replicates and 16 media control replicates for each condition. The red lines in panels a, c and e represent the thresholds for determination of the quality of the assay: acceptable (Z’ = 0.35) and good (Z’ = 0.5).

With the *S. aureus* 18 h-old biofilms, the Z’ values that were obtained while testing the various concentrations of resazurin (20–100 µM) were consistently above 0.35 after 10 min. of incubation and above 0.5 after 30 min. (Figure 3a). With resazurin at 20 µM, a concentration routinely used in 96WP, the assay performance was overall better when compared to higher resazurin concentrations. In addition, only a minimum of 10 min. of incubation was needed to reach a Z’ above 0.5. With 42 h-old biofilms, resazurin at 40 µM was slightly more optimal and an incubation of at least 30 min. was required to reach a Z’ above 0.5 (Figure 3b), but only a marginal benefit was obtained with a longer incubation time. In general, the longer the incubation was, the better the Z’ factor and the wider the separation between the maximum and minimum signals (Figure 3c,d). On the other hand, while the difference between the maximum and minimum signals significantly increased over time, the increase of the Z’ factor slowed down quickly. Therefore, for further experiments, resazurin at the concentration of 20 µM was selected to test 18 h-old *S. aureus* biofilms and 40 µM for 42 h-old biofilms using 30 min. incubation for each.

**Figure 4:**
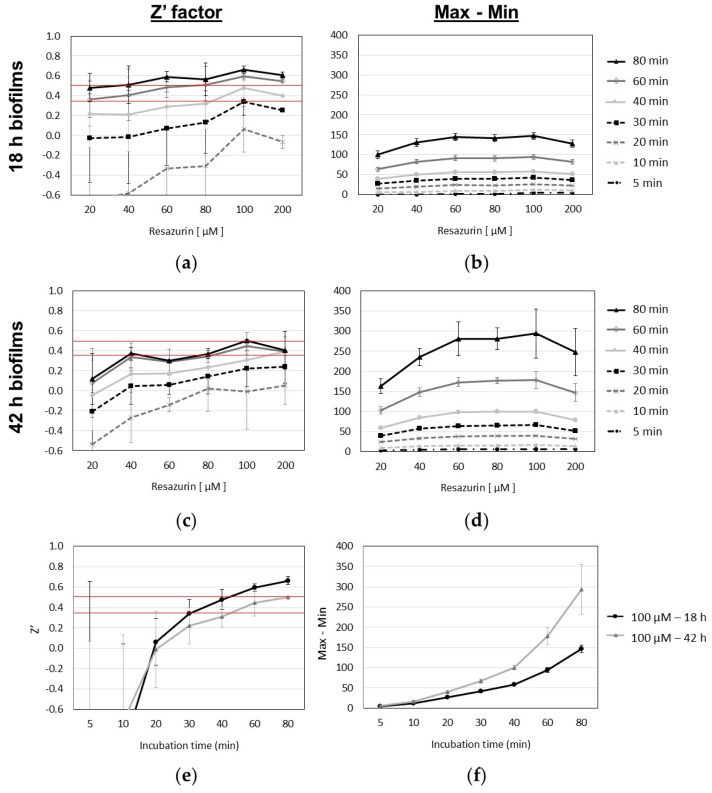
Effect of different resazurin concentrations and incubation times with the dye on the performance of a miniaturized biofilm screening assay in 384WP with *P. aeruginosa*. Assay performance is measured using Z’ factor (**a**,**c**) and maximum minus minimum signal (**b**,**d**) of the resazurin staining of *P. aeruginosa* biofilms grown for 18 h or 42 h in a volume of 40 µL with a concentration of the bacterial inoculum of 1.75 × 10^5^ CFU mL^−1^. (**e**,**f**) show the Z’ factor and the maximum minus minimum signal obtained with the best resazurin concentration over time. Maximum minus minimum results are expressed in RFUs. Results are based on 2 independent biological replica experiments performed at 37 °C, each plate containing from 36 to 48 technical replicates and 12 media control replicates for each condition. The red lines in panels a, c, and e represent the thresholds for determination of the quality of the assay: acceptable (Z’ = 0.35) and good (Z’ = 5).

As expected, different conditions were required for the staining of *P. aeruginosa*, thus confirming the need for species-dependent optimization (Figure 4). With both 18 h-old or 42 h-old biofilms, regardless of the concentration of resazurin used, a Z’ above 0.5 could never be reached and only the longest incubation times would allow for reaching a Z’ of at least 0.3. Despite the higher initial cell concentration used, *P. aeruginosa* produced a much lower fluorescence signal than *S. aureus*. The possible explanations include strain-specific differences affecting the reduction rate of resazurin or a greater thickness or sliminess of *P. aeruginosa* biofilms hampering the penetration of resazurin [42]. Therefore, *P. aeruginosa* biofilms were stained at 37 °C, which significantly increased the Z’ factor, in order to increase the metabolic activity and improve the sensitivity of the resazurin assay. With long incubation times (60–80 min.), all of the tested resazurin concentrations provided acceptable (Z’ > 0.35) to good (Z’ > 0.5) performance values with 18 h-old *P. aeruginosa* biofilms (Figure 4a). The resazurin concentration of 100 µM resulted in the best Z’ factor with both the 18 h and 42 h-old biofilms (Figure 4a,c). An incubation of at least 60 min. with 18 h-old biofilms and 80 min. with 42 h-old biofilms was necessary to ensure proper assay performance. Of note, performing the assay at 37 °C also increases the fluorescence signal obtained from the *S. aureus* biofilms, but further resazurin staining assays with *S. aureus* were still performed at RT, as carrying out the assay at RT was enough to obtain good assay performance.

### 2.3. Optimizing the Conditions for Crystal Violet Staining

The crystal violet staining was performed on the same biofilms that were used for the resazurin staining. Different concentrations of crystal violet and lengths of incubation times were tested with both bacterial species. In the case of *S. aureus*, the absorbance of the stained biomass was also measured at different time points after the addition of ethanol to dissolve the bound crystal violet. The Z’ factor increased proportionally to the dissolution time for one hour and then stabilized (Supplementary Figure S2b). An hour was consequently selected as the most optimal dissolution time for the bound crystal violet. Additionally, different incubation times with the dye (5, 10, and 15 min.) did not significantly affect the assay performance (Supplementary Figure S2a); therefore, the shortest time (5 min.) was selected for further assays. While the crystal violet assay more variable results than resazurin [42], statistically acceptable results could still be obtained in our study.

**Figure 5:**
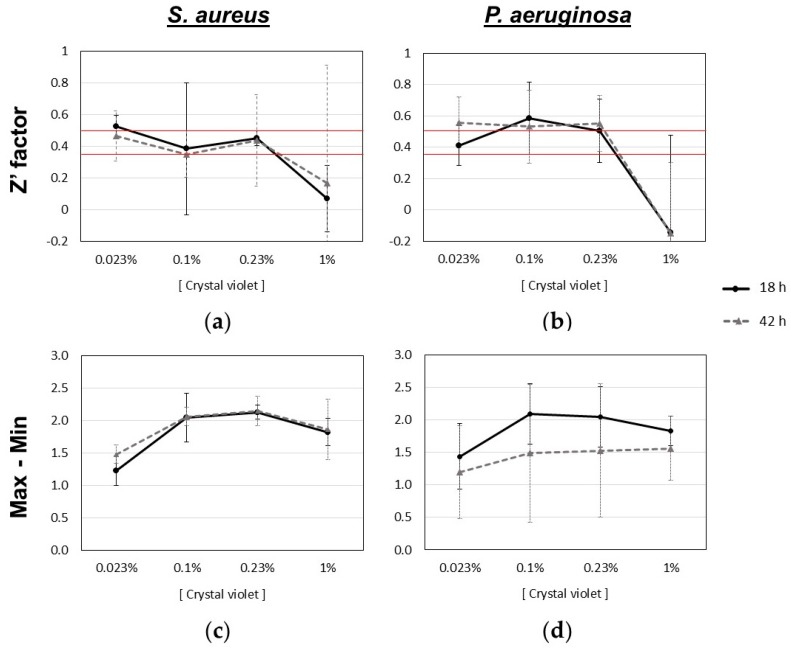
Effect of different crystal violet concentrations on the performance of a miniaturized biofilm screening assay in 384WP. Assay performance is measured using Z’ factor (**a**,**b**) and maximum minus minimum signal (**c**,**d**) of the crystal violet staining of *S. aureus* and *P. aeruginosa* biofilms grown for 18 h or 42 h in a volume of 40 µL with a concentration of the bacterial inoculum of 7.00 × 10^4^ CFU mL^−1^ (*S. aureus*) or 1.75 × 10^5^ CFU mL^−1^ (*P. aeruginosa*). Maximum minus minimum results are expressed in RAUs. Results are based on 3–5 independent biological replica experiments, with each plate containing from eight (one experiment) to 96 technical replicates and 12 to 32 media control replicates for each condition. The red lines in panels a and b represent the thresholds for determination of the quality of the assay: acceptable (Z = 0.35) and good (Z = 5).

With *S. aureus* biofilms (Figure 5a,c) the crystal violet at 0.023% (*v/v*) provided higher Z’ values, although the difference between the maximum and minimum signal was greater with higher crystal violet concentrations. The most likely reason for this could be the reduced inter-well variability detected with lower crystal violet concentrations, since using higher concentrations of crystal violet occasionally led to darkly stained media control wells containing no bacteria. With *P. aeruginosa* (Figure 5b,d), the three lowest concentrations of crystal violet resulted in very similar Z’ factors and maximum minus minimum values, especially on 42 h-old biofilms. The crystal violet concentration of 0.1% (*v/v*) gave an average Z’ value above 0.5 with both young and older *P. aeruginosa* biofilms.

### 2.4. Biofilm Formation in 384WP in Comparison with 96WP

**Figure 6:**
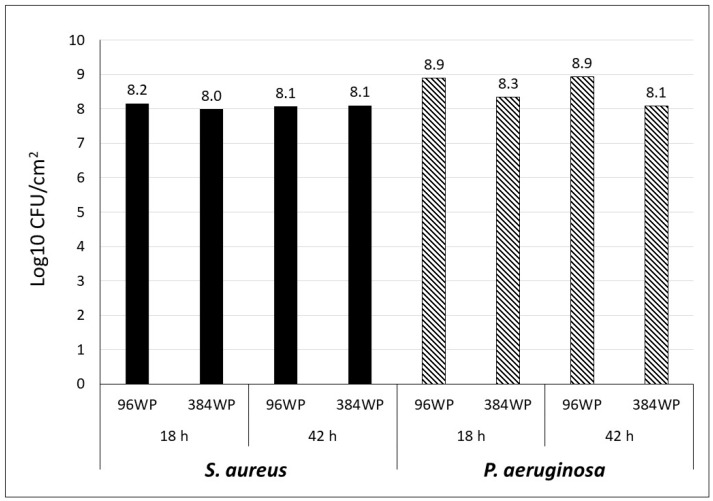
Log10 of the CFUs per cm^2^ of *S. aureus* (black) and *P. aeruginosa* (striped) biofilms grown in 96WP or 384WP for 18 h or 42 h. The data represents the average of 16–22 biofilms (technical replicates) from 2–5 independent biological replica experiments.

Next, we compared the number of viable cells attached as biofilms in 384WP versus 96WP. The *S. aureus* and *P. aeruginosa* biofilms that were produced in each plate were scraped, serially diluted, and plated on tryptic soy agar (TSA). Viable colonies (CFUs) were counted the next day and the amount of CFUs in each biofilm was normalized to the area (cm^2^) of the wells in both systems (Figure 6). With *S. aureus*, a similar amount of CFU cm^−2^ was observed between the 18 h-old and 42 h-old biofilms, as well as in the 96WP and 384WP plates, suggesting a similar cell density. On the other hand, with *P. aeruginosa*, the number of CFU cm^−2^ was slightly reduced by 0.6–0.8 log in 384WP when compared to 96WP. It could be that biofilms that formed by *P. aeruginosa* were less dense or had a different structure in the smaller wells of 384WP, which stimulated cell dispersal. However, overall, a relatively similar cell density was still achieved in biofilms that were grown for 18 h and 42 h in each plate model. It is possible that, in the restricted space of the wells, biofilms reached a maximal number of cells within the given area or that the time span between the two time points studied was not long enough to result in a detectable cell density difference. In any case, the differences between responses in 18 h versus 42 h biofilms cannot be attributed to a larger number of viable cells attached on the wells, at least on our assay conditions.

### 2.5. Susceptibility of S. aureus Biofilms Formed in 384WP and 96WP to Antibiotics

**Figure 7:**
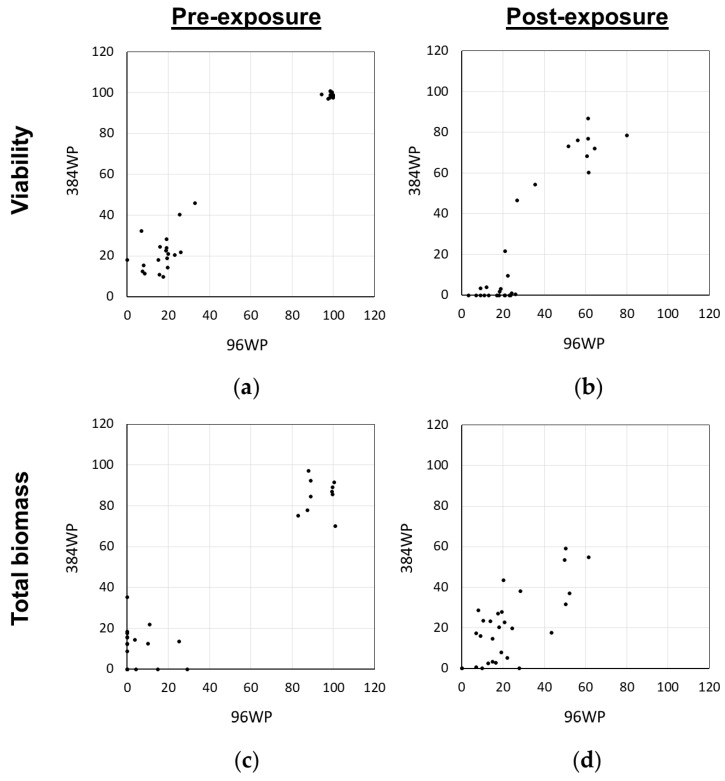
Correlation of the susceptibility of *S. aureus* biofilms to different concentrations of four antibiotics (rifampicin, oxacillin, doxycycline, penicillin G) in 96WP and 384WP in pre- (**a**,**c**) and post-exposure (**b**,**d**) modes. The effect of the antibiotics on the biofilms was assessed by resazurin (“Viability”, (**a**,**b**)) and crystal violet (“Total Biomass”, (**c**,**d**)) staining. Results are expressed as inhibition percentages of viability or biomass in comparison to untreated biofilms and they are based on two independent biological replica experiments, with two (96WP) or eight (384WP) technical replicates for each condition.

We further evaluated the susceptibility of *S. aureus* biofilms to a panel of standard antibiotics at seven different concentrations (10 µM to 0.01 pM) while using the pre- and post-exposure modes in order to compare the screening results obtained between the miniaturized 384WP platform and the characterized 96WP platform (Figure 7). Overall, the susceptibility of the biofilms was comparable in both models, which confirms the applicability of the 384WP model for antibacterial activity assessment. The biofilm viability inhibition percentages that were obtained with the resazurin assay in the pre-exposure mode were very similar between the 96WP and the 384WP, with an average difference of inhibition percentage under 6 % (5.5% ± 5.8%). When using the post-exposure mode, the difference between the viability inhibition results that were obtained in 96WP and in 384WP increased, with an average difference of inhibition of almost 15% (14.3% ± 7.7%). With the crystal violet assay, an average difference of biomass formation inhibition of 11.4% ± 8.9% was observed between 96WP and 384WP in pre-exposure and 10.6% ± 7.5% in post-exposure. Thus, performing the assays in 384WP rather than in 96WP produced a very similar outcome and did not result in generally higher or lower inhibition values at the different concentrations of antibiotics tested. Still, it is important to note that, in our experiments, with both species, the initial bacterial concentration was about 70 times lower in 384WP than in 96WP, which could have affected the antimicrobial susceptibility during the early stages of bacterial growth prior to biofilm formation and especially in pre-exposure experiments. Some compounds that have a rapid MoA on bacteria might perform differently in both models when using the same assay conditions that are described here.

### 2.6. Antibacterial Pilot Screening Against S. aureus Biofilms in 384WP and 96WP Platforms

**Figure 8:**
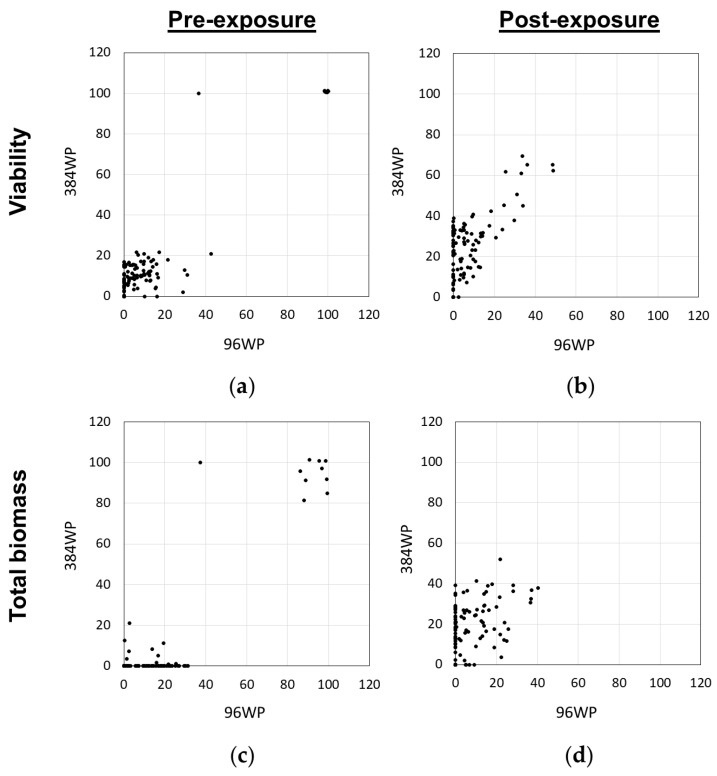
Inhibition of *S. aureus* biofilms by 100 compounds tested at 10 µM in 96WP and 384WP in pre (**a**,**c**) and post-exposure (**b**,**d**) modes. The effect of the compounds on the biofilms was assessed by resazurin (“Viability, (**a**,**b**)) and crystal violet (“Total biomass”, (**c**,**d**)) staining. Results are expressed as inhibition percentages of viability or biomass in comparison to untreated biofilms and they are based on two independent biological replica experiments, with one (96WP) or two (384WP) technical replicates for each condition.

We performed a pilot screen of 100 compounds, which included natural derivatives as well as 10 antibiotics, at 10 µM, to further verify the applicability of 384WP for antibacterial screening in a larger scale (Figure 8). The list of all of the tested compounds, sources, and their screening results in 96WP and 384WP is available in the Supplementary Materials (Table S1). Similar to previous findings, the majority of the results correlated between 384WP and 96WP, but a slight difference was observed; the inhibition values that were obtained using post-exposure mode in 384WP were generally higher than values obtained in 96WP (by 18% with resazurin and 11% with crystal violet). This difference was not observed with the pre-exposure mode and it would be unlikely to cause major differences in the conclusions of a screening, since screenings in the early discovery of antibacterial compounds typically evaluate the prevention of growth (pre-exposure) rather than the antimicrobial effects on pre-existing biofilms (post-exposure). It is still a difference that must be taken in consideration for post-exposure screenings.

In the pre-exposure mode, one of the antibiotics (streptomycin) resulted in significantly different inhibition values in 384WP and 96WP. An inhibition of 100% with streptomycin was observed in 384WP, while this antibiotic only inhibited 30 to 40% of the biofilm formation in 96WP. Such a phenomenon was exclusively seen with streptomycin. It is plausible that the lower concentration of the bacterial cells in 384WP in the beginning of the experiment affected the outcome of the assay. However, this difference between 384WP and 96WP was not observed when using the post-exposure mode for this antibiotic. This can be explained by the fact that, after 18 h (when the treatment with streptomycin starts in the post-exposure assay), the number of viable cells attached is very similar between 384WP and 96WP (Figure 6), making comparable effects of streptomycin in both systems. Overall, the average difference in the resazurin and crystal violet staining results between 384WP and 96WP for the same compounds was 8.7% ± 9.3% in pre-exposure and 15.6% ± 10.5% in post-exposure. Therefore, the results that were obtained in both plates were quite similar for the indicated compounds. Of note, the variations between replicates were slightly higher in 384WP, with an average SD that was approximately 4% higher in 384WP than in 96WP. Taken together, we conclude that the optimized platform performed in 384WP is as reliable as the 96WP-based model and it can be used to screen novel anti-biofilms.

### 2.7. Screening Campaign Against S. aureus Biofilms in 384WP

**Figure 9:**
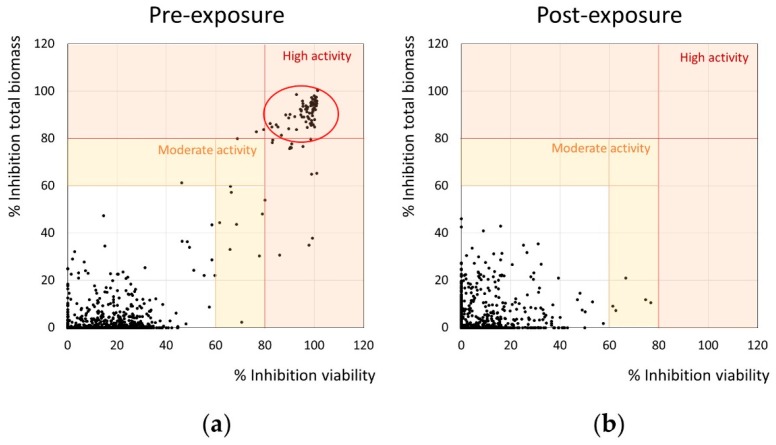
Results of the screening of a compound library of 2000 compounds tested at 10 µM against *S. aureus* biofilms grown in 384WP. (**a**): prevention of biofilm formation (pre-exposure). (**b**): destruction of 18h-old pre-formed biofilms (post-exposure). The effect of the compounds on the biofilms was assessed by resazurin (x axis) and crystal violet (y axis) staining. The red lines represent the inhibition thresholds used to select active compounds. Results are expressed as inhibition percentages of viability or biomass in comparison to untreated biofilms and they are based on two independent biological replica experiments.

The optimized 384WP platform was next used to screen a library containing 2000 compounds against *S. aureus* biofilms, both for the capacity to prevent biofilm formation (pre-exposure mode) and to destroy pre-formed biofilms (post-exposure mode) (Figure 9). As in the pilot screen, the compounds were tested at 10 µM and biofilms were grown and analyzed, as described above. We set-up empiric thresholds to categorize the screening results. Compounds displaying an inhibition of at least 80% were regarded as highly active, while compounds with an inhibitory activity of at least 60% were considered to be moderately active. The identity of the compounds and the screening results are available as supplementary material (Table S2). Overall, 77 compounds were able to prevent biofilm formation by at least 80% (according to both the resazurin and crystal violet staining). A list of all these hits and their inhibitory activity is provided (Table A1, Appendix A). However, no compound significantly affected pre-formed biofilms, which is not surprising when considering the high chemotolerance of biofilms. Of the 77 positive hits initially identified in pre-exposure, 50 were known antibacterial compounds and seven were known to be anti-infective, which suggests that the screening platform worked well in identifying compounds with previously recognized antibacterial activity. Five other hits were known antifungal compounds: econazole nitrate, amphotericin B, sulconazole nitrate, miconazole nitrate, and phenylmercuric acetate. For some of those, antibacterial activity has already been reported [43,44], but no prior studies have been done on the antibacterial activity of phenylmercuric acetate or sulconazole, at least to be best of our knowledge. It is interesting to note that amphotericin B is generally thought to be inactive on bacteria, arguably either because of the drug’s sterol-based mechanism of action or because of the target’s membrane structure [45]. Seven additional hits were neoplastic compounds: teniposide, dactinomycin, mitomycin C, floxuridine, celastrol, agelasine, and carmofur. The antibacterial activity of those compounds has been investigated before and some of them seem to hold potential for treatment of bacterial infections, but their high cytotoxicity generally prevents their use as antibiotics [46,47,48,49,50,51]. Two of the remaining initial hits are anti-helminthic drugs. The first, pyrvinium pamoate, has also been identified in a screening against methicillin-resistant *Staphylococcus aureus* (MRSA) [52]. The second, niclosamide, has been repeatedly identified in screening campaigns and investigated for its many potential new purposes, including as an antibacterial drug [51,53,54,55]. The five remaining hits, triamcinolone acetonide, chlorthalidone, physostigmine salicylate, chlorgiline hydrochloride, and chlorpheniramine maleate, were known for diverse functions that were unrelated to antimicrobial treatment. However, after performing new follow-up studies on re-purchased compounds, their antibacterial activity could not be confirmed, and these hits were not pursued any further.

## 3. Materials and Methods

### 3.1. Bacterial Growth and Biofilm Formation

The biofilm-producing strains of *S. aureus* ATCC 25,923 (Faculty of Pharmacy, University of Helsinki, Helsinki, Finland) and *P. aeruginosa* ATCC 15,442 (provided by the Laboratory of Microbiology, Parasitology and Hygiene (LMPH), University of Antwerp, Antwerpen, Belgium) were seeded on tryptic soy agar (TSA) (LAB011, Lab M Ltd., Heywood, United Kingdom) and grown overnight under aerobic conditions at 37 °C. The bacteria were then suspended in 5 mL of tryptic soy broth (TSB) (LAB004, Lab M Ltd.) and grown to exponential phase under aerobic conditions at 37 °C with shaking (220 rpm) until the cells reached approximately 4.00 × 10^8^ CFU (Colony Forming Unit) mL^−1^ for *S. aureus* and 1.00 × 10^9^ CFU mL^−1^ for *P. aeruginosa*. The biofilms were formed in polystyrene 384WP (Nunc^TM^ 242757, Thermo Fisher Scientific, Roskilde, Denmark) or 96WP (Nunc^TM^ 161093, Thermo Fisher Scientific, Roskilde, Denmark) from various dilutions (for *S. aureus*: 4.00 × 10^6^ CFU mL^−1^, 2.50 × 10^6^ CFU mL^−1^, 7.00 × 10^5^ CFU mL^−1^, 7.00 × 10^4^ CFU mL^−1^, and 7.00 × 10^3^ CFU mL^−1^; for *P. aeruginosa*: 1.00 × 10^7^ CFU mL^−1^, 6.25 × 10^6^ CFU mL^−1^, 1.75 ×10^6^ CFU mL^−1^, 1.75 ×10^5^ CFU mL^−1^, and 1.75 × 10^4^ CFU mL^−1^) of the initial bacterial suspension. For each species, the plates were incubated for 18 h or 42 h under aerobic conditions at 37 °C, 220 rpm. For the 42 h-old biofilms, the media was changed after 18 h of incubation.

### 3.2. Resazurin Staining

The resazurin staining was performed, as described earlier [15], with some modifications. Briefly, the planktonic solution was discarded very gently, attempting to minimize any disruptions to the biofilms. The biofilms were washed once with a working volume (either 40 µL or 50 µL in 384WP or 200 µL in 96WP) of phosphate buffered saline (PBS) (BR0014, Thermo Fisher Scientific, Basingstoke, England). The washed biofilm cells were incubated with different concentrations of resazurin (20, 40, 60, 80, 100, and 200 µM) in the darkness at room temperature (RT) or at 37 °C (when indicated), with agitation (220 rpm) for various periods of time (*S. aureus*: 5, 10, 20, 30, and 40 min; *P. aeruginosa*: 5, 10, 20, 30, 40, 60, and 80 min.). The resazurin sodium salt (R7017, Sigma–Aldrich, St. Louis, MO, USA) was dissolved in PBS to obtain a stock solution of 400 µM (or 0.1 mg mL^−1^), which was subsequently diluted in PBS to achieve the indicated concentrations for staining the biofilm cells. Fluorescence was measured at λ_excitation_ = 560 nm and λ_emission_ = 590 nm while using the top optics of the Varioskan LUX Multimode microplate reader (Thermo Scientific, Vantaa, Finland). After the measurement, the resazurin solution was removed from the wells, and the biofilms were stained with crystal violet, as follows.

### 3.3. Crystal Violet Staining

After resazurin staining, biofilms were subsequently stained with crystal violet as previously reported [14]. To fix the biofilms, a working volume of ethanol 100% (either 40 µL or 50 µL in 384WP or 200 µL in 96WP) was added onto the biofilms and the 96WP/384WP plates were incubated at RT. After 15 min. the lids were removed, and wells were allowed to air-dry completely. The biofilms were stained with various concentrations (0.023%, 0.1%, 0.23%, 1% *v/v*) of crystal violet (HT90132, Sigma-Aldrich, St. Louis, MO, USA) at RT for five minutes and then washed twice with deionized H_2_O and left to dry a few minutes (5 min. for 96WP, up to 10 min. for 384WP). The crystal violet bound to the biofilms was solubilized in 96% ethanol for 1 h at RT. The absorbance was measured at 595 nm with a Multiskan Sky microplate spectrophotometer (Thermo Scientific, Vantaa, Finland).

### 3.4. Comparing the CFUs of Biofilms formed in 384WP and 96WP

The biofilms of *S. aureus* and *P. aeruginosa* were grown in both 96WP and 384WP, as described previously. The bacteria were seeded in the wells at a concentration of 4.00 × 10^6^ CFU mL^−1^ (*S. aureus*) or 1.00 × 10^7^ CFU mL^−1^ (*P. aeruginosa*) in 96WP and 7.00 × 10^4^ CFU mL^−1^ (*S. aureus*) or 1.75 × 10^5^ CFU mL^−1^ (*P. aeruginosa*) in 384WP. The concentration of the bacterial cells selected for 384WP was based on the obtained results during the optimization steps. After 18 h or 42 h, the planktonic bacteria were discarded, and biofilms were washed once with a working volume of PBS. The biofilms were then scraped using a pipette tip in either 20 µL (384WP) or 100 µL (96WP) of PBS and clumps were thoroughly broken by repeated pipetting. The bacteria were serially diluted in PBS and 10 µL drops of the dilutions (10^−4^ to 10^−7^) were spotted on TSA to measure the formation of viable colonies.

### 3.5. Comparing the Antibiotic Susceptibility of S. aureus Biofilms in 384WP and 96WP

**Scheme 1:**
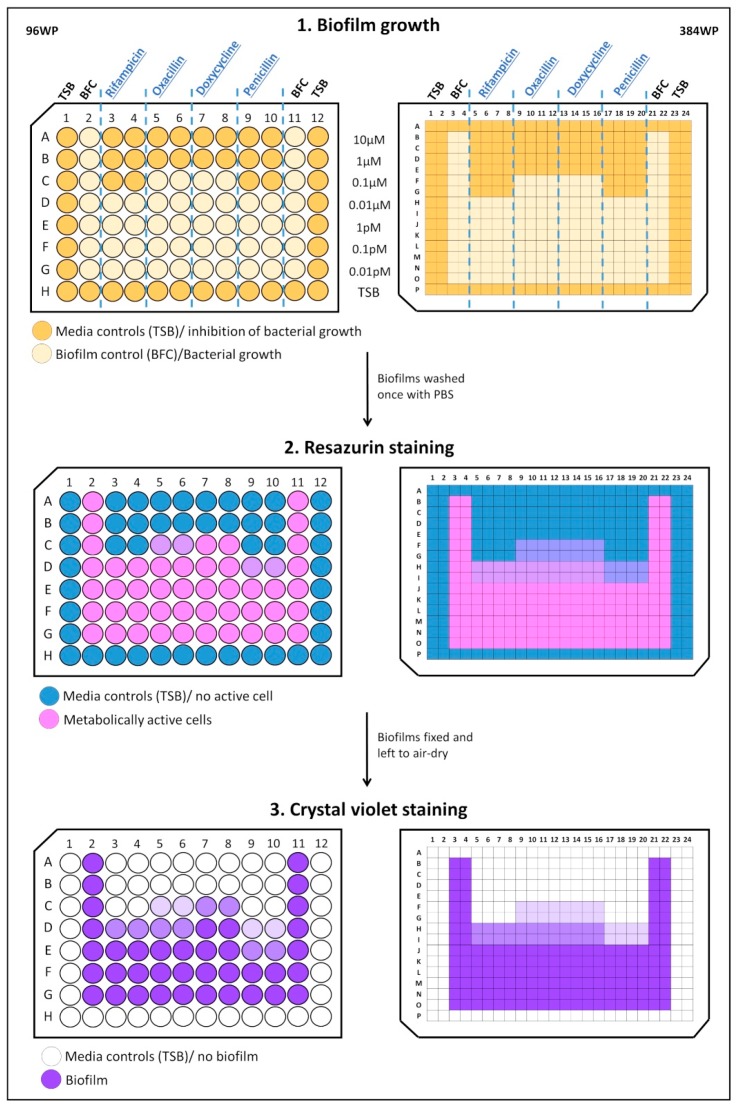
Workflow used for comparing the inhibition of *S. aureus* growth with a panel of four model antibiotics using the pre-exposure mode in 96WP and 384WP.

A small panel of four well-characterized antibiotics (penicillin, rifampicin, oxacillin, and doxycycline; Sigma–Aldrich, St. Louis, MO, USA), with diverse chemotypes and three different mechanisms of action, were tested against *S. aureus* (Scheme 1). For that purpose, the antibiotics were dissolved in dimethyl sulfoxide (DMSO) (34943, Sigma–Aldrich, St. Louis, MO, USA) to obtain a stock solution of 20 mM. The tested antibiotics were further dissolved in DMSO is such a way that the final concentration of DMSO did not exceed 2.5% (*v/v*). A pre-exposure mode was used in which the antibiotics (1 µL per well in 384WP and 5 µL per well in 96WP) were added into the wells at the same time with the bacterial suspensions. The following parameters were used: (i) the total working volume for 384WP was 40 µL and 200 µL for 96WP, (ii) for 384WP the added concentration of suspended cells was 7.00 × 10^4^ CFU mL^−1^, whereas a higher concentration of added suspended cells (4.00 × 10^6^ CFU mL^−1^) was used for 96WP. The inhibition capacity of the indicated antibiotics at different concentrations was measured after 18 h whlie using the resazurin (20 µM, 30 min.) and crystal violet assays, as described above.

### 3.6. Pilot Screening Against S. aureus Biofilms in 384WP and 96WP

A total of 100 compounds were tested on *S. aureus* biofilms grown in 96WP and 384WP platforms. Eighty-eight of them were from a natural derivatives collection (NDL-3000, TimTec Inc., Newark, DE, USA), two were provided by the medicinal chemistry group (Division of Pharmaceutical Chemistry and Technology, Faculty of Pharmacy, University of Helsinki, Helsinki, Finland), and were synthesized, as described in [56]. The remaining 10 were well-known antibiotics, included for control purposes. All of the compounds were dissolved in DMSO:PBS (1:1, *v/v*) to a concentration of 400 µM. One µL per well (384WP) or 5 µL per well (96WP) of compound (at a final concentration of 10 µM) were added to the test plates in a final working volume of 40 µL in 384WP and 200µl in 96WP. The screening was performed using the pre-exposure (antibiotics added at the same time as the bacteria, cells with compounds cultured for 18 h) and post-exposure (antibiotics added onto 18 h-old biofilms, cells with compounds incubated for 24 h) modes. The *S. aureus* inoculum concentration was 7.00 × 10^4^ CFU mL^−1^ in 384WP and 4.00 × 10^6^ CFU mL^−1^ in 96WP. The effect of the compounds was assessed while using the resazurin (20 µM in pre-exposure and 40 µM in post-exposure, 30 min. incubation at RT in darkness, 220 rpm) and crystal violet (0.023%, *v/v*), 5 min. incubation, 1 h dissolution in EtOH at RT) staining assays, as described above. The list of all tested compounds, sources, and their screening results in 96WP and 384WP is available in the Supplementary Table S1.

### 3.7. Screening of a Compound Library against S. aureus

The commercially available Microsource Spectrum Collection (Discovery Systems Inc., Gaylordsville, CT, USA) was screened on *S. aureus* biofilms that were grown in 384WP. The 2000 compounds were received as dry powders and solubilized in DMSO:PBS (1:1, *v/v*) to a concentration of 200 µM. Two µL per well of compound were added to the test plates in a final working volume of 40 µL (for a final concentration of 10 µM). As for the pilot screening, the screening was performed while using the pre-exposure and post-exposure modes and the *S. aureus* inoculum concentration was 7.00 × 10^4^ CFU mL^−1^. The wells were treated, as described in Section 2.6, using the resazurin and crystal violet staining assays to measure the effect on biofilms viability. The list of all tested compounds, sources and their screening results in 384WP is available in the Supplementary Materials (Table S2).

### 3.8. Statistical Analysis

The inhibition results of bacterial growth by antibiotics are shown as the percent of control ± S.D. (standard deviation). The assay performance was monitored using the screening window coefficient Z’ factor (Z’, Equation (1)) and the signal-to-noise (S/N, Equation (2)), calculated with the following equations, according to [36,57], respectively:

Z’ = (1 − (3 × s.d._max_ + 3 × s.d._min_))/(X_max_ − X_min_) (1)

S/N = (X_max_ − X_min_)/√(s.d._max_^2^ − s.d._min_^2^) (2)

X_min_ and s.d._min_ are the average and the standard deviation of the readings that were obtained in media controls (wells containing only sterile TSB) and X_max_ and s.d._max_ are the average and standard deviation of the readings obtained in biofilm controls (wells containing untreated bacteria).

## 4. Conclusions

In this work, we have miniaturized an antibacterial screening platform combining the resazurin and crystal violet staining into 384WP format while using two different bacterial species, *S. aureus* and *P. aeruginosa*, as gram-positive and gram-negative biofilm-forming models, respectively. The method was optimized in terms of initial bacterial cell concentrations, working volumes, the resazurin and crystal violet concentrations, and incubation times. Despite a slightly higher well-to-well variability detected in 384WP when compared to 96WP, we show that the miniaturized platform produces high quality results and that the resazurin staining and crystal violet staining can be easily applied to 384WP to study changes in bacterial biofilm formation. Through antibacterial challenges and a validatory small-scale pilot screen, we also showed that the obtained results are comparable to those obtained in 96WP, making this miniaturized platform suitable for the large-scale screening of antimicrobial compounds. A screening of 2000 compounds using this platform further confirmed its applicability. Although no new compounds worth taking further as a lead candidate were identified, interesting observations were made on many compounds that are not presently used as antibiotics, and our results were confirmative of earlier findings that were made during other screening studies. Other parameters, including the shaking speed during the incubation, which can affect biofilm formation and tolerance, were not studied here. The effect of the smaller working volume and the shape of the wells in the 384WP on the distribution of nutrients and oxygen and, thereby, the overall architecture of the biofilms, also remains to be studied. Nevertheless, our screening campaign, by indicating many previously identified compounds, strengthens the applicability of the optimized 384 platform in the reliable identification of new antibacterial compounds. In addition, this platform was shown to allow the testing of four-times the number of compounds in a single run as compared to 96WP with less amount of materials needed. In conclusion, the optimized 384WP platform demonstrates that resazurin and crystal violet can be applied for high-throughput anti-biofilm screening in 384WP.

## Supplementary Materials

Supplementary Materials can be found at https://www.mdpi.com/1422-0067/21/9/3034/s1. Table S1: Compounds identification and inhibition results of the pilot screening performed on *S. aureus* biofilms in 96WP and 384WP. Table S2: Identification of the compounds from the Microsource Spectrum Collection (Discovery Systems Inc., Houston, TX, USA) and results of the screening performed on *S. aureus* planktonic cells and biofilms in 384WP. Figure S1: Correlation of the Z’ factor and the signal-to-noise (S/N) statistical parameters throughout the optimization process. Figure S2: Folds of the Z’ factor and the Max-Min value obtained for the crystal violet assay after different incubation periods of the dye in the wells and different dissolution periods in ethanol.

## Abbreviations

ATCC	American Type Culture Collection
CFU	Colony-forming unit
DMSO	Dimethyl sulfoxide
HTS	High-throughput screening
MDPI	Multidisciplinary Digital Publishing Institute
MIC	Minimum inhibitory concentration
MoA	Mode of action
OD	Optical density
PBS	Phosphate buffered saline
Rpm	Revolutions per minute
RT	Room temperature
S.D.	Standard deviation
S/N	Signal-to-noise
TSA	Tryptic soy agar
TSB	Tryptic soy broth
96WP	96-well plate
384WP	384-well plate
Z’	Z’ factor

## Appendix A

**Table A1:** Hits preventing over 80% of *S. aureus* biofilm formation from the screening of 2000 compounds from the Microsource Spectrum Collection (Discovery Systems Inc.).

Compound Name	% Inhibition Biofilm Formation		Main Known Function ^1^
	Viability	Biomass	
CETYLPYRIDINIUM CHLORIDE	99	95	anti-infective
MINOCYCLINE HYDROCHLORIDE	95	92	antibacterial
ECONAZOLE NITRATE	94	92	antifungal
NOVOBIOCIN SODIUM	99	90	antibacterial
MECLOCYCLINE SULFOSALICYLATE	97	93	antibacterial
TENIPOSIDE	97	91	antineoplastic
AZLOCILLIN SODIUM	99	92	antibacterial
AMPHOTERICIN B	95	94	antifungal
LOMEFLOXACIN HYDROCHLORIDE	85	86	antibacterial
CEFAMANDOLE SODIUM	99	94	antibacterial
NAFCILLIN SODIUM	99	92	antibacterial
DACTINOMYCIN	101	96	antineoplastic
DIRITHROMYCIN	100	94	antibacterial
MITOMYCIN C	83	85	antineoplastic
RIFAXIMIN	98	88	antibacterial
CHLORHEXIDINE	82	86	antibacterial, disinfectant
NORFLOXACIN	99	94	antibacterial
OFLOXACIN	95	95	antibacterial
METHACYCLINE HYDROCHLORIDE	99	94	antibacterial
BENZETHONIUM CHLORIDE	101	95	anti-infective
CEPHALOTHIN SODIUM	99	88	antibacterial
SULCONAZOLE NITRATE	95	92	antifungal
GEMIFLOXACIN MESYLATE	97	93	antibacterial
THIMEROSAL	98	91	anti-infective, preservative
PENICILLIN G POTASSIUM	96	92	antibacterial
RIFAMPIN	101	95	antibacterial
TRIAMCINOLONE ACETONIDE	90	90	anti-inflammatory
HETACILLIN POTASSIUM	99	80	antibacterial
CHLORPHENIRAMINE (S) MALEATE	80	84	antihistaminic
PENICILLIN V POTASSIUM	98	88	antibacterial
CLINDAMYCIN HYDROCHLORIDE	99	97	antibacterial
HEXACHLOROPHENE	95	89	anti-infective
GENTIAN VIOLET	100	92	antibacterial, anti-helminthic
PYRVINIUM PAMOATE	99	94	anti-helminthic
MICONAZOLE NITRATE	88	90	antifungal
CHLORTETRACYCLINE HYDROCHLORIDE	96	89	antibacterial, anti-amebic
DICLOXACILLIN SODIUM	101	96	antibacterial
LINCOMYCIN HYDROCHLORIDE	92	89	antibacterial
CHLORTHALIDONE	98	86	diuretic, antihypertensive
PHYSOSTIGMINE SALICYLATE	99	86	cholinergic, miotic
CLOXACILLIN SODIUM	97	87	antibacterial
PHENYLMERCURIC ACETATE	99	95	antifungal
ERYTHROMYCIN ETHYLSUCCINATE	99	92	antibacterial
FURAZOLIDONE	97	94	antibacterial
TRIMETHOPRIM	85	85	antibacterial
FUSIDIC ACID	95	92	antibacterial
VANCOMYCIN HYDROCHLORIDE	95	96	antibacterial
PHENETHICILLIN POTASSIUM	100	85	antibacterial
ROXITHROMYCIN	99	94	antibacterial
MOXIFLOXACIN HYDROCHLORIDE	90	84	antibacterial
CEFTIBUTEN	100	93	antibacterial
BACAMPICILLIN HYDROCHLORIDE	100	98	antibacterial
TYLOSIN TARTRATE	99	96	antibacterial
RETINYL PALMITATE	100	95	provitamin
FLOXURIDINE	100	86	antineoplastic
CEFDINIR	101	98	antibacterial
SARAFLOXACIN HYDROCHLORIDE	93	84	antibacterial
TRICLOSAN	101	94	anti-infective
TELITHROMYCIN	100	88	antibacterial
NICLOSAMIDE	87	81	anthelmintic, teniacide
LEVOFLOXACIN	97	89	antibacterial
CIPROFLOXACIN	97	93	antibacterial, fungicide
TICARCILLIN DISODIUM	101	100	antibacterial
TILMICOSIN	101	101	antibacterial
GATIFLOXACIN	93	99	antibacterial
METHYLBENZETHONIUM CHLORIDE	99	91	anti-infective
AZITHROMYCIN	100	97	antibacterial
MUPIROCIN	100	97	antibacterial
PEFLOXACINE MESYLATE	99	95	antibacterial
TEICOPLANIN	99	94	antibacterial
ERYTHROMYCIN STEARATE	97	93	antibacterial
CETRIMONIUM BROMIDE	95	90	anti-infective
CLORGILINE HYDROCHLORIDE	99	87	antidepressant
CELASTROL	100	96	antineoplastic
FLORFENICOL	97	85	antibacterial
AGELASINE	100	96	antineoplastic
CARMOFUR	90	89	antineoplastic

^1^ Provided by Discovery Systems Inc.
