# Supplementary material for: Optimization of a High-Throughput 384-Well Plate-Based Screening Platform with Staphylococcus aureus ATCC 25923 and Pseudomonas aeruginosa ATCC 15442 Biofilms
Source: Int J Mol Sci. 2020 Apr 25;21(9):3034. doi: 10.3390/ijms21093034 (PMC7246797; doi:10.3390/ijms21093034)
Supplement: Supplementary file 1 [file ijms-21-03034-s001.zip › ijms-782205-SI/Supplementary files_Gilbert-Girard et al/Supplementary Table 1.pdf]

Supplementary Table 1. Identification and source of the compounds and results of the pilot screening performed on S. aureus biofilms in 96WP and 384WP. Results are expressed as the average percentage of inhibition from two biological replicates.

Compounds	ID number	Source	Inhibition of *S. aureus* biofilms (%)							
			Pre-exposure				Post-exposure			
			Viability		Total biomass		Viability		Total biomass	
			96WP	384WP	96WP	384WP	96WP	384WP	96WP	384WP
Rifampicin	R3501	Sigma-Aldrich	98.7	100.6	88.1	81.3	48.6	65.2	10.1	41.5
Oxacillin	28221	Sigma-Aldrich	99.8	100.6	96.9	97.2	25.5	61.7	21.9	52.1
Doxycycline	D9891	Sigma-Aldrich	99.1	100.4	88.8	91.2	34.0	45.1	-27.6	35.1
Penicillin	13752	Sigma-Aldrich	99.4	100.4	98.8	100.8	48.8	62.2	40.4	38.0
Streptomycin	56501	Sigma-Aldrich	36.5	100.0	37.5	100.1	3.1	8.5	6.8	-2.8
Tetracycline	87128	Fluka Analytical	99.2	100.7	90.7	101.3	31.0	50.7	-13.7	17.9
Ampicillin	A6140	Sigma-Aldrich	99.9	101.2	99.5	84.9	33.7	69.5	36.9	32.4
Dicloxacillin	D9016	Sigma-Aldrich	100.2	101.1	99.1	91.8	36.1	65.2	37.1	36.7
Levofloxacin	28266	Sigma-Aldrich	98.3	101.0	86.4	95.7	33.3	60.9	36.6	30.6
Vancomycin	861987	Sigma-Aldrich	98.4	101.3	95.6	100.8	13.1	14.7	23.6	12.2
N -(abiet-8,11,13-trien-18-oyl) cyclohexyl-L-alanine	11	Manner et al., 2015	42.8	21.0	6.7	-31.7	3.2	18.6	4.4	2.0
N-(abiet-8,11,13-trien-18-oyl)-D-tryptophan	9b	Manner et al., 2015	16.2	0.0	31.3	-39.7	5.2	29.1	-5.0	11.6
102	ST006037	NDL-3000	16.7	9.2	13.6	-8.7	14.5	31.8	14.0	29.0
103	ST006587	NDL-3000	12.4	11.5	1.3	-8.3	9.2	23.2	-7.0	28.4
104	ST006593	NDL-3000	9.6	12.6	2.6	7.3	-6.7	37.2	-12.4	27.1
105	ST007000	NDL-3000	6.7	4.0	-6.2	-19.7	0.5	8.3	14.1	29.2
106	ST007035	NDL-3000	5.1	8.5	-2.6	-0.9	7.0	14.6	-1.3	-4.8
107	ST007286	NDL-3000	11.1	7.9	23.7	-5.5	17.5	35.2	0.3	18.7
164	ST012939	NDL-3000	2.5	8.7	-35.1	-19.8	20.7	29.3	13.1	14.0
165	ST012940	NDL-3000	-2.5	7.3	-50.0	-16.2	-0.8	31.8	10.4	27.2
166	ST012949	NDL-3000	-0.9	15.7	-23.5	-16.5	0.7	33.1	4.2	22.9
167	ST012956	NDL-3000	0.1	2.2	-40.4	-8.3	3.6	17.6	-2.9	10.3
168	ST012957	NDL-3000	-1.4	6.9	-35.0	-24.7	4.8	9.5	-14.5	6.2
169	ST012960	NDL-3000	-1.0	-2.5	-39.3	-23.8	9.7	10.2	-1.1	10.4
170	ST013021	NDL-3000	10.0	11.0	-8.3	-15.8	5.2	27.1	13.3	26.2
1030	ST070457	NDL-3000	3.0	8.8	1.5	3.7	-4.2	32.1	-9.2	21.1
1031	ST070458	NDL-3000	12.3	12.2	26.6	-4.3	-3.7	35.3	-9.1	27.5
1032	ST070492	NDL-3000	6.5	9.2	22.9	-21.4	5.3	11.7	-0.8	8.6
1033	ST070499	NDL-3000	11.9	11.2	7.3	-2.2	3.9	14.2	-4.9	0.2
1034	ST070506	NDL-3000	8.3	17.2	19.5	11.3	10.7	17.6	5.3	26.8
1035	ST070595	NDL-3000	-0.4	4.7	-23.7	-3.4	12.4	15.0	-5.8	-3.2
1036	ST070604	NDL-3000	1.2	15.7	2.6	21.2	-2.4	32.9	-2.4	34.5
1037	ST070609	NDL-3000	4.4	15.7	14.2	-3.8	14.1	30.2	13.8	19.1
1038	ST070619	NDL-3000	1.6	9.9	15.7	-1.7	-2.1	9.8	-11.7	29.2
1039	ST070626	NDL-3000	-1.5	5.3	9.6	-24.3	8.4	20.6	-7.0	12.8
1040	ST070645	NDL-3000	8.3	10.2	31.2	-6.4	0.4	31.5	-2.7	13.1
1103	ST071169	NDL-3000	14.1	14.5	-3.7	-6.3	2.5	-4.3	-18.1	-9.6
1104	ST071178	NDL-3000	2.9	15.5	1.7	-2.8	-7.5	31.2	-16.3	29.0
1105	ST071180	NDL-3000	15.4	4.0	-11.3	-68.3	24.7	45.4	-4.2	18.5

1427	ST074775	NDL-3000	29.1	2.1	-13.9	-25.3	6.9	27.8	-26.2	13.9
1428	ST074776	NDL-3000	31.1	10.6	2.2	-8.4	8.3	14.5	-9.8	2.2
1429	ST074777	NDL-3000	29.7	12.8	16.7	5.1	-0.5	30.4	-10.5	-2.9
1430	ST074778	NDL-3000	9.8	21.0	21.8	1.0	23.9	33.3	-5.0	10.1
1431	ST074779	NDL-3000	10.0	17.2	0.3	12.7	12.2	27.0	-6.2	24.0
1432	ST074780	NDL-3000	15.9	16.0	-2.8	-20.7	5.2	36.4	-7.0	22.3
1433	ST074781	NDL-3000	5.5	13.5	-20.3	-2.0	0.7	21.4	-9.3	23.4
1434	ST074782	NDL-3000	10.0	-0.4	7.3	-9.2	-4.8	6.0	-14.2	-5.9
1435	ST074783	NDL-3000	12.8	7.7	10.0	0.0	4.2	19.0	-2.1	17.0
1436	ST074784	NDL-3000	12.6	10.5	-3.0	-7.2	-2.6	34.0	-7.7	25.9
1437	ST074785	NDL-3000	9.6	15.8	-5.1	-4.2	-4.4	34.9	-9.1	23.6
2084	ST081356	NDL-3000	7.1	20.4	18.1	0.0	-1.2	20.7	28.2	39.2
2085	ST081364	NDL-3000	0.0	-2.6	-0.1	-14.8	-10.8	7.1	6.3	16.2
2086	ST081380	NDL-3000	6.4	14.1	16.1	1.8	9.8	18.8	2.1	4.6
2280	ST088177	NDL-3000	2.1	12.0	25.6	1.3	5.4	26.0	3.9	26.9
2281	ST088185	NDL-3000	4.0	15.3	6.7	-12.6	0.1	38.9	2.8	23.6
2282	ST088217	NDL-3000	2.5	16.8	20.9	0.1	9.7	40.9	9.9	24.5
2283	ST088220	NDL-3000	6.1	21.8	2.4	-4.3	9.0	39.8	15.8	38.9
2284	ST088330	NDL-3000	-5.0	0.4	2.6	-20.4	2.4	29.6	9.3	24.3
2285	ST088333	NDL-3000	15.9	4.4	19.1	-15.0	2.0	13.6	-19.7	-22.5
2324	ST088606	NDL-3000	1.3	6.0	17.9	-13.3	0.0	31.1	-3.8	27.7
2325	ST088608	NDL-3000	6.3	10.2	12.0	-20.7	3.2	33.1	5.7	36.5
2326	ST088612	NDL-3000	8.3	9.8	18.6	-16.6	5.6	35.8	3.8	35.7
2409	ST092585	NDL-3000	-1.0	15.1	18.1	-16.3	13.5	31.5	-5.7	39.1
2410	ST092586	NDL-3000	-8.8	2.9	11.2	-26.5	5.3	11.2	6.6	26.1
2411	ST092587	NDL-3000	2.2	6.7	-4.0	-10.3	10.7	23.1	9.2	-7.9
2412	ST092601	NDL-3000	4.1	8.9	6.0	-15.7	4.9	34.4	19.0	17.6
2413	ST092612	NDL-3000	0.6	14.6	-8.6	-8.6	0.0	34.4	16.4	27.0
2414	ST092622	NDL-3000	16.2	11.0	10.9	-9.1	29.7	37.9	28.2	36.3
2415	ST092632	NDL-3000	17.4	21.6	-1.2	-3.0	18.4	42.2	17.9	39.8
2416	ST092652	NDL-3000	-6.9	6.5	14.6	-15.5	4.1	32.7	14.8	36.0
2417	ST092660	NDL-3000	5.1	10.4	13.8	8.2	-0.2	3.5	5.2	-5.0
2418	ST092677	NDL-3000	-5.5	10.6	25.1	-2.1	-0.4	25.0	4.4	25.4
2419	ST092685	NDL-3000	5.6	15.3	30.2	-2.6	6.6	31.6	21.8	15.0
2420	ST092688	NDL-3000	12.5	10.4	30.1	0.2	13.5	29.9	20.0	28.4
2421	ST092693	NDL-3000	13.1	12.5	17.0	-12.8	8.8	31.1	13.8	34.9
2422	ST092696	NDL-3000	-5.2	4.2	14.0	-8.5	6.6	7.3	5.2	17.0
2423	ST092700	NDL-3000	13.0	7.8	13.6	-13.7	-1.7	-11.4	4.9	-36.8
2424	ST092701	NDL-3000	4.1	11.4	12.0	-20.6	1.1	26.5	4.7	15.7
2425	ST092702	NDL-3000	13.4	17.3	19.6	-18.5	5.2	33.0	25.8	17.5
2465	ST092936	NDL-3000	21.4	18.0	29.5	-4.1	5.5	26.3	19.0	8.5
2466	ST092942	NDL-3000	14.4	18.0	27.1	-0.8	9.6	25.9	15.0	16.4
2467	ST092966	NDL-3000	2.5	7.5	22.5	-24.9	-6.1	22.7	-1.8	-5.7
2468	ST092971	NDL-3000	-5.2	7.7	-20.1	-55.0	-11.8	-1.1	-15.7	-35.7
2469	ST092972	NDL-3000	5.2	5.7	-18.8	-58.0	-14.3	9.8	-30.5	-10.8
2470	ST093057	NDL-3000	10.1	10.6	1.0	-5.9	3.6	11.0	23.9	20.8
2471	ST093063	NDL-3000	1.9	5.6	5.6	-26.3	0.1	22.8	11.9	12.9
2590	ST095411	NDL-3000	-5.1	14.5	7.2	-28.4	-1.9	25.5	12.7	21.5

2591	ST095412	NDL-3000	4.8	3.3	9.5	-12.3	-2.8	4.1	-4.0	15.5
2592	ST095413	NDL-3000	8.9	10.0	19.8	-1.1	-13.7	21.8	-9.5	-8.8
2593	ST095414	NDL-3000	-7.7	7.6	12.6	-13.3	-15.5	16.2	2.5	11.9
2594	ST095416	NDL-3000	-1.3	17.0	10.2	-6.5	-0.3	26.6	22.5	3.5
2595	ST095417	NDL-3000	-19.7	-2.3	-4.3	-22.5	-21.3	-18.1	-3.4	-22.1
2596	ST095418	NDL-3000	4.9	15.6	0.1	-21.4	-1.3	27.9	10.0	9.0
2597	ST095419	NDL-3000	-6.7	8.3	3.3	-15.1	-10.7	-1.2	1.8	12.8
2965	ST085690	NDL-3000	-2.3	-3.2	16.7	-36.6	-14.3	-1.1	-15.4	8.5
2966	ST085855	NDL-3000	-6.5	4.5	9.5	-31.9	-13.7	10.4	-10.1	20.2
2967	ST085897	NDL-3000	-3.7	5.3	18.1	-24.6	-3.4	13.4	21.5	33.2
2968	ST085899	NDL-3000	-5.3	8.6	18.1	-3.2	-5.0	11.1	13.4	20.7
2969	ST085955	NDL-3000	-4.9	11.2	0.5	-47.7	-16.8	20.6	0.2	20.9
2970	ST086020	NDL-3000	-11.6	-1.0	9.5	-47.3	-9.4	-1.8	-15.4	13.8
