# Supplementary material for: Optimization of a High-Throughput 384-Well Plate-Based Screening Platform with Staphylococcus aureus ATCC 25923 and Pseudomonas aeruginosa ATCC 15442 Biofilms
Source: Int J Mol Sci. 2020 Apr 25;21(9):3034. doi: 10.3390/ijms21093034 (PMC7246797; doi:10.3390/ijms21093034)
Supplement: Supplementary file 1 [file ijms-21-03034-s001.zip › ijms-782205-SI/Supplementary files_Gilbert-Girard et al/Supplementary Table 2.pdf]

Table S2. Identification of the compounds from the Microsource Spectrum Collection (Discovery Systems Inc.) and results of the screening performed on *S. aureus* planktonic cells and biofilms in 384WP.

Compounds name	Inhibition of *S. aureus* (%)[1]							
	Pre-exposure				Post-exposure			
	Planktonic cells		Biofilm		Planktonic cells		Biofilm	
	Turbidity	*Viability*	*Viability*	*Biomass*	*Turbidity*	*Viability*	*Viability*	*Biomass*
MAFENIDE HYDROCHLORIDE	9.9	8.6	-5.7	3.2	-5.3	30.7	-49.9	2.6
NEFOPAM	19.3	8.1	-21.3	18.5	52.3	-1.0	-26.5	4.3
METAPROTERENOL	28.7	12.3	-15.9	6.8	45.3	15.5	-46.6	21.7
SULPIRIDE	23.5	9.3	7.2	24.1	31.8	-3.5	12.4	3.1
METRONIDAZOLE	36.3	14.2	-14.9	-3.3	28.4	14.5	-9.6	-18.0
ESTRADIOL ACETATE	17.1	-23.5	-19.3	25.0	28.8	-27.4	-38.1	-26.7
NIACIN	33.9	8.8	-14.5	-1.0	29.2	10.2	-17.7	-5.6
LISINOPRIL	17.2	-26.7	12.5	6.2	32.4	-22.0	26.5	31.9
NOSCAPINE HYDROCHLORIDE	22.1	11.6	-0.8	-11.8	34.6	-8.0	30.1	8.2
SURAMIN	37.9	-17.4	-16.1	9.6	32.9	-23.4	-14.0	-22.7
PAPAVERINE HYDROCHLORIDE	25.0	1.9	-11.6	3.5	29.3	-3.3	0.8	19.8
PHENYLETHYL ALCOHOL	17.1	-38.9	-1.3	16.4	24.2	-19.5	5.2	-24.2
PHENYTOIN SODIUM	2.2	-9.5	-0.4	24.8	14.0	-15.5	10.7	0.8
EDROPHONIUM CHLORIDE	12.8	-43.6	2.1	29.1	9.5	-21.7	-16.8	18.6
PUROMYCIN HYDROCHLORIDE	22.1	22.1	-10.2	-3.0	-20.3	18.4	-47.1	-2.7
SULFADOXINE	49.3	3.6	-13.4	-12.0	36.6	10.7	-28.9	1.4
POTASSIUM p-AMINOBENZOATE	53.0	30.3	-15.2	-5.4	48.1	30.8	-20.7	2.6
PHENYL AMINOSALICYLATE	41.1	6.3	-11.7	-17.5	36.3	12.2	7.4	0.2
PIRACETAM	49.2	27.8	-12.6	-12.2	35.9	21.3	1.6	-12.0
IVERMECTIN	23.5	-5.3	-10.8	-10.7	40.1	10.3	-12.2	5.4
SENNOSIDE A	50.1	17.4	-2.7	-20.7	42.2	26.8	-23.9	-11.8
ADENOSINE	32.5	-7.9	6.3	-7.8	33.0	4.3	29.2	20.5
METAXALONE	30.8	25.1	-7.7	-10.6	32.4	6.5	25.9	6.8
AMODIAQUINE DIHYDROCHLORIDE	40.5	9.1	-10.6	-20.8	49.4	14.4	7.9	16.3
PROPOFOL	49.9	20.6	-16.5	-22.1	30.3	11.2	12.3	3.8
ATROPINE SULFATE	30.6	-19.4	-6.9	-17.7	43.8	-6.4	-4.2	-12.3
RANOLAZINE	31.3	7.1	-1.4	-15.9	27.8	0.3	15.2	0.9
beta-CAROTENE	12.9	-28.7	6.1	-5.0	-9.7	-15.3	3.7	18.4
LEVALBUTEROL HYDROCHLORIDE	13.1	5.5	-10.8	-8.3	0.2	10.3	-24.5	-6.5
CARBACHOL	28.3	11.1	-2.2	-16.4	40.0	10.1	-19.6	24.4
LEVODOPA	69.7	55.0	-29.2	-21.6	72.6	53.3	-46.8	-13.6
CETYLPYRIDINIUM CHLORIDE	99.7	97.8	98.6	95.1	43.6	18.9	0.6	-1.5
MAPROTILINE HYDROCHLORIDE	49.6	9.6	-6.7	-19.6	43.2	22.0	2.8	4.6

PIRENZEPINE HYDROCHLORIDE	48.7	19.5	-7.9	-21.3	45.9	24.2	-19.6	15.6
METHACHOLINE CHLORIDE	50.0	26.3	-12.4	-18.3	33.9	18.3	1.5	1.0
RANITIDINE	35.0	18.0	5.8	-17.9	33.9	12.9	31.1	35.4
MINOCYCLINE HYDROCHLORIDE	98.9	97.4	94.8	92.0	74.0	66.6	41.8	-11.2
ECONAZOLE NITRATE	99.7	93.9	94.4	92.4	66.7	-15.1	-4.8	14.1
NIFEDIPINE	55.7	20.7	-16.9	-17.0	36.5	1.4	9.7	8.9
BUMETANIDE	36.1	13.3	-9.2	-22.6	41.1	2.4	17.3	21.9
NOVOBIOCIN SODIUM	99.4	96.4	99.3	89.6	77.7	55.4	48.9	7.4
CEFUROXIME SODIUM	31.0	-25.2	26.3	13.6	34.9	-24.6	25.2	34.9
PARACHLOROPHENOL	23.4	8.5	4.4	21.1	-10.1	18.0	9.0	21.8
MECLOCYCLINE SULFOSALICYLATE	98.8	98.1	97.1	93.3	96.2	91.7	11.8	-27.4
FENOFIBRATE	50.8	13.2	-2.7	-15.2	52.4	14.4	-19.5	-1.8
THIOPENTAL SODIUM	48.7	7.0	-19.0	-22.8	33.8	22.3	10.0	10.7
MEBENDAZOLE	41.6	23.6	-5.4	-23.0	40.6	25.9	4.0	11.4
FINASTERIDE	55.4	26.5	-17.1	-25.1	36.2	15.8	-1.1	-13.3
BETAMETHASONE VALERATE	51.1	31.3	-16.3	-24.6	32.8	19.3	-6.5	0.0
TESTOSTERONE	44.5	-11.2	0.8	-21.1	29.8	-6.1	26.8	13.9
GLUCONOLACTONE	40.9	8.5	5.3	-18.8	31.9	10.3	32.3	26.9
SODIUM NITROPRUSSIDE	53.5	-8.7	-15.3	-21.8	37.6	3.3	0.2	-20.3
TENIPOSIDE	98.0	97.4	96.9	91.4	65.8	35.9	12.3	-12.0
ALLOPURINOL	52.5	-5.1	-15.7	-23.1	43.1	8.5	5.9	17.2
CHLOROGUANIDE HYDROCHLORIDE	23.7	-4.4	-2.0	-17.4	33.3	-9.4	6.9	23.5
AMOXICILLIN	27.6	-14.2	4.3	-5.2	14.9	-14.3	16.0	43.0
GRISEOFULVIN	14.8	10.7	-2.7	4.6	-1.7	8.5	-0.7	24.8
AUROTHIOGLUCOSE	53.5	9.4	-7.3	-20.6	72.0	48.8	-46.3	-15.4
DANTHRON	59.1	43.1	-17.8	-18.8	53.0	21.5	-27.4	13.6
BETAMETHASONE	55.1	21.2	-15.7	-23.1	49.8	24.8	21.2	1.0
METFORMIN HYDROCHLORIDE	48.7	34.1	-2.8	-20.4	41.8	18.2	1.4	16.7
CARBAMAZEPINE	61.2	34.6	-15.7	-23.1	54.1	30.8	-27.2	-0.2
RUTOSIDE (rutin)	55.9	33.7	-12.6	-20.1	43.5	26.2	-9.3	11.6
CHLORAMBUCIL	38.0	23.1	14.0	-2.9	34.2	2.9	24.5	3.5
MECAMYLAMINE HYDROCHLORIDE	49.6	23.5	9.0	-2.7	37.0	10.5	37.0	4.4
PRAMOXINE HYDROCHLORIDE	65.5	15.0	-11.2	-21.4	39.1	-1.1	11.0	5.3
METHIMAZOLE	43.4	10.6	-13.1	-16.0	39.5	10.1	15.1	20.1
SULOCTIDIL	49.4	19.8	9.0	-21.9	35.8	4.3	-2.6	17.1
MINOXIDIL	23.0	11.5	-9.6	-12.3	42.5	9.2	12.9	21.5
FLUNISOLIDE	31.7	-17.2	18.9	9.2	31.0	-15.3	5.2	27.2
NITROFURANTOIN	30.6	0.4	17.4	10.1	4.1	18.3	-10.0	18.6
CARBENOXOLONE SODIUM	49.4	24.2	-0.2	-13.5	60.4	40.0	-18.9	-0.4
NYLIDRIN HYDROCHLORIDE	48.3	35.2	-5.2	-17.6	53.2	33.2	-28.1	-21.2

VIGABATRIN	56.0	28.7	-1.1	-20.3	44.3	22.6	-0.5	-1.8
PARGYLINE HYDROCHLORIDE	51.9	36.4	-13.6	-18.8	36.5	31.6	1.8	7.8
RIBOFLAVIN	62.7	37.5	-5.5	-22.8	48.8	26.3	-20.9	-0.3
FENOPROFEN	49.4	31.7	-7.6	-22.1	41.3	33.1	-12.4	-24.9
CLOMIPRAMINE HYDROCHLORIDE	43.8	21.2	13.4	-7.0	34.8	12.5	25.6	8.4
NALBUPHINE HYDROCHLORIDE	37.1	25.6	4.7	-7.1	43.9	20.7	40.9	-14.4
PENTETIC ACID	56.9	23.3	-14.2	-20.6	25.9	-0.5	16.8	10.6
ERYTHROMYCIN	98.3	97.9	99.3	37.9	77.5	65.2	39.3	-28.9
SANGUINARINE SULFATE	49.6	19.9	0.9	-18.2	31.8	-0.2	-22.4	-27.0
AZLOCILLIN SODIUM	97.6	96.8	99.4	92.4	79.9	42.7	57.5	1.8
SODIUM OXYBATE	25.1	-12.0	8.8	11.8	20.9	-8.8	3.8	10.3
TANNIC ACID	38.6	21.2	-1.8	-13.9	36.5	16.5	19.8	-25.2
ALVERINE CITRATE	-9.9	27.1	11.5	15.9	22.2	50.3	-119.1	-29.7
CLARITHROMYCIN	46.1	19.4	-4.4	-20.0	50.0	24.9	0.1	7.4
AMPHOTERICIN B	98.5	98.4	95.5	93.6	96.1	92.8	8.4	-9.6
BENAZEPRIL HYDROCHLORIDE	64.4	48.3	1.8	-15.6	41.6	31.3	-13.9	-21.9
AZATHIOPRINE	57.5	28.1	-13.9	-20.7	44.1	23.4	-8.2	0.5
ACEDAPSONE	57.4	36.4	-0.3	-21.6	49.1	36.1	-14.7	8.1
BETHANECHOL CHLORIDE	45.5	31.3	0.3	-15.8	43.9	10.0	29.4	23.1
PREGABALIN	55.8	36.4	13.3	-8.2	38.4	12.2	23.4	10.1
CARBENICILLIN DISODIUM	57.7	2.9	-12.3	-22.3	38.6	5.5	39.3	21.0
ZOMEPIRAC SODIUM	63.0	22.8	-6.2	-16.2	38.5	8.6	28.1	21.6
CHLORAMPHENICOL PALMITATE	40.1	-9.7	-10.5	-15.7	45.4	0.5	6.3	18.8
MECHLORETHAMINE	59.1	10.8	-0.6	-14.7	42.8	13.6	2.9	7.8
MEPHENESIN	45.8	-6.1	4.8	-2.6	11.1	-18.1	7.4	21.2
METHOCARBAMOL	-6.4	2.1	31.3	25.3	9.0	28.1	-15.2	-13.5
RONIDAZOLE	62.7	15.8	-13.8	-6.2	65.9	38.4	-26.8	-20.0
MOXALACTAM DISODIUM	41.1	14.7	-2.0	-19.4	66.4	53.3	-24.4	-14.1
FLUMETHASONE	52.7	30.2	3.0	-9.2	45.7	21.3	-1.2	-26.6
OXYBUTYNIN CHLORIDE	50.5	38.6	5.0	-20.9	49.6	30.5	-21.3	-22.1
FOLIC ACID	46.6	18.2	-8.3	-22.7	51.3	28.9	-8.8	-7.6
NYSTATIN	31.0	17.2	5.1	-21.0	44.7	32.7	-21.0	0.9
LOMEFLOXACIN HYDROCHLORIDE	98.5	96.2	84.6	85.9	81.6	60.1	36.5	2.7
PENICILLAMINE	29.7	-0.1	12.2	-8.2	42.1	18.8	17.5	7.2
ACEBUTOLOL HYDROCHLORIDE	56.1	24.7	-11.4	-17.5	37.5	5.4	2.0	20.6
FLUFENAMIC ACID	57.9	55.1	-26.9	-20.0	48.4	35.8	-26.2	-16.3
PHENFORMIN HYDROCHLORIDE	42.6	13.6	-2.3	-15.5	40.8	3.2	7.0	21.7
PROGLUMIDE	28.8	-4.7	-1.4	-19.1	40.9	20.5	14.0	17.1
PROSCILLARIDIN	20.7	-3.1	15.3	-14.2	27.7	-17.6	13.5	23.8
PROMETHAZINE HYDROCHLORIDE	23.7	15.9	5.1	-14.1	4.4	11.5	-6.4	-19.3

alpha-TOCHOPHEROL	47.7	25.0	-0.9	-11.9	60.5	48.7	-7.9	7.3
CHOLINE CHLORIDE	74.3	57.5	-14.5	-21.9	63.1	49.9	-21.9	-14.9
ETHYL PARABEN	44.9	14.7	6.0	-18.1	42.7	26.3	3.3	7.5
CARPROFEN	61.0	65.2	-38.9	-22.2	29.9	30.8	-50.8	-22.0
AMANTADINE HYDROCHLORIDE	47.4	15.6	1.4	-21.9	51.6	34.8	-11.0	13.3
HYDROQUINONE	61.4	39.2	-5.8	-23.2	52.4	41.5	-12.5	-24.1
AMPICILLIN SODIUM	42.2	-3.2	13.4	-17.8	41.5	13.5	28.3	-1.2
VALSARTAN	61.2	42.0	2.0	-16.0	42.1	17.3	22.9	-28.0
BACITRACIN	54.9	16.8	1.3	-22.0	47.3	5.9	15.6	-0.2
ATOMOXETINE HYDROCHLORIDE	60.1	26.0	-6.7	-23.0	46.4	17.6	12.7	-28.4
BISACODYL	38.5	2.3	-0.1	-21.1	44.2	12.3	15.0	16.3
TOPOTECAN HYDROCHLORIDE	37.9	16.9	1.4	-19.1	38.7	11.9	29.5	-28.1
CARBINOXAMINE MALEATE	26.2	-7.0	15.8	-7.3	12.7	-15.2	16.3	21.8
SPARTEINE SULFATE	30.2	14.6	-4.7	-6.2	3.4	44.3	-21.9	-13.7
CHLORAMPHENICOL HEMISUCCINATE	62.9	49.4	6.8	-16.8	70.1	55.4	-37.0	-21.4
MECLIZINE HYDROCHLORIDE	75.7	59.0	-17.4	-19.9	63.3	52.3	-28.5	-27.8
SULFACHLORPYRIDAZINE	82.2	55.2	15.9	-18.6	47.6	29.0	-21.1	-28.3
METHOTREXATE(+/-)	71.7	57.0	4.2	-21.9	49.3	45.7	-26.3	-25.9
SULFAMETER	61.7	26.8	1.9	-21.4	57.6	37.3	-23.2	-22.6
NADIDE	61.6	39.7	-1.1	-24.5	57.9	46.6	-25.2	-25.1
XYLAZINE	46.0	20.8	14.3	-15.0	41.0	19.1	18.7	-1.8
NOREPINEPHRINE	40.1	25.6	9.2	-22.3	46.1	22.8	13.3	-26.0
PHTHALYLSULFATHIAZOLE	52.7	32.0	-3.4	-19.5	41.0	12.6	19.8	7.0
ORPHENADRINE CITRATE	58.6	34.2	-0.7	-16.1	34.9	19.1	9.0	10.6
CEFAMANDOLE SODIUM	98.3	95.7	99.0	94.4	88.7	49.2	48.1	14.5
PHENACEMIDE	41.7	-6.5	-5.2	-18.1	40.4	18.2	5.0	13.3
ASPARTAME	25.8	-11.4	23.6	20.8	22.0	-6.7	9.7	27.5
FENBENDAZOLE	28.3	8.1	-11.0	-17.4	-3.5	10.3	-23.2	1.5
PENFLURIDOL	89.5	77.4	49.4	34.1	49.6	56.8	-13.5	-31.1
MINAPRINE HYDROCHLORIDE	54.1	37.6	-5.9	-22.1	58.2	43.0	-19.6	-24.7
HYDROCORTISONE VALERATE	51.0	25.4	6.3	-16.5	47.4	31.1	-16.4	-11.4
SCOPOLAMINE HYDROBROMIDE	54.5	46.7	6.7	-12.5	43.3	33.4	-3.2	-28.0
alpha-TOCHOPHERYL ACETATE	43.2	14.0	10.5	-22.3	49.5	30.2	-12.7	-12.3
ATORVASTATIN CALCIUM	60.8	39.6	-0.8	-23.0	51.6	41.9	-15.8	-28.4
COUMARIN	38.6	10.0	13.9	-8.5	39.9	11.5	15.1	-21.5
HYDROXYCHLOROQUINE SULFATE	43.4	21.3	18.0	-23.1	42.5	16.4	22.3	3.6
AMIKACIN SULFATE	63.6	27.7	-2.1	-18.2	37.8	8.5	11.4	15.7
NATEGLINIDE	59.7	31.3	-7.1	-10.4	49.7	20.2	28.2	8.6
AMPROLIUM	34.2	-19.2	-2.9	-10.8	39.1	9.0	1.3	7.0
SALSALATE	31.0	1.5	21.2	-16.0	36.0	25.7	26.8	-7.7

BACLOFEN	33.6	-14.2	7.0	-8.4	3.3	-16.2	4.0	16.7
DESOXYCORTICOSTERONE ACETATE	26.1	20.2	0.6	-16.6	-1.2	4.9	-5.1	1.7
BITHIONATE SODIUM	91.3	84.6	65.8	33.1	87.5	62.4	66.6	21.0
PHENOXYBENZAMINE HYDROCHLORIDE	64.9	52.8	1.6	-19.0	53.5	32.2	-16.9	-12.3
CARISOPRODOL	45.8	28.2	17.1	-18.3	43.1	25.8	3.3	-1.0
TESTOSTERONE PROPIONATE	37.1	26.2	5.8	-21.0	36.3	12.4	3.0	-0.1
CHLORAMPHENICOL	86.9	74.9	29.1	-20.4	34.6	23.4	-11.6	-2.6
MECLOFENAMATE SODIUM	63.8	65.7	-0.9	-22.8	40.4	32.4	-12.4	1.7
SULFADIMETHOXINE	51.0	31.2	38.3	-10.8	37.6	13.0	27.7	3.5
METHOXAMINE HYDROCHLORIDE	41.4	36.3	16.1	-18.7	43.2	17.4	18.2	-14.6
SULFAMETHOXYPYRIDAZINE	37.7	11.2	20.0	-16.9	32.9	14.0	10.8	16.8
NAFCILLIN SODIUM	97.5	98.1	99.5	91.7	91.9	72.3	53.2	10.9
TOLAZAMIDE	48.5	7.4	7.7	-19.3	38.8	14.8	8.0	20.2
NORETHINDRONE	33.1	14.3	7.7	-15.1	25.3	26.7	3.2	9.3
SUCCINYLSULFATHIAZOLE	44.9	-7.1	22.5	-0.8	30.5	-0.7	16.4	22.3
OXACILLIN SODIUM	97.0	97.3	83.0	78.2	33.8	61.2	34.4	1.9
CEFMETAZOLE SODIUM	61.2	22.6	0.0	-21.9	42.3	20.4	-11.7	1.3
PHENAZOPYRIDINE HYDROCHLORIDE	47.1	44.3	0.8	-23.6	58.5	47.9	-20.1	-18.0
VARDENAFIL HYDROCHLORIDE	60.0	33.6	5.7	-19.2	40.2	24.1	-7.6	-5.6
FENSPIRIDE HYDROCHLORIDE	39.1	35.0	11.9	-22.7	40.7	31.5	-2.1	-4.6
TILORONE	56.0	28.0	8.2	-21.7	42.7	24.7	-6.3	3.4
MEMANTINE HYDROCHLORIDE	27.9	19.4	14.6	-11.3	39.3	33.9	6.5	16.0
REPAGLINIDE	38.8	22.2	17.7	1.6	29.5	19.4	31.1	0.1
THEOPHYLLINE	25.3	18.7	12.2	-19.9	37.6	22.6	31.6	16.8
DACTINOMYCIN	98.4	97.7	101.0	95.8	90.1	80.9	50.2	6.8
OXYPHENCYCLIMINE HYDROCHLORIDE	54.8	13.2	-0.6	-20.4	37.3	7.8	15.2	12.9
ACETAMINOPHEN	39.4	2.9	-1.2	-9.9	35.0	16.9	11.9	6.4
DIRITHROMYCIN	97.4	95.9	99.9	94.4	67.0	62.9	-3.4	-5.1
AMILORIDE HYDROCHLORIDE	47.0	-6.4	8.8	-8.7	19.5	-6.2	20.7	26.7
CANDESARTAN CILEXTIL	26.8	6.7	-9.8	-20.4	1.0	-5.1	-1.9	-7.7
ANTAZOLINE PHOSPHATE	41.9	14.2	8.1	-17.7	32.3	15.6	-10.1	-12.5
HYDROCORTISONE	49.7	49.9	6.2	-22.1	37.0	29.9	-5.1	-7.7
BECLOMETHASONE DIPROPIONATE	31.8	2.6	21.9	-1.1	23.4	19.2	17.3	-27.5
TRAMADOL HYDROCHLORIDE	40.3	35.6	16.3	-17.8	33.2	29.1	2.1	-12.8
BROMOCRIPTINE MESYLATE	52.8	27.3	12.1	-19.9	33.1	18.8	-11.2	1.4
PINACIDIL	53.9	34.5	5.4	-21.3	30.2	23.5	4.0	0.4
CEFADROXIL	53.5	11.6	18.8	-16.0	44.4	21.5	29.4	-6.6
METHIMAZOLE	39.9	11.0	21.4	-9.4	36.9	20.5	21.6	-29.2
CHLORCYCLIZINE HYDROCHLORIDE	63.6	5.3	3.2	-17.5	38.4	15.6	7.1	3.8
MEDRYSONE	47.9	10.7	10.5	-19.4	38.5	16.8	7.2	-20.7

SULFAGUANIDINE	62.1	11.4	2.2	-18.3	39.6	20.4	23.5	-3.5
METHYLDOPA	30.6	-13.7	25.2	-9.6	48.6	29.7	17.0	-0.4
SUPROFEN	51.3	3.1	0.2	-12.7	16.6	-2.4	-2.3	13.0
NALOXONE HYDROCHLORIDE	11.6	9.2	7.4	-6.7	-4.6	8.2	7.2	-3.1
GALANTHAMINE HYDROBROMIDE	51.9	31.1	6.1	-16.6	38.3	17.2	-15.3	-15.4
NORETHYNODREL	54.7	50.0	10.1	-22.3	40.4	32.7	-6.5	-31.2
TRANEXAMIC ACID	45.6	25.0	10.1	-17.1	35.9	17.5	14.3	9.5
OXYBENZONE	41.7	38.1	22.1	-22.5	39.1	33.2	-13.5	-10.1
CEFOPERAZONE SODIUM	86.9	70.1	13.6	-21.0	88.0	79.6	12.6	9.7
PHENELZINE SULFATE	53.7	41.7	13.1	-22.1	35.0	26.6	-16.2	-26.9
FLUORESCEIN	35.6	8.4	8.4	-17.4	38.3	17.4	20.9	-16.7
MEFENAMIC ACID	36.9	46.6	0.8	-17.0	26.5	21.9	9.9	-25.7
VINCRISTINE SULFATE	47.5	-0.3	1.5	-4.5	36.4	16.4	1.8	22.5
ATENOLOL	55.3	32.0	6.2	-12.6	37.8	22.1	14.0	-27.5
CROTAMITON	45.0	-0.9	10.8	-11.1	39.5	13.2	-4.3	5.9
TOLNAFTATE	32.6	-5.4	19.0	-11.0	29.4	7.5	1.7	-16.0
MITOMYCIN C	98.2	95.2	82.8	84.9	60.8	24.9	-0.9	7.2
PROPAFENONE HYDROCHLORIDE	19.5	21.3	-12.3	-12.8	2.4	1.5	-9.7	2.0
ACETAZOLAMIDE	80.4	55.6	-4.1	-20.3	36.8	23.5	-30.3	-23.2
MEPIVACAINE HYDROCHLORIDE	61.2	56.1	8.4	-21.8	33.4	30.1	-11.3	-9.4
AMINOCAPROIC ACID	57.6	28.6	29.6	-6.4	36.6	17.9	-17.5	-5.8
ROSIGLITAZONE	62.8	49.6	14.4	-13.3	44.0	39.2	-19.7	-26.9
ANTHRALIN	57.4	31.9	3.0	-21.7	45.7	22.7	-9.5	-14.2
RIFAXIMIN	97.1	97.7	97.8	88.4	89.1	83.4	33.1	3.4
BENSERAZIDE HYDROCHLORIDE	32.1	8.2	26.1	-6.8	41.9	19.9	33.4	6.0
TERBINAFINE HYDROCHLORIDE	27.7	15.1	19.7	-9.5	35.8	14.4	15.2	3.5
BUSULFAN	50.9	12.1	19.9	-20.8	47.3	22.4	9.8	-1.5
VERAPAMIL HYDROCHLORIDE	38.5	12.6	30.3	-0.9	40.2	28.0	13.6	19.4
CEFOTAXIME SODIUM	46.3	6.9	28.6	-11.5	56.8	24.8	37.0	9.6
ENILCONAZOLE	26.4	14.4	15.2	-4.5	34.7	10.5	2.9	-0.6
CHLORHEXIDINE	95.1	93.3	82.2	86.3	40.5	41.2	32.3	8.4
MEGESTROL ACETATE	17.1	17.9	-2.0	-11.3	-1.6	20.3	-6.8	3.4
SULFAMONOMETHOXINE	40.8	18.2	11.9	-18.0	44.0	31.1	-28.0	-30.3
METHYLPREDNISOLONE	70.6	58.8	0.7	-22.6	57.1	44.1	-48.9	-24.4
SACCHARIN	41.1	17.3	17.9	-21.4	36.0	23.5	-7.1	6.2
NAPHAZOLINE HYDROCHLORIDE	46.2	36.9	14.4	-16.7	32.4	32.3	2.3	2.6
FENRETINIDE	46.6	-6.6	18.1	-0.8	42.7	15.7	-28.1	-10.6
NORFLOXACIN	99.8	99.0	99.3	94.5	89.2	77.5	17.8	3.7
CEPHALEXIN	44.7	10.6	35.7	12.1	47.6	21.4	31.6	3.3
OXYMETAZOLINE HYDROCHLORIDE	41.9	37.8	6.3	-8.9	32.2	21.0	24.8	-4.7

OFLOXACIN	97.3	96.4	95.5	94.7	91.0	66.2	31.9	-2.8
PHENINDIONE	45.2	-6.0	19.5	-16.1	35.0	15.7	1.0	7.1
NIACINAMIDE	43.5	12.6	11.6	-9.0	39.7	15.1	-2.9	7.0
METHACYCLINE HYDROCHLORIDE	98.8	96.9	98.6	93.9	79.1	75.3	16.5	-16.8
OMEPRAZOLE	34.9	-17.4	12.1	10.3	6.0	-9.3	12.5	6.4
CARBETAPENTANE CITRATE	17.9	7.1	6.2	-16.2	-1.0	12.0	-14.5	1.5
CEFPROZIL	40.2	25.3	29.8	-3.2	56.6	40.6	-2.8	-22.5
TRIMETHOBENZAMIDE HYDROCHLORIDE	61.9	55.4	4.3	-19.3	35.8	36.1	-12.3	-26.9
DICHLORVOS	41.5	21.2	21.8	-11.8	35.8	29.0	-6.9	15.2
FLUCONAZOLE	56.2	52.5	10.6	-18.9	33.6	31.1	14.4	-24.9
ACETOHYDROXAMIC ACID	47.5	-7.6	18.6	-21.5	35.4	11.8	-8.0	2.2
NILUTAMIDE	38.0	-8.6	17.0	-16.0	29.0	24.1	-9.4	-17.7
AMINOGLUTETHIMIDE	34.3	19.3	26.4	-14.7	34.4	19.5	22.9	1.8
LOSARTAN	37.6	17.0	20.5	-13.4	27.4	23.5	32.1	-25.1
ANTIPYRINE	46.8	3.9	20.1	-23.1	36.9	14.5	1.2	5.8
CANRENONE	35.0	17.9	-0.5	-12.1	30.8	20.2	22.7	-11.6
BENZETHONIUM CHLORIDE	99.8	97.0	100.8	95.1	56.4	27.3	21.9	10.7
TOPIRAMATE	30.1	-20.0	10.3	-9.4	30.4	19.2	4.6	17.2
CAFFEINE	32.3	-15.0	25.8	9.8	3.2	8.6	10.2	-8.8
PANTOPRAZOLE	14.9	12.0	-1.2	-12.7	0.4	9.1	-22.8	-2.2
CEPHALOTHIN SODIUM	98.5	97.3	98.9	87.6	94.4	73.1	15.6	2.0
FIROCOXIB	39.8	23.2	15.1	-14.0	50.7	38.5	-28.4	-17.3
CHLOROCRESOL	27.0	-1.8	19.6	-7.1	35.0	21.5	2.1	-7.6
MELPHALAN	24.9	27.4	26.4	-3.5	33.7	31.0	-8.2	-26.5
SULCONAZOLE NITRATE	99.6	92.5	94.8	91.9	64.6	19.3	-18.6	3.9
METOCLOPRAMIDE HYDROCHLORIDE	47.8	4.7	6.8	-21.2	37.5	23.8	-12.7	-9.9
ACETANILIDE	22.8	15.8	23.9	-8.3	17.7	20.1	37.4	-25.4
NAPROXEN(+)	28.2	26.7	25.6	4.8	31.5	18.4	3.8	-2.2
ENALAPRIL MALEATE	49.9	13.9	17.1	-16.1	37.8	17.4	-7.6	-7.6
NORGESTREL	40.0	22.9	12.0	-10.7	34.1	23.7	8.5	3.0
OXOLINIC ACID	55.3	-7.4	12.3	-16.8	44.4	16.6	-9.9	2.1
OXYPHENBUTAZONE	29.4	8.6	6.9	-18.4	36.4	27.3	-1.7	9.9
BEZAFIBRATE	21.1	-16.1	22.4	23.6	34.2	11.6	7.8	-14.1
PHENIRAMINE MALEATE	25.8	14.7	-2.4	-20.3	-16.0	57.5	-26.8	11.0
PROPRANOLOL HYDROCHLORIDE (+/-)	18.2	1.9	17.6	-14.2	41.4	18.8	-27.9	-24.9
MEFEXAMIDE	48.2	47.2	9.8	-19.3	61.5	50.6	-28.0	-26.4
ZOLMITRIPTAN	30.4	12.0	22.3	2.4	32.8	17.7	-5.6	-26.9
PIMOZIDE	49.1	33.0	21.3	-18.5	31.1	21.9	-11.8	-27.2
METHYLDOPATE HYDROCHLORIDE	25.4	-6.7	19.7	-17.9	38.2	24.1	-23.0	-27.8
VINBLASTINE SULFATE	17.2	2.9	11.1	-18.9	46.1	35.5	-25.3	-9.3

TEMEFOS	20.0	15.2	25.8	9.2	38.1	19.2	-4.8	-12.4
LOVASTATIN	32.3	25.8	14.5	-8.6	38.9	19.7	1.6	-27.8
ACETYLCHOLINE	40.5	6.7	12.5	-12.2	36.3	12.2	7.9	-29.0
AMINOLEVULINIC ACID HYDROCHLORIDE	26.8	16.4	11.5	-20.0	36.5	18.3	8.2	-19.4
AMINOSALICYLATE SODIUM	33.5	-8.8	15.2	-16.9	34.4	9.2	16.7	7.9
HOMOSALATE	20.5	8.3	9.9	-9.2	34.9	18.7	-15.6	-19.2
APOMORPHINE HYDROCHLORIDE	31.1	-11.4	21.5	0.9	-1.1	12.6	-28.5	-13.7
MODAFINIL	-9.0	30.0	10.0	-13.4	-4.5	-2.3	-20.3	-2.9
BENZOCAINE	40.6	18.1	11.5	-16.1	45.7	27.2	-25.5	22.0
GEMIFLOXACIN MESYLATE	99.4	98.5	97.4	93.4	85.4	73.7	29.9	-9.8
CAMPHOR (1R)	35.0	19.0	24.0	-13.0	38.0	22.2	-8.8	14.0
LOPERAMIDE HYDROCHLORIDE	41.9	38.3	25.9	-9.0	33.7	27.6	1.3	-24.2
CEPHAPIRIN SODIUM	81.8	14.4	32.8	-3.7	94.7	78.6	41.8	-0.4
LINDANE	17.7	6.6	8.2	23.0	29.3	3.6	-21.5	3.3
CHLOROQUINE DIPHOSPHATE	25.1	-14.2	-0.2	-5.9	28.6	4.3	-33.8	-0.8
MESTRANOL	16.1	-5.9	4.9	0.4	20.3	5.2	-33.8	-1.9
RITODRINE HYDROCHLORIDE	20.5	-25.2	13.9	0.6	40.9	-0.6	-29.1	0.3
METOPROLOL TARTRATE	18.2	-4.8	8.2	-1.2	31.8	10.0	-19.7	2.4
FLURANDRENOLIDE	33.3	-29.0	10.4	9.7	43.9	-18.9	-14.4	0.1
NEOSTIGMINE BROMIDE	18.3	-8.0	9.0	-2.3	39.7	5.8	-4.5	0.2
KETOPROFEN	25.9	-16.6	7.6	5.9	41.4	-28.9	6.6	4.2
NORTRIPTYLINE	17.5	7.4	24.8	4.7	28.3	-8.0	-7.5	2.4
CEFOXITIN SODIUM	36.6	-26.2	12.4	7.1	61.7	2.2	-34.1	0.3
OXYTETRACYCLINE	99.1	96.4	101.1	65.4	82.9	77.7	22.7	-1.3
CETIRIZINE HYDROCHLORIDE	59.8	-15.9	22.2	-0.2	27.2	-22.7	-7.7	-2.3
PHENYLBUTAZONE	13.5	-7.3	18.6	13.0	3.8	-23.8	-13.8	1.9
METHSCOPOLAMINE BROMIDE	11.1	-24.3	8.0	8.4	-11.5	-54.2	-5.8	2.2
PROBUCOL	47.2	4.6	-1.5	-3.3	20.0	11.6	-14.2	0.9
DEBRISOQUIN SULFATE	14.6	-26.8	9.7	3.9	29.7	1.2	-20.0	9.4
NICARDIPINE HYDROCHLORIDE	47.3	10.2	0.0	-4.6	36.7	28.6	-4.7	-2.5
SULFAQUINOXALINE SODIUM	94.3	71.3	15.8	2.0	33.2	6.6	1.1	-1.0
CLEBOPRIDE MALEATE	54.0	20.2	12.7	-5.8	42.4	23.9	-11.8	7.2
MITOTANE	52.5	-5.4	-2.9	-9.5	31.5	3.6	-7.4	-2.3
ATROPINE OXIDE	44.6	6.7	-1.7	-4.3	40.5	17.8	5.9	-0.8
ACETYLCYSTEINE	31.5	-7.1	31.0	10.2	22.6	-6.6	31.3	15.0
PARAMETHADIONE	39.2	16.9	19.9	-7.3	28.1	10.0	27.3	4.2
AMITRIPTYLINE HYDROCHLORIDE	40.3	-19.2	3.6	-0.1	29.0	-0.1	7.2	0.5
SALICYLANILIDE	47.2	-4.1	6.9	-4.9	38.0	7.7	-13.5	7.0
ASPIRIN	26.0	-14.4	18.9	-7.4	25.3	1.3	17.3	-1.7
CLIOQUINOL	42.5	4.6	18.5	-3.6	19.9	-6.0	-15.6	4.7

BENZTHIAZIDE	8.3	-41.4	11.9	3.1	6.3	-38.1	14.0	0.5
PRAVASTATIN SODIUM	59.5	41.1	16.4	-1.2	18.8	0.5	13.0	31.2
CAPREOMYCIN SULFATE	54.0	7.4	1.6	-7.7	23.7	-0.6	-5.4	6.3
PODOFILOX	86.6	61.7	3.9	0.0	29.6	14.6	-19.4	-0.4
CEPHRADINE	65.4	19.5	15.2	-7.5	42.8	21.9	14.8	-0.8
ACRISORCIN	71.5	49.8	0.7	-5.9	35.7	22.0	12.7	1.1
CHLOROTHIAZIDE	60.6	16.2	9.8	-8.7	40.3	8.5	12.6	-0.1
CHLOROTRIANISENE	80.0	52.5	14.8	-6.6	45.5	22.9	5.8	-1.2
OXYQUINOLINE HEMISULFATE	44.0	0.9	23.5	-5.8	32.5	13.2	9.6	2.3
CLEMASTINE	64.5	30.7	14.2	-0.3	30.3	7.7	15.3	1.3
PIPERAZINE	51.7	3.8	-0.2	-8.1	34.8	4.7	-22.9	2.0
CORTISONE ACETATE	56.5	25.7	-5.2	-8.7	47.2	24.0	-25.5	-0.7
PROBENECID	44.8	-9.2	18.5	-8.4	27.5	6.8	-13.2	-2.1
DACARBAZINE	57.0	25.4	15.3	-7.2	42.6	19.2	1.3	-1.2
PYRANTEL PAMOATE	12.0	-31.0	11.3	-1.7	1.0	-11.4	2.8	-0.6
DEXAMETHASONE SODIUM PHOSPHATE	55.3	17.2	20.2	15.6	20.2	-6.2	-3.4	27.5
RESORCINOL	59.9	27.4	-0.1	-6.3	27.3	9.8	-21.2	-1.8
DIETHYLSTILBESTROL	68.6	39.9	30.0	0.6	19.5	19.1	0.6	4.0
STREPTOZOSIN	62.4	20.6	17.1	-8.6	38.0	20.8	2.3	-1.1
DIPHENYLPYRALINE HYDROCHLORIDE	67.2	34.0	9.6	-8.3	30.2	16.9	-7.4	-0.7
SULFATHIAZOLE	67.6	17.7	9.1	-9.2	48.2	19.8	-21.5	-3.5
DYPHYLLINE	67.0	32.3	-3.9	-9.5	43.0	15.1	-17.9	-2.2
THIMEROSAL	99.1	98.1	98.0	90.7	85.1	79.3	62.5	7.3
ESTRADIOL VALERATE	44.4	1.3	4.8	-8.6	24.6	-6.0	-1.6	4.9
TRIAMCINOLONE	40.9	0.3	11.4	-6.0	41.3	18.8	-13.6	-0.8
FLUDROCORTISONE ACETATE	49.3	8.7	3.6	-8.6	31.5	6.0	-6.9	-3.1
TRIPELENNAMINE CITRATE	42.2	-5.1	24.4	-9.2	45.0	18.2	-20.9	-2.7
GALLAMINE TRIETHIODIDE	35.4	-5.5	16.6	-8.5	31.9	2.3	-14.1	5.8
VIDARABINE	51.0	0.2	15.6	-4.5	28.6	-8.5	-5.3	-1.3
HALOPERIDOL	46.6	7.6	-0.5	-4.5	36.7	10.6	2.7	5.7
THEOBROMINE	65.2	27.4	3.2	-3.5	17.6	-0.2	-6.9	-1.1
HYDROCORTISONE HEMISUCCINATE	64.0	33.4	-3.6	-8.3	39.0	22.5	-5.7	-1.9
OUABAIN	67.6	39.4	13.6	-10.8	40.0	17.1	-7.9	-2.6
INDOMETHACIN	55.3	33.1	7.2	-8.8	38.0	23.9	-7.2	-1.2
CEFACLOR	85.3	57.0	20.9	-0.3	82.1	63.2	-1.2	-3.2
KANAMYCIN A SULFATE	63.1	27.0	2.1	-10.1	34.5	14.8	-1.5	-1.8
CANRENOIC ACID, POTASSIUM SALT	49.6	-6.8	15.1	-7.9	31.6	-0.4	13.4	1.8
METHICILLIN SODIUM	98.9	97.1	90.1	75.8	94.7	67.8	34.2	-0.9
FLUMEQUINE	95.2	82.6	58.6	43.5	64.2	46.0	-8.8	-1.6
CHLOROXYLENOL	46.7	4.4	10.0	-7.4	37.9	19.7	-22.8	-0.6

PENICILLIN G POTASSIUM	99.7	96.2	96.5	92.3	77.5	46.2	34.3	5.2
CLIDINIUM BROMIDE	39.6	-22.4	19.5	-6.4	23.5	13.4	0.0	5.6
PIROXICAM	30.9	-21.9	12.5	0.5	16.5	-20.0	-23.4	6.3
COTININE	56.8	19.1	-2.6	-9.2	13.1	-3.5	5.0	11.1
PROCAINAMIDE HYDROCHLORIDE	82.7	49.2	2.2	-8.4	26.4	8.0	-1.7	-2.2
DANAZOL	65.1	39.6	-19.0	-8.6	27.4	21.8	-14.5	-0.3
PYRAZINAMIDE	79.9	46.6	11.4	-7.7	47.8	20.0	-0.7	4.0
DEXTROMETHORPHAN HYDROBROMIDE	67.3	42.5	-3.6	-9.0	37.9	32.7	2.6	-0.8
RIFAMPIN	99.5	97.6	101.0	94.8	90.3	82.4	23.2	-2.2
DIFLUNISAL	66.0	35.2	1.7	-10.0	36.8	17.6	-5.1	-1.0
SULFABENZAMIDE	70.5	20.7	26.6	5.2	34.3	3.3	20.8	1.3
DIPYRIDAMOLE	45.1	-5.0	25.9	-8.4	33.7	22.4	16.1	1.1
SULFINPYRAZONE	68.2	14.6	19.1	1.3	35.8	2.6	-5.9	2.0
TRISODIUM ETHYLENEDIAMINE TETRACETATE	45.1	10.1	12.0	-7.5	30.4	18.0	-3.3	-4.1
THIOGUANINE	70.3	10.7	9.9	-8.7	34.7	-0.5	-3.3	-0.8
ESTRIOL	92.4	78.1	48.5	36.3	59.5	52.9	16.8	-0.4
TRIAMCINOLONE ACETONIDE	99.9	94.2	90.2	90.3	69.7	15.7	36.5	1.9
FLUMETHAZONE PIVALATE	59.6	26.8	7.4	-10.0	32.3	11.8	-11.0	-4.0
TRIPROLIDINE HYDROCHLORIDE	51.4	8.4	-7.1	-8.2	5.8	0.0	7.0	29.9
GEMFIBROZIL	74.9	34.9	0.2	-9.3	26.2	2.4	-23.7	-3.0
WARFARIN	60.1	28.0	-7.5	-9.3	24.9	11.9	-28.8	-2.4
HETACILLIN POTASSIUM	96.7	98.4	98.5	79.8	82.7	67.1	31.3	-2.4
STRYCHNINE	73.4	14.6	1.8	-10.6	40.3	12.0	-2.5	-1.7
HYDROCORTISONE PHOSPHATE TRIETHYLAMINE	62.8	20.6	12.5	-10.5	38.2	16.8	-6.9	-1.9
ALBUTEROL (+/-)	57.8	9.3	17.8	-7.8	38.5	13.8	16.7	-1.5
INDOPROFEN	39.9	-4.5	19.4	-6.6	25.4	14.3	7.6	3.9
IODIPAMIDE	51.4	-3.9	15.2	-7.0	44.0	32.1	4.0	-0.4
KETOCONAZOLE	80.1	34.5	14.3	-7.1	33.9	7.6	-19.7	0.3
CHENODIOL	40.0	-7.1	2.7	-7.1	28.9	7.2	-33.4	-2.1
METHOXSALEN	46.2	-1.4	18.4	-4.4	20.0	10.6	12.7	-1.6
FLUNARIZINE HYDROCHLORIDE	54.8	-34.7	26.4	2.0	16.8	-33.1	-20.2	-1.7
CHLORPHENIRAMINE (S) MALEATE	97.3	95.1	79.6	83.8	80.7	71.3	21.4	-1.1
PENICILLIN V POTASSIUM	99.5	97.1	97.7	88.5	83.7	65.2	41.0	-1.6
CLINDAMYCIN HYDROCHLORIDE	100.4	98.4	98.9	97.3	76.1	62.6	15.9	-2.0
POLYMYXIN B SULFATE	49.6	-27.2	7.4	-7.4	56.8	30.7	-9.9	1.1
CRESOL	73.2	38.4	-7.4	-9.9	15.9	25.0	31.2	-0.5
PROCAINE HYDROCHLORIDE	69.8	26.4	-7.1	-10.5	55.9	27.0	-22.3	-3.4
DAPSONE	66.1	37.7	2.1	-10.0	49.0	29.2	-26.3	-1.6

PYRILAMINE MALEATE	50.4	15.2	28.4	-5.3	28.2	2.8	27.1	1.5
DIBENZOTHIOPHENE	46.4	13.8	32.4	-7.4	34.6	16.2	17.7	4.0
ROXARSONE	42.2	3.8	10.8	-6.7	49.0	16.2	-2.5	0.1
DIGITOXIN	39.3	5.9	10.3	-7.4	46.2	23.7	-4.3	-1.5
SULFACETAMIDE	44.5	-10.9	34.2	-6.3	22.9	6.4	20.0	-3.4
PYRITHIONE ZINC	68.7	36.5	32.1	-6.4	62.5	31.4	-17.6	0.1
SULFISOXAZOLE	59.7	-18.1	35.6	2.1	14.3	-18.8	14.6	6.1
EMETINE	54.5	-14.4	1.0	-9.4	10.2	-12.8	10.9	20.4
THIORIDAZINE HYDROCHLORIDE	63.9	16.4	7.2	-8.4	19.1	16.5	-2.3	-2.4
ESTRONE	57.0	7.3	2.6	-9.1	25.6	10.3	-1.2	-1.1
TRIAMCINOLONE DIACETATE	52.6	11.8	15.1	-6.4	27.2	15.6	0.3	0.0
FLUOCINOLONE ACETONIDE	61.7	20.8	2.4	-9.8	28.5	25.2	4.8	-3.0
TROPICAMIDE	69.0	19.3	8.6	-10.6	39.3	14.2	-9.2	-1.3
GENTAMICIN SULFATE	59.1	12.7	14.0	-10.0	39.7	24.6	1.3	-5.4
XYLOMETAZOLINE HYDROCHLORIDE	49.9	7.6	26.6	-6.4	32.1	3.0	20.7	-3.4
HEXACHLOROPHENE	100.2	97.1	95.1	89.1	79.3	59.5	76.8	10.6
ACONITINE	47.5	-6.7	23.4	-3.6	31.9	12.0	0.9	0.9
HYDROFLUMETHIAZIDE	46.3	8.0	27.3	0.7	33.2	24.1	-5.2	-3.3
ARECOLINE HYDROBROMIDE	41.8	-7.8	20.0	-7.0	30.4	10.0	-13.1	-0.8
INOSITOL	36.0	7.8	24.5	-9.1	24.9	9.8	18.2	-1.1
LIOTHYRONINE	46.3	-13.0	10.5	3.7	14.0	-20.3	-6.6	2.9
LACTULOSE	65.1	16.5	0.4	-10.3	6.8	6.0	2.0	14.6
CHOLECALCIFEROL	71.8	35.2	-7.8	-10.9	15.9	4.0	-17.2	-1.0
METHYLERGONOVINE MALEATE	72.9	37.7	-2.6	-10.8	32.4	19.5	-69.4	-2.3
FLUPHENAZINE HYDROCHLORIDE	69.8	30.1	6.6	-11.0	31.2	18.7	-11.4	-2.9
CHLORPROMAZINE	63.3	37.9	8.5	-8.5	31.6	22.9	-3.9	-3.2
PHENOLPHTHALEIN	57.8	14.9	17.6	-9.4	41.2	15.4	-10.1	-2.7
CLOMIPHENE CITRATE	91.9	78.9	51.2	24.2	41.5	11.9	1.4	-4.7
PRAZIQUANTEL	55.1	-5.4	17.6	-4.3	30.5	1.4	4.9	1.2
CROMOLYN SODIUM	46.5	-11.8	22.1	-8.2	33.6	7.4	16.5	-3.2
PROCHLORPERAZINE EDISYLATE	48.6	4.9	22.6	5.1	32.8	21.0	-8.4	0.9
DAUNORUBICIN	72.5	32.9	16.6	-7.8	33.5	10.3	-4.8	0.2
PYRIMETHAMINE	42.3	-7.1	30.0	-5.7	31.9	10.4	-4.4	0.0
DIBUCAINE HYDROCHLORIDE	36.8	-4.2	20.8	12.6	32.1	17.7	5.1	0.6
SALICYL ALCOHOL	39.4	-37.9	21.6	-4.2	-1.7	-18.9	-2.8	-1.6
DIGOXIN	58.4	55.7	-16.1	-9.0	-2.4	30.3	-18.1	1.2
SULFADIAZINE	70.4	36.6	8.0	-9.9	41.0	26.3	-25.4	-2.7
DISOPYRAMIDE PHOSPHATE	89.7	71.1	-25.5	-9.2	31.1	19.4	-12.3	-4.1
SULINDAC	62.9	14.3	6.5	-8.7	37.7	16.2	-4.6	-3.0
ADRENALINE BITARTRATE	85.1	64.1	-3.5	-6.2	41.3	31.7	2.1	-4.0

THIOTHIXENE	62.7	3.7	14.0	-9.9	44.3	20.0	-10.0	-2.3
ETHACRYNIC ACID	76.9	50.4	-12.8	-11.0	56.1	33.7	-22.1	-2.2
TRIAMTERENE	53.8	7.4	23.2	-8.7	33.4	15.8	2.4	-0.6
FLUOCINONIDE	66.4	25.0	6.7	-9.1	32.6	13.2	7.4	2.3
TRYPTOPHAN	38.9	-3.5	11.1	-8.8	36.7	14.3	-5.7	2.1
GENTIAN VIOLET	71.4	100.0	100.5	92.2	63.1	59.6	-0.6	-3.1
ACETARSOL	39.0	-12.4	27.5	-6.1	31.6	10.4	1.2	-1.6
HEXYLRESORCINOL	37.8	0.7	29.3	4.7	34.1	14.7	-7.3	0.3
YOHIMBINE HYDROCHLORIDE	17.5	-33.9	17.1	-5.8	3.9	-15.0	0.7	4.6
HYDROXYPROGESTERONE CAPROATE	57.8	13.3	-2.2	-10.3	32.7	20.2	8.9	41.0
CAPTOPRIL	81.6	48.2	0.8	-8.7	29.9	12.7	-18.2	0.0
IODOQUINOL	56.9	30.4	10.0	-8.7	56.2	41.9	-8.8	0.0
ALLANTOIN	66.6	38.4	0.1	-9.4	33.7	17.3	4.0	-3.4
LEUCOVORIN CALCIUM	50.8	17.2	10.6	-9.0	47.1	38.5	-0.2	-1.5
CINCHONIDINE	56.3	23.3	9.8	-9.2	44.8	20.7	6.9	-2.4
METHYLTHIOURACIL	48.1	9.9	3.8	-10.1	68.9	54.7	-2.5	-1.8
FLUTAMIDE	48.1	7.9	24.7	-6.7	31.1	8.7	9.1	1.2
CHLORPROPAMIDE	35.9	-11.6	22.8	-2.2	54.2	34.1	8.7	0.0
PHENYLEPHRINE HYDROCHLORIDE	42.1	-12.8	16.4	-4.8	35.8	10.2	-2.2	-1.3
CLONIDINE HYDROCHLORIDE	43.0	-4.2	14.4	-7.4	50.7	30.7	-11.4	-2.9
PRAZOSIN HYDROCHLORIDE	38.5	-3.4	26.5	2.6	33.4	16.1	-13.1	-1.8
CYCLIZINE	33.3	0.9	26.2	9.2	53.6	39.7	-13.7	0.2
PROCYCLIDINE HYDROCHLORIDE	33.0	-33.6	19.0	-3.0	5.1	-11.0	-3.8	-1.7
SODIUM DEHYDROCHOLATE	55.1	22.7	2.3	-9.2	11.5	0.6	6.6	-0.5
PYRVINIUM PAMOATE	100.0	98.0	99.0	94.3	49.7	0.4	-64.3	-4.4
DICLOFENAC SODIUM	65.1	23.1	-13.1	-10.6	30.4	22.2	-31.3	-4.9
SALICYLAMIDE	47.6	-4.2	9.3	-8.6	28.8	15.1	5.7	-3.4
DIHYDROERGOTAMINE MESYLATE	54.5	29.5	5.1	-10.7	41.0	37.7	5.6	0.8
SULFAMERAZINE	51.3	10.4	8.8	-8.6	62.1	38.9	-12.3	-3.5
DISULFIRAM	54.3	29.0	5.2	-10.0	53.3	37.1	-0.7	-4.5
TAMOXIFEN CITRATE	100.7	97.7	83.2	79.3	45.4	18.7	11.3	-0.8
EQUILIN	44.1	-8.0	22.6	-4.7	35.4	20.3	-2.2	0.5
TIMOLOL MALEATE	35.6	-5.4	22.2	-5.3	24.0	13.8	-4.2	-1.3
ETHAMBUTOL HYDROCHLORIDE	43.4	1.8	25.2	-1.9	30.3	18.8	-2.7	0.2
TRICHLORMETHIAZIDE	27.7	6.1	29.4	-6.5	39.8	19.6	16.4	-1.2
FLUOROMETHOLONE	29.6	-1.7	22.1	0.9	22.6	14.0	5.6	-1.3
TUAMINOHEPTANE SULFATE	42.0	-9.6	21.8	-1.9	0.5	-7.1	-14.0	-2.3
GLUCOSAMINE HYDROCHLORIDE	52.7	7.6	4.4	-8.1	-0.8	16.1	-16.1	-2.3
MERBROMIN	77.2	43.6	1.6	-10.7	33.1	22.5	-30.0	-4.6
HISTAMINE DIHYDROCHLORIDE	58.6	24.0	11.9	-8.7	28.1	17.3	-11.6	-1.9

ADENOSINE PHOSPHATE	55.0	10.4	15.1	-9.4	25.2	11.2	11.5	-3.0
HYDROXYUREA	47.2	24.3	19.6	-9.1	34.7	31.0	16.2	-1.5
CIMETIDINE	49.1	14.6	20.5	-10.5	29.4	9.7	1.5	-2.1
IPRATROPIUM BROMIDE	56.0	27.0	17.7	-10.4	41.2	23.0	-13.9	-2.6
ALTHIAZIDE	47.7	-3.5	20.6	-3.6	34.0	10.5	10.6	-1.6
LEVONORDEFRIN	44.2	-0.3	33.3	0.7	36.6	21.5	10.6	4.8
CINCHONINE	26.3	-6.3	29.2	-7.4	27.1	12.4	-2.1	0.1
MICONAZOLE NITRATE	99.3	89.7	88.4	90.1	57.0	8.7	-10.2	-1.6
DROPERIDOL	35.7	-6.5	28.2	-4.1	38.7	15.8	-13.5	0.2
CHLORTETRACYCLINE HYDROCHLORIDE	98.9	97.7	95.7	89.1	78.9	67.7	39.5	-1.7
PHENYLPROPANOLAMINE HYDROCHLORIDE	36.7	-15.4	21.0	-5.2	-26.1	-5.8	20.2	-4.3
CLOTRIMAZOLE	97.3	86.8	46.3	61.3	39.9	-30.5	-27.3	-1.5
PREDNISOLONE	63.3	15.8	6.0	-10.1	21.3	6.3	-8.0	0.3
CYCLOPENTOLATE HYDROCHLORIDE	73.4	47.5	10.5	-11.1	27.3	21.7	-49.0	-2.2
PROMAZINE HYDROCHLORIDE	60.8	16.3	12.7	-4.3	34.1	10.4	6.4	-2.3
DEMECLOCYCLINE HYDROCHLORIDE	84.4	77.2	46.5	36.6	42.8	38.5	-17.2	-3.1
QUINACRINE HYDROCHLORIDE	51.9	6.3	7.4	-7.2	40.0	16.7	-3.4	-1.7
DICLOXACILLIN SODIUM	95.3	97.7	101.0	95.8	95.9	82.8	13.0	-3.5
SODIUM SALICYLATE	49.6	-1.7	25.5	0.0	35.9	11.5	9.0	-2.3
DIHYDROSTREPTOMYCIN SULFATE	74.0	34.0	21.9	-5.3	36.0	25.6	3.7	8.2
SULFAMETHAZINE	41.0	-20.4	13.8	-4.0	37.3	8.9	-3.6	-0.5
DOPAMINE HYDROCHLORIDE	58.7	-4.8	13.4	-7.7	38.1	19.4	2.2	0.1
TERBUTALINE HEMISULFATE	36.8	-18.0	27.3	-2.0	32.4	5.3	6.8	-2.1
ERGOCALCIFEROL	44.0	-14.9	24.2	-6.7	18.4	2.8	-1.9	-1.1
TOBRAMYCIN	15.4	-30.8	24.3	21.3	10.3	-14.1	-6.3	1.0
ETHINYL ESTRADIOL	45.9	4.0	13.1	-5.5	-10.4	29.2	-27.8	4.6
TRIFLUOPERAZINE HYDROCHLORIDE	79.9	33.1	-5.8	-8.4	33.7	16.8	-36.4	0.0
FLUOROURACIL	59.8	26.5	10.2	-10.0	25.1	17.3	-27.4	-4.4
TYROTHRICIN	62.6	10.9	2.3	-6.9	31.4	14.8	4.7	-1.3
GRAMICIDIN	51.5	-8.0	7.9	-10.0	41.5	34.5	1.1	-3.3
PHENACETIN	53.3	-20.9	4.9	-9.8	40.9	15.8	9.7	-3.9
HOMATROPINE BROMIDE	59.1	10.4	14.7	-10.1	42.1	30.7	0.2	-3.9
KETOTIFEN FUMARATE	33.6	-6.1	33.6	7.0	31.7	7.6	17.1	-0.6
HYDROXYZINE PAMOATE	59.3	13.1	16.2	-7.1	31.2	18.1	-0.5	-0.5
CLOZAPINE	29.5	0.6	15.0	-6.4	23.5	13.0	0.5	0.2
ISONIAZID	49.3	9.8	18.7	-7.9	31.1	13.7	3.8	-2.2
ADENINE	35.9	1.2	18.0	0.3	28.4	14.2	1.8	0.2
LINCOMYCIN HYDROCHLORIDE	98.9	98.3	92.1	89.3	76.2	73.1	29.4	-0.5
COENZYME B12	13.6	-13.0	26.3	5.8	-9.0	-10.8	3.3	6.4

NEOMYCIN SULFATE	47.1	5.6	-9.0	-11.4	8.3	16.0	-20.5	2.9
FAMOTIDINE	72.0	38.3	8.8	-12.6	21.7	10.9	10.3	3.1
CHLORTHALIDONE	97.1	97.6	98.4	86.4	81.0	71.6	13.2	-3.4
PHYSOSTIGMINE SALICYLATE	96.3	98.5	98.8	85.5	63.3	61.9	43.0	-2.4
CLOXACILLIN SODIUM	97.1	98.4	96.6	87.1	75.4	54.7	41.5	-0.2
PREDNISOLONE ACETATE	40.7	5.4	7.5	-7.8	38.3	16.2	-3.0	-0.3
CYCLOPHOSPHAMIDE HYDRATE	33.4	7.5	10.0	-6.4	37.7	25.4	-2.8	-1.7
PROPANTHELINE BROMIDE	49.5	2.9	31.7	-3.6	31.3	13.0	-19.6	-2.8
DESIPRAMINE HYDROCHLORIDE	35.0	2.3	33.2	6.9	27.0	15.5	13.8	0.6
QUINIDINE GLUCONATE	49.0	11.1	24.6	-5.6	32.3	15.4	-24.6	-1.7
DICUMAROL	66.8	44.4	44.0	6.1	41.5	26.2	13.6	-1.4
SISOMICIN SULFATE	32.9	-10.2	28.6	-6.0	23.0	3.0	19.3	-1.4
DIMENHYDRINATE	27.3	-10.3	19.0	5.6	23.6	14.0	-4.8	0.2
SULFAMETHIZOLE	27.2	-18.8	30.0	8.3	15.2	-17.6	-4.7	0.3
DOXEPIN HYDROCHLORIDE	36.8	6.1	10.6	-7.6	16.7	42.1	-8.8	-2.3
TETRACAINE HYDROCHLORIDE	73.1	41.0	1.3	-9.4	30.2	21.1	-10.7	-1.7
ERGONOVINE MALEATE	61.7	44.9	10.9	-8.0	30.7	31.2	-16.8	2.4
TOLAZOLINE HYDROCHLORIDE	45.9	-7.9	11.7	-8.9	43.1	32.1	4.1	-2.9
ETHIONAMIDE	42.6	10.0	17.9	-6.7	40.5	34.8	0.0	-0.5
TRIHEXYPHENIDYL HYDROCHLORIDE	54.5	9.0	12.0	-10.8	42.8	17.6	3.5	-2.3
FLURBIPROFEN	46.1	24.8	10.9	8.9	34.1	28.8	4.8	-2.1
UREA	46.8	7.6	22.1	-7.4	32.5	21.8	10.9	-0.1
GUAIFENESIN	41.7	5.2	21.8	-2.9	33.5	20.6	9.2	-1.1
PHENYLMERCURIC ACETATE	97.5	97.8	99.5	95.0	81.4	91.5	74.6	11.9
HOMATROPINE METHYLBROMIDE	38.7	-13.6	22.9	5.0	24.0	8.5	20.6	-0.2
BETAHISTINE HYDROCHLORIDE	29.3	-4.2	23.1	0.0	33.1	15.6	-3.9	-0.9
HYOSCYAMINE	29.6	1.0	28.3	8.2	18.3	14.7	12.8	1.5
HYDRASTINE (1R, 9S)	37.4	-19.6	14.3	-5.7	15.8	-7.5	9.5	14.3
ISOPROPAMIDE IODIDE	35.9	31.2	-2.0	-11.1	0.2	29.0	-15.4	-1.2
AMINACRINE	60.2	24.2	12.2	-9.2	17.9	16.1	-7.8	2.9
MEDROXYPROGESTERONE ACETATE	69.2	51.1	3.2	-8.2	22.2	19.9	-28.1	1.0
CHOLESTEROL	49.9	8.9	14.0	-10.2	27.2	13.5	7.5	-2.8
NITROFURAZONE	55.2	14.4	14.6	-9.4	40.3	37.3	6.2	-1.0
ETODOLAC	33.6	8.6	21.5	-8.0	53.8	36.7	-24.1	-1.6
CHLORZOXAZONE	34.4	-5.4	29.8	-7.9	37.8	29.5	-0.4	-4.9
PILOCARPINE NITRATE	33.0	7.7	27.0	4.1	43.5	26.3	1.3	-1.9
CLOXYQUIN	36.4	5.9	26.6	-7.1	28.0	24.6	4.7	-1.2
PREDNISONE	37.1	-13.7	22.1	-5.1	39.0	13.4	-8.8	-1.2
CYCLOSERINE	33.8	6.3	10.3	-2.8	27.9	20.6	-4.4	-1.2
DEXPROPRANOLOL HYDROCHLORIDE	39.5	-9.2	21.0	-7.6	38.8	13.7	-7.4	-2.2

DEXAMETHASONE	28.8	9.0	20.8	-5.6	24.5	20.2	5.7	-1.0
QUININE SULFATE	23.5	-23.9	33.7	0.4	7.4	-17.3	-18.2	0.8
DICYCLOMINE HYDROCHLORIDE	50.7	14.4	6.2	-7.7	12.6	35.2	-14.6	-0.3
SPECTINOMYCIN HYDROCHLORIDE	65.7	36.9	6.9	-10.3	12.6	14.7	-25.3	-0.7
DIMETHADIONE	48.1	26.6	11.9	-3.4	29.5	24.2	-21.4	-1.6
SULFAMETHOXAZOLE	64.9	19.1	23.7	-9.7	29.8	15.2	-4.2	-2.0
DOXYCYCLINE HYDROCHLORIDE	81.5	67.2	68.4	43.7	47.8	51.3	-6.2	-2.1
TETRACYCLINE HYDROCHLORIDE	75.9	54.2	59.8	22.2	66.3	57.1	-11.5	-1.9
ERYTHROMYCIN ETHYLSUCCINATE	96.1	97.2	99.5	91.5	91.3	87.0	6.3	-3.6
TOLBUTAMIDE	42.8	5.4	26.4	-8.9	29.6	13.3	10.7	-2.4
ETHOPROPAZINE HYDROCHLORIDE	44.9	25.0	33.9	-4.6	31.6	20.0	1.3	0.8
TRIMEPRAZINE TARTRATE	32.0	5.9	29.3	-9.9	48.6	19.5	-17.2	-3.5
FURAZOLIDONE	97.0	96.8	96.7	93.9	23.1	15.9	10.8	2.9
URSODIOL	22.8	-1.7	20.6	-8.1	40.4	12.8	-1.9	-2.7
GUANABENZ ACETATE	20.1	6.1	27.2	15.4	17.7	11.7	-4.4	1.7
SULFANILAMIDE	26.3	-19.8	16.9	4.5	-6.5	-15.1	-24.6	-2.0
HYDRALAZINE HYDROCHLORIDE	31.8	43.7	0.5	-8.6	-1.7	28.7	-15.7	-0.5
MOLSIDOMINE	53.9	15.0	8.7	-7.4	-16.1	33.9	-14.0	-0.2
IBUPROFEN	76.1	65.9	-7.5	-7.8	50.1	46.7	-17.3	-0.9
LIDOCAINE HYDROCHLORIDE	30.9	8.4	17.4	-8.0	-12.8	26.0	2.4	0.9
ISOPROTERENOL HYDROCHLORIDE	53.6	30.2	5.0	-7.7	56.3	54.9	12.0	-1.7
BEKANAMYCIN SULFATE	77.8	47.9	58.5	28.6	26.4	37.6	-12.3	-2.1
MEPENZOLATE BROMIDE	2.4	15.2	-2.8	-3.0	-17.3	-13.2	-37.2	12.5
PIPERINE	21.4	-9.3	-77.7	-3.6	12.7	-8.0	-166.1	-4.7
NITROMIDE	2.0	1.3	-15.7	-2.2	24.6	15.3	-40.6	-1.1
FENOTEROL HYDROBROMIDE	8.0	-22.3	4.6	1.2	18.9	-7.8	10.4	3.7
CICLOPIROX OLAMINE	8.3	7.1	-9.1	-3.2	19.0	2.0	-0.3	-0.4
PINDOLOL	42.5	-10.4	-5.9	-3.2	23.5	-2.5	-6.6	11.4
COLCHICINE	29.6	0.4	-7.0	-3.4	37.3	16.3	5.2	1.0
PRIMAQUINE DIPHOSPHATE	100.1	91.6	68.8	79.9	20.9	-27.9	22.0	12.2
CYPROTERONE ACETATE	-1.1	-22.3	19.7	6.4	21.0	-8.4	10.4	7.1
PROPYLTHIOURACIL	37.7	-20.9	-17.9	-2.5	32.8	-3.2	-28.4	0.3
DEXAMETHASONE ACETATE	-5.3	-14.3	-5.5	-1.7	21.8	8.4	-9.5	-0.3
RACEPHEDRINE HYDROCHLORIDE	29.9	-29.7	8.6	-3.8	19.6	-25.9	-24.4	11.8
DIENESTROL	-27.7	-16.5	-10.2	2.2	2.5	-0.9	6.3	-1.0
SPIRONOLACTONE	38.4	-31.0	-10.8	-1.6	-0.3	-50.7	-12.6	42.6
DIOXYBENZONE	-8.0	5.7	-7.7	-0.6	-6.6	-16.6	2.2	30.6
SULFAPYRIDINE	24.6	3.9	-9.8	-0.6	46.8	21.1	-36.6	2.7
DOXYLAMINE SUCCINATE	51.6	39.5	-12.5	5.6	42.3	27.1	-24.7	0.2
TETRAHYDROZOLINE HYDROCHLORIDE	25.6	6.0	-3.2	-2.8	43.4	9.2	-2.2	6.3

ESTRADIOL	27.0	17.8	-0.4	0.7	32.4	16.0	-6.2	8.3
TRANYLCYPROMINE SULFATE	40.0	2.6	-9.8	-4.6	56.5	23.1	-2.9	4.0
EUCATROPINE HYDROCHLORIDE	48.7	27.0	1.8	-3.6	37.5	13.9	-12.7	1.2
TRIMETHOPRIM	98.0	96.1	85.2	84.8	57.8	34.3	6.8	0.6
FUROSEMIDE	40.7	-7.4	11.5	3.3	27.6	7.0	22.4	7.9
VALPROATE SODIUM	39.5	5.5	5.4	-2.3	46.0	15.1	-15.5	0.0
GUANETHIDINE SULFATE	23.6	11.4	-1.1	-2.4	43.5	22.6	0.7	0.7
AZELAIC ACID	36.4	-15.0	7.5	-3.0	33.7	11.5	11.6	-2.9
HYDROCHLOROTHIAZIDE	19.4	-8.2	8.2	-2.2	30.7	15.1	-13.0	2.8
MYCOPHENOLIC ACID	0.5	-32.0	5.8	4.9	14.7	-22.7	4.3	33.6
IMIPRAMINE HYDROCHLORIDE	21.6	5.1	0.7	-3.6	9.6	-3.1	0.0	46.1
PHENTOLAMINE HYDROCHLORIDE	39.9	25.5	-2.9	-3.0	31.0	9.1	-15.4	-1.8
ISOSORBIDE DINITRATE	52.1	44.0	-9.4	-3.0	57.5	43.5	-21.3	0.0
BUDESONIDE	37.0	24.3	-5.9	-3.4	30.1	3.9	1.4	-1.2
MERCAPTOPURINE	54.7	34.3	-8.5	-2.6	33.7	12.7	-3.2	1.9
ETOPOSIDE	80.1	53.1	13.0	-5.0	47.1	20.0	-5.4	-0.2
NORETHINDRONE ACETATE	58.2	29.2	-15.3	-5.1	40.4	6.0	-27.3	0.2
FENBUFEN	42.7	0.1	0.1	-4.4	19.6	-3.7	27.4	3.5
CINOXACIN	51.0	16.2	6.2	-4.9	25.7	-9.5	29.5	3.1
PIPERACILLIN SODIUM	61.7	39.9	37.9	-4.7	71.1	45.2	-7.5	2.6
COLISTIMETHATE SODIUM	44.3	16.1	2.0	-4.5	35.7	20.4	-8.8	1.8
PRIMIDONE	44.8	12.4	6.4	-4.5	40.7	19.1	11.4	-2.1
CYTARABINE	33.9	3.2	12.1	-2.1	34.2	11.2	4.5	7.0
PSEUDOEPHEDRINE HYDROCHLORIDE	4.8	-24.9	15.3	-0.3	26.1	-3.7	9.2	2.9
DEFEROXAMINE MESYLATE	34.7	11.1	-12.8	-4.4	-3.4	-9.5	7.0	23.1
RESERPINE	59.5	38.1	-7.2	-4.5	34.3	8.7	-24.8	-3.2
DIETHYLCARBAMAZINE CITRATE	65.1	49.9	-6.0	-3.9	53.4	36.0	-21.7	0.2
STREPTOMYCIN SULFATE	27.1	13.2	-0.7	-4.4	39.1	5.0	-0.7	-1.7
DIPHENHYDRAMINE HYDROCHLORIDE	46.2	22.3	1.9	-5.0	34.3	15.9	19.2	-2.1
SULFASALAZINE	50.5	31.8	-7.5	-6.2	39.5	4.0	-3.2	-1.6
DYCLONINE HYDROCHLORIDE	71.9	46.4	-7.1	-5.7	45.2	19.3	10.3	-1.2
THIABENDAZOLE	46.6	10.7	18.1	-2.8	27.6	-3.2	14.7	4.4
ESTRADIOL CYPIONATE	39.0	15.4	17.4	-4.4	36.3	10.2	10.5	3.7
TRIACETIN	40.1	2.0	8.4	-3.0	38.2	17.1	-13.5	0.3
EUGENOL	42.8	13.3	4.8	-4.5	41.6	26.3	-1.6	0.7
TRIOXSALEN	39.4	6.9	14.7	-4.6	45.1	7.2	7.2	-1.8
FUSIDIC ACID	96.8	96.2	94.7	91.6	91.0	79.0	-0.7	-1.6
VANCOMYCIN HYDROCHLORIDE	96.2	92.3	95.3	95.9	21.7	-9.7	-13.9	19.3
HALAZONE	23.7	12.9	8.3	-2.6	10.3	-11.1	-2.6	19.7
PHENETHICILLIN POTASSIUM	98.8	98.4	100.1	84.9	77.3	43.0	30.1	-1.7

HYDROCORTISONE ACETATE	56.0	23.6	-3.7	-3.7	66.1	37.9	-23.0	1.3
OLEANDOMYCIN PHOSPHATE	75.8	53.8	22.1	-4.7	43.5	13.3	-3.9	-2.6
INDAPAMIDE	37.1	16.8	4.4	-0.9	46.1	16.9	-7.8	-0.4
BUTAMBEN	53.8	34.1	4.6	-6.6	45.3	7.1	-11.1	-0.6
ISOXSUPRINE HYDROCHLORIDE	44.9	24.4	-0.8	-3.8	54.3	28.3	-21.3	-0.2
BRUCINE	26.3	10.7	27.8	-3.7	31.8	2.2	21.6	3.1
METHENAMINE	37.1	25.9	8.4	0.8	29.8	0.1	33.0	7.7
DEHYDROCHOLIC ACID	35.1	0.4	14.5	-2.0	39.4	11.0	1.1	1.0
OXIDOPAMINE HYDROCHLORIDE	50.7	18.1	1.4	-3.3	70.6	48.5	-26.5	-0.4
MEBEVERINE HYDROCHLORIDE	55.7	-20.9	17.1	-3.2	33.1	20.6	18.9	-0.9
ACECLIDINE	21.4	-18.5	19.3	-1.6	44.4	18.2	-13.6	2.6
BUPROPION	8.6	-50.0	26.1	-1.8	13.7	-6.0	14.0	13.4
MESNA	6.6	14.0	10.8	-2.1	10.2	-13.1	-4.9	8.4
ROFECOXIB	47.9	6.1	-2.0	-4.3	61.4	27.7	-25.8	-2.8
CARBOPLATIN	52.4	41.4	-0.3	-3.5	72.5	51.7	-49.7	-1.0
ORLISTAT	39.8	11.6	7.4	-3.8	49.9	17.0	-0.4	0.5
CYCLOSPORINE	38.9	32.4	9.1	-3.0	43.7	17.0	-0.5	1.0
ESTROPIPATE	59.8	39.3	4.2	-6.5	61.9	32.9	-11.1	-1.0
BIOTIN	50.2	26.2	1.6	-4.4	51.5	29.2	-14.4	-0.2
EZETIMIBE	38.0	8.0	12.3	-1.8	25.8	-7.3	15.6	2.7
AMIPRILOSE	30.3	21.6	28.0	0.1	34.1	17.0	8.1	3.2
CILOSTAZOL	46.3	17.2	7.6	-3.2	47.4	12.2	-7.6	-0.1
TRAZODONE HYDROCHLORIDE	58.1	29.6	15.6	-3.6	39.6	20.5	-0.7	-2.6
CHLOROPHYLLIDE Cu COMPLEX Na SALT	46.7	11.9	1.8	-3.7	45.5	0.3	-29.6	-0.4
DOBUTAMINE HYDROCHLORIDE	15.6	-7.5	19.0	-2.0	39.5	21.5	8.8	4.3
KETANSERIN TARTRATE	12.2	-50.8	26.1	-0.9	33.2	-0.7	-4.3	4.2
HYCANTHONE	40.2	26.4	-1.9	-5.4	34.4	3.7	-3.1	-1.1
BISMUTH SUBSALICYLATE	26.7	14.5	5.5	-6.9	4.5	-6.5	-8.9	1.3
AMSACRINE	47.1	20.9	-6.8	-6.4	44.0	16.6	-23.9	-3.3
D-LACTITOL MONOHYDRATE	56.0	45.8	14.7	-5.7	41.4	20.5	-21.9	-2.6
ROXITHROMYCIN	97.7	98.4	99.0	94.4	74.0	67.9	-8.4	-2.4
SULFISOXAZOLE ACETYL	56.2	25.5	0.7	-5.5	48.7	15.7	-21.1	-1.0
OXIBENDAZOLE	67.2	51.8	-8.3	-4.8	42.3	20.1	-14.3	-2.1
NETILMICIN SULFATE	50.8	22.2	24.0	-4.2	33.4	2.5	12.9	2.8
NIMODIPINE	56.4	32.0	19.9	-4.3	30.3	5.0	14.8	1.8
NORGESTIMATE	29.7	6.1	-19.9	-5.4	33.7	8.8	-27.0	-1.2
TERFENADINE	44.5	8.8	13.2	-2.2	39.5	14.9	-21.6	0.1
AMINOPENTAMIDE	60.9	23.4	12.5	-3.2	42.0	17.1	2.7	1.0
PACLITAXEL	64.0	41.4	-2.2	-5.0	41.8	25.9	-11.4	1.4
ROLIPRAM	41.0	-18.4	19.3	-2.4	19.3	-19.7	-15.7	2.9

TACROLIMUS	27.6	12.5	4.3	-5.8	-0.6	-2.1	-22.5	-0.4
EPIRUBICIN HYDROCHLORIDE	82.3	64.3	11.9	-5.7	65.4	33.9	-40.7	-3.9
CAPSAICIN	65.7	52.0	-5.0	-4.4	49.9	26.9	-11.6	-2.0
CEFUROXIME AXETIL	60.1	-1.7	18.5	-3.4	78.4	53.3	-1.6	-1.8
TRETINON	52.0	15.1	7.5	-4.5	38.0	16.7	-13.1	-2.8
SIMVASTATIN	58.5	44.3	-1.0	-6.2	56.1	22.5	-22.8	-2.0
CISPLATIN	55.1	32.9	12.3	-3.7	47.4	22.0	-26.1	-0.7
MOXIFLOXACIN HYDROCHLORIDE	99.1	97.7	89.6	84.1	89.6	54.9	36.5	1.0
ASCORBIC ACID	44.2	7.1	25.8	-2.8	39.7	15.7	10.1	0.9
CLAVULANATE LITHIUM	68.7	26.9	34.8	-1.8	81.9	58.1	-1.8	0.4
AKLOMIDE	46.7	11.2	15.1	-4.3	29.9	17.9	-11.7	-1.6
OLMESARTAN MEDOXOMIL	44.5	17.2	16.5	-5.3	50.7	11.2	-17.2	-0.8
TIAPRIDE HYDROCHLORIDE	25.8	-0.8	17.4	-2.3	33.0	6.1	-24.8	0.6
CITICOLINE	29.0	-22.3	27.2	-3.9	23.7	-18.5	-0.2	7.5
MENTHOL(-)	12.4	5.8	2.8	-4.3	1.0	-25.4	-3.6	9.8
BIFONAZOLE	92.1	65.9	21.6	-5.4	59.4	0.9	-67.5	-4.4
EDOXUDINE	57.9	52.0	-2.5	-5.5	35.4	18.6	-32.7	-3.9
FIPRONIL	56.7	31.8	0.6	-3.5	29.1	1.4	-18.8	-1.1
PYRIDOSTIGMINE BROMIDE	37.4	23.3	12.2	-5.3	34.0	15.2	-5.0	-2.0
BENZOYLPAS	58.5	36.8	1.0	-4.6	40.4	13.6	-15.0	-1.9
MIDODRINE HYDROCHLORIDE	55.6	34.9	6.7	-7.6	39.0	17.7	2.6	-4.7
LEVOCARNITINE	30.3	-22.1	19.7	-4.6	40.0	6.4	13.4	-3.2
MITOXANTHRONE HYDROCHLORIDE	28.3	34.4	30.5	-2.4	43.8	17.6	11.7	-2.3
TAURINE	22.1	0.2	15.5	-2.6	29.6	-3.4	-19.0	1.3
PIPOBROMAN	47.7	23.1	18.8	-6.0	39.0	17.3	-15.8	-0.4
DOXORUBICIN	59.6	23.2	16.9	-2.8	39.0	6.8	-14.8	1.0
ACYCLOVIR	33.4	3.4	23.3	-5.2	41.2	21.9	-19.1	-0.6
TERAZOSIN HYDROCHLORIDE	-2.0	-27.0	30.1	-0.8	21.0	-14.3	5.1	11.8
CLOPIDOGREL SULFATE	37.9	23.8	3.4	-5.0	14.1	-5.6	-9.0	1.8
ARSANILIC ACID	53.5	26.8	11.6	-4.8	45.0	17.6	-19.0	-3.2
BUTACAINE	67.7	60.4	-8.8	-5.1	54.5	33.3	-16.8	-1.0
ROLITETRACYCLINE	44.5	25.7	15.8	-6.3	39.8	9.8	-0.3	-3.4
BROMPHENIRAMINE MALEATE	55.1	42.8	13.5	-3.2	41.7	18.3	-11.6	-1.5
VECURONIUM BROMIDE	57.3	34.8	5.3	-4.8	44.8	20.1	-8.4	-3.5
FAMPRIDINE	51.7	32.0	10.0	-7.1	45.0	23.5	-7.3	-3.0
FEXOFENADINE HYDROCHLORIDE	33.0	8.0	20.1	-3.0	31.4	2.3	14.4	3.9
BRETYLIUM TOSYLATE	19.5	8.4	24.4	-4.5	30.2	9.8	16.1	-0.6
OXCARBAZEPINE	19.0	-11.5	12.8	-1.1	35.0	14.5	-8.5	1.5
ZIDOVUDINE [AZT]	41.3	15.9	16.7	-2.6	37.0	21.7	-9.1	0.2
PIOGLITAZONE HYDROCHLORIDE	55.6	16.6	18.1	-2.0	37.8	18.0	-28.9	-2.2

MENADIONE	28.4	0.8	22.2	-2.2	32.5	15.2	-5.6	22.6
ALCLOMETAZONE DIPROPIONATE	29.5	-11.7	30.4	0.0	5.2	-5.5	-5.5	3.5
NICOTINYL ALCOHOL TARTRATE	-3.7	15.2	22.5	-2.7	-5.2	-6.9	-2.1	9.6
CEFTIBUTEN	98.0	98.7	100.0	93.1	68.3	48.4	11.6	-4.8
BACAMPICILLIN HYDROCHLORIDE	98.2	99.2	99.9	98.0	93.2	73.8	-4.0	-3.8
APRAMYCIN	33.6	15.0	18.2	-3.5	41.5	10.7	1.1	-1.1
THONZYLAMINE HYDROCHLORIDE	41.1	28.8	12.3	-3.9	55.6	35.1	3.2	-4.7
TYLOSIN TARTRATE	96.7	98.7	98.8	95.6	83.5	76.2	-10.7	-4.1
ENOXACIN	95.6	96.9	78.9	48.2	63.9	47.9	-13.5	-2.8
DECOQUINATE	22.4	-2.5	20.6	-3.7	49.8	14.8	11.1	0.7
ISOXICAM	31.0	8.2	29.2	-4.0	32.6	11.4	0.1	0.1
BROMINDIONE	46.3	19.8	11.1	-2.4	39.4	15.4	-9.3	0.1
NADOLOL	28.1	14.4	3.8	-2.6	33.8	16.8	-4.0	1.1
LOBENDAZOLE	38.5	1.3	18.5	-2.7	39.2	13.9	2.7	-2.3
OXETHAZAINE	37.6	-1.0	28.3	-2.5	39.4	19.0	-11.2	0.4
THIAMINE	30.1	-22.9	20.7	-1.7	18.6	-16.3	-1.1	17.7
ETANIDAZOLE	8.3	7.2	3.2	-4.0	9.3	-15.6	11.2	9.4
METHYSERGIDE MALEATE	66.6	21.0	9.5	-5.5	62.9	34.5	-24.2	-4.2
RETINYL PALMITATE	97.9	100.2	99.7	95.1	52.5	37.5	-11.7	-1.6
TIOCONAZOLE	98.9	94.7	95.4	76.6	66.9	11.4	-37.9	-2.8
LORATADINE	53.6	34.4	2.8	-4.2	35.7	2.9	2.4	-2.3
PANTHENOL	43.6	0.1	5.6	-4.4	59.2	27.3	-2.3	1.7
CLOBETASOL PROPIONATE	29.5	8.6	7.0	-3.6	45.4	19.3	-0.8	-1.7
PIPAMPERONE	17.9	5.9	22.3	-2.6	35.7	9.9	13.1	6.0
SIROLIMUS	37.5	22.1	27.6	-2.4	28.7	6.2	15.9	28.8
ALISKIREN HEMIFUMARATE	45.4	10.1	17.4	-1.9	38.4	11.0	4.0	14.8
NICERGOLINE	26.3	7.9	7.7	-3.8	42.8	20.3	-8.6	7.9
TRIFLURIDINE	86.8	47.1	27.8	-1.7	39.6	-1.9	17.9	10.4
FOSCARNET SODIUM	40.9	7.1	23.0	-1.8	28.2	10.8	17.3	8.9
MELOXICAM SODIUM	37.3	-18.7	15.6	-3.1	16.3	-12.0	16.5	31.5
AZACITIDINE	30.5	10.7	6.0	-5.3	-3.1	-4.7	-6.6	-0.4
DONEPEZIL HYDROCHLORIDE	33.6	-14.1	9.6	-5.4	46.1	15.3	-16.3	-2.4
SALICIN	40.8	23.6	13.7	-3.7	64.0	33.4	-15.1	-4.1
ALENDRONATE SODIUM	85.3	59.6	21.8	-5.1	51.1	29.7	-4.9	-1.0
FLOXURIDINE	99.7	99.0	99.6	85.8	86.3	65.3	0.5	-2.8
CEFDINIR	99.1	99.0	100.9	97.6	93.0	74.2	33.8	4.4
BENDROFLUMETHIAZIDE	57.9	22.0	7.2	-6.5	55.8	26.6	-5.7	-2.8
SERTRALINE HYDROCHLORIDE	30.4	11.3	30.1	-4.4	22.6	15.9	17.7	0.2
THIAMPHENICOL	52.3	41.5	25.3	-3.7	38.9	12.4	3.4	-0.2
SARAFLOXACIN HYDROCHLORIDE	99.7	97.2	92.7	83.8	85.2	69.8	25.9	0.2

ETHISTERONE	23.0	-10.5	9.1	-0.4	35.4	2.9	-7.8	1.1
CEFDITORIN PIVOXIL	97.2	79.5	90.7	76.1	77.2	48.0	47.1	11.9
LABETALOL HYDROCHLORIDE	27.1	-9.0	26.8	-2.0	36.7	20.5	-7.9	0.8
CAPOBENIC ACID	34.7	-16.8	24.0	0.9	9.9	-12.4	10.6	5.7
NALTREXONE HYDROCHLORIDE	5.9	6.2	13.8	-3.1	5.5	-6.0	-2.9	0.3
METHYLENE BLUE	43.4	35.9	14.4	-8.4	56.3	16.2	-25.6	-6.1
DIPYRONE	66.2	64.0	8.4	-2.9	73.4	53.1	-18.2	-3.3
TRICLOSAN	100.4	98.7	100.9	93.9	76.4	43.5	40.1	4.3
NAFRONYL OXALATE	50.2	38.8	9.9	-3.7	58.1	29.6	-6.0	-2.1
SOLIFENACIN SUCCINATE	67.4	28.1	20.7	-5.9	33.9	6.4	-15.9	9.5
THALIDOMIDE	43.5	20.9	12.8	-4.8	49.1	24.4	-19.2	-2.5
ERGOTAMINE TARTRATE	25.1	9.6	20.3	-2.2	28.8	8.1	10.5	1.2
SELAMECTIN	40.1	18.6	25.0	-4.6	35.4	11.5	-1.5	-0.5
PHENTERMINE	49.1	-14.6	14.5	-4.3	34.7	14.5	0.0	8.7
IOPANIC ACID	42.8	40.4	-21.0	-1.5	31.8	4.7	-49.1	1.5
PANCURONIUM BROMIDE	43.5	-31.3	20.0	-2.5	32.5	9.0	1.0	7.9
PAROXETINE HYDROCHLORIDE	43.9	11.1	24.5	-2.2	30.8	8.6	-9.5	7.1
ACAMPROSATE CALCIUM	31.4	-19.5	20.5	-2.3	16.2	1.8	2.9	18.9
SPIPERONE	27.5	13.9	5.4	-4.5	5.3	-8.4	1.2	11.2
PIRENPERONE	51.4	40.9	10.3	-2.3	43.7	19.7	-17.2	-1.7
CEFSULODIN SODIUM	42.3	35.6	10.7	-4.5	76.5	60.8	-13.2	-4.3
CARVEDILOL	36.7	14.5	12.9	-4.1	38.2	8.1	-11.1	-2.9
CYCLOHEXIMIDE	49.7	40.3	7.9	-3.4	44.4	23.5	-4.0	-2.7
FLUVASTATIN	54.3	29.2	15.8	-3.9	47.6	12.8	-3.9	-0.8
MONENSIN SODIUM	61.2	52.2	19.8	-3.9	69.5	47.6	2.2	-1.1
ACARBOSE	29.2	8.9	27.4	-4.0	28.6	10.0	21.2	3.3
ALTRETAMINE	25.3	15.6	22.1	-1.4	30.5	11.4	18.9	1.7
SIBUTRAMINE HYDROCHLORIDE	19.8	5.6	18.2	-2.8	37.4	18.0	-8.6	2.5
BEPRIDIL HYDROCHLORIDE	23.6	10.2	26.9	-1.6	34.3	25.4	-3.9	1.1
ALFLUZOSIN	43.9	9.5	23.1	-2.7	34.3	17.3	13.4	0.3
TENOXICAM	26.5	8.0	22.6	-4.0	38.6	20.5	-19.5	2.4
CLOPIDOL	3.2	-18.1	20.0	-1.1	-8.2	-6.3	3.1	1.7
PARAROSANILINE PAMOATE	-9.8	-9.4	8.4	-1.8	10.3	-3.6	-7.8	2.5
VALACYCLOVIR HYDROCHLORIDE	46.1	32.2	1.3	-3.3	50.7	19.7	-12.9	-1.8
LEVAMISOLE HYDROCHLORIDE	43.4	52.5	16.7	-5.7	66.8	60.5	-10.4	-4.1
ACETOHEXAMIDE	58.4	47.0	8.3	-2.8	57.0	28.6	-17.7	-1.2
CYCLOTHIAZIDE	53.6	50.1	16.2	-4.1	47.7	31.2	-8.5	-0.6
METHYLATROPINE NITRATE	50.4	34.6	6.7	-5.7	52.1	25.0	-12.3	-2.3
SULFANILATE ZINC	28.8	20.7	15.4	-3.5	51.2	31.3	-15.7	-2.6
TRIMETHADIONE	47.3	10.7	20.5	-1.9	26.5	11.2	10.9	0.5

QUIPAZINE MALEATE	42.7	27.6	23.7	-4.1	36.3	14.0	10.9	-0.7
ACEPROMAZINE MALEATE	21.4	2.5	14.2	-1.7	37.3	15.4	-7.7	7.9
NITRENDIPINE	2.2	8.5	22.8	6.5	29.9	19.6	-10.1	0.2
ANAGRELIDE HYDROCHLORIDE	62.0	36.4	12.4	-2.6	29.2	19.5	-3.4	1.3
NAPROXOL	49.8	28.1	21.6	-3.6	35.0	13.8	-16.6	8.8
TRIENTINE HYDROCHLORIDE	49.8	-2.0	11.7	-2.1	7.1	-13.8	-0.4	27.1
KETOROLAC TROMETHAMINE	89.7	100.1	90.3	76.3	-19.1	25.4	20.3	-1.1
FUMAZENIL	49.0	37.0	16.0	-6.4	66.3	40.2	-13.8	-4.4
ETHYLNOREPINEPHRINE HYDROCHLORIDE	42.2	44.3	14.2	-4.4	62.2	46.4	-30.5	-2.1
PREDNISOLONE SODIUM PHOSPHATE	66.4	48.8	-5.1	-4.4	38.5	10.5	-14.6	-1.6
ERYTHROMYCIN ESTOLATE	76.7	69.4	18.6	-7.0	34.3	31.8	-11.9	-0.2
AVOBENZONE	72.7	52.5	5.5	-4.0	58.7	30.9	6.4	-1.1
FOSFOMYCIN CALCIUM	47.1	33.6	8.7	-3.0	48.0	32.9	-8.6	-1.4
IRBESARTAN	27.1	12.6	22.7	-3.2	36.0	8.0	6.5	-1.6
TINIDAZOLE	51.5	28.5	21.4	-1.7	36.0	13.8	-0.3	2.5
PIZOTYLINE MALATE	26.0	10.2	29.6	-3.0	43.5	16.2	-9.3	-0.2
ABAMECTIN	22.9	20.3	9.0	-1.8	21.9	17.6	-13.0	0.9
ROPINIROLE	39.7	5.9	25.0	-1.8	33.7	19.0	-3.7	-0.6
AMINOHIPPURIC ACID	54.7	36.0	11.8	-3.0	31.0	17.2	-8.8	1.8
PERINDOPRIL ERBUMINE	19.0	-14.3	28.0	4.1	10.7	-16.3	-3.0	5.4
BROMHEXINE HYDROCHLORIDE	42.0	28.1	5.1	-2.9	4.8	-7.8	-14.0	17.0
TELITHROMYCIN	98.6	99.0	100.2	88.0	94.5	83.5	1.2	-2.0
CHLOROXINE	79.5	46.1	28.9	-1.9	60.1	43.9	-9.9	-2.4
CHLORMADINONE ACETATE	50.7	34.6	10.1	-4.5	38.2	10.9	-8.4	-3.1
PERHEXILINE MALEATE	100.0	98.4	86.0	30.8	33.8	25.4	-12.0	0.5
DULOXETINE HYDROCHLORIDE	50.2	40.0	3.9	-4.1	41.2	17.0	-5.7	0.0
MEPHENTERMINE SULFATE	42.6	29.0	21.5	1.6	38.7	24.4	-5.8	-1.6
ETHOXZOLAMIDE	40.8	28.6	16.8	-1.5	32.3	9.4	7.0	0.8
NICLOSAMIDE	100.1	96.2	86.7	81.4	82.4	47.2	61.3	9.1
NITHIAMIDE	71.2	32.2	18.1	-1.9	33.7	11.8	-3.5	1.7
URETHANE	31.2	12.7	22.9	-2.0	31.7	20.6	9.5	5.5
ZINC UNDECYLENATE	49.2	24.8	18.1	-2.9	35.1	15.4	-10.3	1.5
RITANSERIN	29.0	8.9	29.0	1.0	25.2	11.7	7.5	2.4
BIPERIDEN	33.6	7.1	17.5	-4.2	-23.8	-7.3	-1.5	1.3
BENZALKONIUM CHLORIDE	98.5	99.0	66.1	59.9	4.6	-37.7	15.6	2.4
ETOMIDATE	59.7	31.7	3.5	-4.4	56.6	23.9	-34.4	-1.9
COLFORSIN	63.8	59.3	15.8	-0.3	33.8	26.2	-26.5	2.4
TICLOPIDINE HYDROCHLORIDE	67.6	32.4	-4.2	-4.0	46.3	11.3	-8.3	-1.2
LANSOPRAZOLE	46.8	40.4	9.0	-4.1	27.2	22.0	0.7	8.0
ALTRENOGEST	34.2	19.7	9.0	-2.2	47.2	12.4	-12.4	0.4

ALAPROCLATE	32.6	19.3	10.4	-5.9	32.2	21.2	-17.6	6.4
PREGNENOLONE SUCCINATE	55.9	23.7	30.1	-3.4	27.7	-9.5	5.9	4.7
ESTRADIOL PROPIONATE	6.2	-8.3	18.4	-0.1	22.0	0.5	0.7	4.0
ATOVAQUONE	40.7	-0.2	5.5	-3.7	41.3	11.4	2.0	0.2
CEFAMANDOLE NAFATE	27.6	11.5	19.8	-0.4	32.7	25.6	-23.1	0.5
LEVOFLOXACIN	99.0	95.8	97.3	89.3	80.3	58.7	33.4	-1.2
CARBIDOPA	42.9	-7.0	26.0	-3.9	35.3	9.4	-29.6	1.0
EXEMESTANE	22.8	-23.2	17.3	-1.0	-19.7	-7.7	1.2	22.1
BENZOIC ACID	17.3	10.8	9.5	-1.8	1.4	-9.2	-11.2	4.0
QUETIAPINE	11.1	2.5	21.4	-2.7	36.7	20.3	-35.6	-2.4
MEFLOQUINE	51.9	48.1	15.8	-3.2	60.4	51.7	-29.4	-1.7
ROSUVASTATIN CALCIUM	24.7	8.8	22.7	-3.9	29.7	6.8	-2.4	-1.9
CARMUSTINE	40.9	38.7	11.8	-2.9	36.6	28.7	0.0	-1.6
OXAPROZIN	29.3	14.4	23.0	3.7	34.7	10.7	-4.6	0.0
CHLORPROTHIXENE HYDROCHLORIDE	54.9	36.6	8.2	-3.3	46.3	31.5	-15.0	-2.0
OXICONAZOLE NITRATE	98.7	92.9	76.5	82.9	41.0	-28.6	-8.7	0.3
PAROMOMYCIN SULFATE	12.5	-21.9	30.3	4.7	23.3	7.0	0.8	1.1
NISOLDIPINE	9.8	-12.4	26.4	4.4	37.5	15.3	-24.3	-2.6
METARAMINOL BITARTRATE	20.6	13.6	32.1	5.8	26.7	20.6	-11.5	-2.0
FLUCYTOSINE	26.8	-19.3	21.1	3.4	38.0	21.5	-10.7	-2.1
NOMIFENSINE MALEATE	15.0	-4.8	24.3	7.8	14.4	19.6	11.9	-1.3
PRALIDOXIME CHLORIDE	17.8	-16.3	23.7	5.7	-8.5	-12.3	-6.1	2.7
THIRAM	-31.6	5.2	28.2	4.9	-12.1	4.3	-11.4	1.4
UNDECYLENIC ACID	8.8	2.1	20.6	22.6	22.5	43.6	-48.1	-2.3
SEMUSTINE	28.6	32.4	16.2	-1.8	32.3	34.1	-35.9	-0.1
DEXCHLORPHENIRAMINE MALEATE	11.4	20.0	16.6	-1.7	34.6	7.3	-1.3	-2.2
CIPROFLOXACIN	99.9	99.1	97.2	92.6	83.2	66.1	10.4	-1.5
LAMOTRIGINE	33.9	17.5	17.6	-3.3	39.4	14.8	-5.9	-1.1
ISOSORBIDE MONONITRATE	11.5	14.2	-1.9	0.8	36.7	13.8	-53.3	1.1
TICARCILLIN DISODIUM	102.8	99.9	101.3	100.3	91.4	57.5	20.6	9.8
MEXILETINE HYDROCHLORIDE	56.9	21.8	9.2	-1.0	59.8	25.4	-40.5	-2.4
BISOPROLOL FUMARATE	10.9	-15.6	5.5	7.7	41.8	-15.5	-33.4	8.9
ACETRIAZOIC ACID	50.5	20.5	5.2	0.2	41.5	14.4	-26.3	-1.8
DARIFENACIN HYDROBROMIDE	9.6	-9.6	11.5	2.3	41.1	-15.7	-22.9	6.5
ESTRADIOL BENZOATE	18.2	7.1	-0.1	6.5	29.3	2.8	-21.1	1.6
TRIMETOZINE	5.0	-23.0	20.1	22.7	38.3	-26.0	-12.2	1.8
LIOTHYRONINE (L- isomer) SODIUM	25.1	40.8	-37.9	-0.7	11.8	6.7	-62.2	-2.3
LITHIUM CITRATE	22.7	-24.2	18.2	19.9	15.2	-27.0	-87.6	3.8
ETHOSUXIMIDE	1.0	-8.8	12.1	6.7	24.9	-23.2	-32.0	-2.3
TILMICOSIN	103.5	98.8	101.4	100.5	30.6	3.1	7.7	-4.4

BENZYL BENZOATE	8.2	-13.1	-1.6	9.5	26.4	-13.1	-18.7	-3.2
RIZATRIPTAN BENZOATE	41.9	-23.7	15.2	34.6	-7.3	-45.9	-39.7	-0.1
ADIPHENINE HYDROCHLORIDE	-27.4	12.6	12.1	4.2	4.1	5.7	-38.2	-1.6
RAMIPRIL	52.4	16.6	5.0	3.8	44.7	-1.1	-33.1	-0.6
CEFTRIAXONE SODIUM TRIHYDRATE	72.4	56.3	8.8	-0.1	65.4	38.7	-44.3	-2.3
OXFENDAZOLE	47.7	31.8	22.5	0.4	38.2	4.8	3.7	2.1
CINNARAZINE	54.8	31.3	3.4	2.0	44.1	15.2	-7.1	-0.6
AZAPERONE	6.6	0.5	17.6	0.2	50.0	11.9	-25.2	-2.6
METHAPYRILENE HYDROCHLORIDE	39.6	19.4	7.3	3.8	68.1	34.9	-36.7	-2.4
MONTELUKAST SODIUM	-44.7	4.1	4.9	1.2	27.7	-14.8	-9.1	0.1
METHAZOLAMIDE	25.6	16.8	11.7	0.3	57.4	23.2	-20.6	-1.0
FOMEPIZOLE HYDROCHLORIDE	24.2	2.9	10.5	0.0	41.8	-3.3	-16.8	-0.9
PERGOLIDE MESYLATE	40.6	20.8	13.0	12.3	38.9	1.9	-27.7	-1.1
PREDNISOLONE HEMISUCCINATE	7.3	-24.5	19.7	0.3	26.0	-8.3	-6.4	-1.5
THIOTEPA	39.9	-2.3	20.8	1.4	20.4	-3.1	-24.5	0.5
CLINDAMYCIN PALMITATE HYDROCHLORIDE	-33.2	-29.1	13.7	4.0	7.2	-28.0	4.9	-1.0
SPIRAMYCIN	63.7	57.8	28.1	-0.7	29.0	19.2	-22.3	-1.2
DILOXANIDE FUROATE	39.6	24.7	5.4	-0.7	73.3	40.8	-49.4	0.1
CELECOXIB	33.5	44.1	2.7	0.4	59.0	28.9	-41.9	-1.3
RALOXIFENE HYDROCHLORIDE	47.4	30.6	37.0	-0.2	61.4	30.5	-28.8	0.5
AMCINONIDE	40.9	23.5	23.0	1.1	40.4	14.5	-26.6	1.1
TETRAMIZOLE HYDROCHLORIDE	45.0	19.4	6.4	-1.2	69.8	36.2	-39.8	0.4
MORANTEL CITRATE	23.7	30.9	7.2	0.2	61.3	34.0	-14.0	1.9
FLUDARABINE PHOSPHATE	41.8	17.6	15.8	-0.5	64.5	17.1	-17.5	-1.7
VENLAFAXINE	23.6	25.5	11.6	0.3	50.9	21.6	-14.7	0.7
DESOXYMETASONE	29.9	7.6	1.7	-0.4	51.6	10.4	-32.3	-1.6
RETINOL	35.2	29.2	10.9	0.6	33.4	5.1	-41.6	-0.3
ZOXAZOLAMINE	-15.0	2.9	-3.4	-2.1	45.6	8.6	-61.6	-1.3
ALRESTATIN	-14.2	-10.1	21.2	4.5	19.7	-3.8	-22.6	1.7
GATIFLOXACIN	94.9	95.3	92.8	98.6	70.1	3.3	41.5	-0.7
PIPERIDOLATE HYDROCHLORIDE	43.2	26.5	1.5	22.7	7.4	-0.8	-10.5	-1.1
FLUNIXIN MEGLUMINE	73.9	60.8	1.5	0.8	58.4	22.7	-42.0	-2.6
BENZOYL PEROXIDE	76.5	63.5	-2.6	-1.0	48.3	23.8	-27.3	-2.8
FAMCICLOVIR	60.2	39.2	17.7	-1.1	47.0	10.6	-12.4	-2.2
QUINAPRIL HYDROCHLORIDE	71.2	54.8	5.2	-2.2	30.6	5.7	6.8	-1.9
ESCITALOPRAM OXALATE	76.0	48.2	3.3	-0.9	62.4	21.1	-10.0	-3.3
TRIMIPRAMINE MALEATE	68.3	49.9	24.8	-1.4	47.9	13.1	-21.8	-2.4
AMITRAZ	67.3	40.8	8.8	-0.2	54.2	13.5	-14.0	-0.2
DANTROLENE SODIUM	23.6	10.3	28.0	1.4	57.3	24.0	-18.9	-1.9

TRANILAST	71.2	45.6	13.6	2.5	50.8	7.2	-27.5	-0.8
BETA-PROPIOLACTONE	47.4	26.6	7.6	0.7	44.9	5.2	-22.0	-2.3
BENURESTAT	51.0	15.9	32.3	0.2	50.2	4.8	-8.4	-1.7
METHYLBENZETHONIUM CHLORIDE	100.7	97.3	99.0	90.7	37.4	0.6	-45.7	-1.7
GLIPIZIDE	39.0	-21.5	4.5	23.3	13.1	-20.5	4.3	-0.8
PRILOCAINE HYDROCHLORIDE	47.7	22.1	10.1	1.9	6.4	-3.0	-25.6	-1.5
PYRIDOXINE	74.8	56.2	5.7	8.1	62.9	30.5	-48.4	-3.7
TETROQUINONE	71.6	62.4	4.8	-1.0	73.5	47.9	-37.8	-2.9
CEFONICID SODIUM	88.2	66.3	38.5	9.8	67.5	47.2	-5.8	-3.0
CLOFIBRATE	69.0	50.1	23.0	-1.8	60.6	26.2	-28.4	-1.0
ETIDRONATE DISODIUM	78.1	50.0	9.5	0.2	53.4	18.9	-16.3	-2.7
AZITHROMYCIN	98.8	97.9	99.5	96.6	85.5	75.4	-35.3	-2.8
CEFPODOXIME PROXETIL	81.9	58.4	41.5	2.9	84.9	54.8	37.8	-1.5
BUPIVACAINE HYDROCHLORIDE	59.9	38.1	6.1	-1.6	73.2	39.3	-14.4	-0.1
TOLTRAZURIL	63.1	43.6	11.2	3.0	40.6	4.0	-23.6	-1.1
PERPHENAZINE	68.5	55.7	21.3	0.7	47.6	9.5	-19.1	0.0
MUPIROCIN	101.8	98.1	100.5	97.1	81.2	80.6	20.4	-1.6
CITALOPRAM	44.2	3.8	16.0	-0.2	54.8	11.1	-30.5	1.5
BETAMETHASONE ACETATE	-5.4	-1.2	17.4	16.8	13.4	-29.4	-6.4	1.9
ISOTRETINON	24.4	31.1	14.3	-1.1	10.0	-5.9	-21.9	-2.5
CYSTEAMINE HYDROCHLORIDE	68.1	52.8	8.8	-3.7	53.9	29.2	-41.6	-2.8
PROADIFEN HYDROCHLORIDE	74.1	66.6	5.2	-1.7	62.7	38.6	-37.6	-1.8
MIGLITOL	70.9	55.2	26.7	-0.8	53.3	17.1	-2.4	-0.5
ANISINDIONE	75.6	62.2	20.9	-1.1	46.8	21.7	0.9	-0.5
CLORSULON	65.0	25.6	16.1	-2.8	53.2	17.1	-11.5	-1.1
BETAINE HYDROCHLORIDE	74.1	34.1	19.0	-1.2	57.4	27.0	-15.3	-0.2
AMLODIPINE BESYLATE	19.8	17.7	32.1	-2.3	62.1	18.6	-3.4	0.8
AMIFOSTINE	67.2	42.4	16.8	0.8	48.4	21.1	0.7	-0.6
DERACOXIB	57.3	35.1	22.8	-1.1	42.0	1.8	-6.9	-0.5
TRIFLUPROMAZINE HYDROCHLORIDE	73.4	50.7	9.8	-0.8	44.9	15.9	-7.9	0.5
PEFLOXACINE MESYLATE	101.6	97.1	99.3	94.7	85.1	65.1	28.0	-0.2
BETAMETHASONE 17,21-DIPROPIONATE	30.7	-16.9	11.0	1.0	34.5	-6.5	-10.9	0.1
AZELASTINE HYDROCHLORIDE	47.0	-14.1	24.5	14.2	24.7	-29.4	-14.1	10.4
HALCINONIDE	58.1	36.8	15.2	-1.7	36.1	8.9	-25.4	-2.9
BENZOXIQUINE	-7.5	1.9	14.3	-0.6	-3.4	5.8	-26.2	-3.7
METHYLPREDNISOLONE SODIUM SUCCINATE	61.4	45.3	9.9	-2.3	35.1	8.4	-39.9	-3.1
GUANFACINE	45.3	41.5	11.9	-1.4	53.2	28.0	-36.0	-1.6
HYDROCORTISONE BUTYRATE	44.7	38.3	27.1	-1.9	57.6	26.7	-0.9	-2.2
RIMANTADINE HYDROCHLORIDE	66.2	37.2	5.4	-1.6	48.3	14.7	-13.5	-1.4

SULFANITRAN	75.6	49.4	-0.6	-1.0	50.1	21.3	-23.1	-2.1
IFOSFAMIDE	32.7	10.4	12.6	-1.7	47.3	5.5	-4.6	-0.5
RESORCINOL MONOACETATE	67.8	47.6	14.1	-1.5	53.1	23.6	-5.2	0.1
NATAMYCIN	61.7	25.7	14.6	-0.4	48.6	14.1	-7.1	-1.2
ANETHOLE	59.7	43.3	0.0	1.5	51.7	15.9	-30.5	1.3
TADALAFIL	43.3	-9.9	12.6	0.2	45.6	4.9	-9.0	-0.9
ALBENDAZOLE	55.0	33.0	12.0	1.4	34.2	5.6	-36.8	-0.2
TOREMIPHENE CITRATE	73.2	42.5	66.4	57.3	13.8	-25.8	-23.0	2.6
RIBAVIRIN	14.5	13.9	11.3	-1.9	-1.3	-7.6	-23.5	-1.4
TEICOPLANIN [A(2-1) shown]	98.5	98.3	99.1	94.0	85.4	65.3	-1.7	-3.5
FLUOXETINE	70.0	42.9	-0.8	-0.6	59.6	30.8	-35.0	-3.2
ERYTHROSINE SODIUM	53.6	45.3	-47.8	-1.2	61.7	35.8	-64.3	0.1
ISOFLUPREDNONE ACETATE	59.7	46.2	24.3	-1.6	51.6	23.7	-14.9	-1.9
BROXYQUINOLINE	83.8	53.4	55.3	22.1	81.5	28.7	-21.2	-3.1
GLYCOPYRROLATE	75.8	54.8	6.7	1.2	55.4	19.0	-20.6	-3.3
URSOLIC ACID	28.1	10.9	11.9	-0.1	64.4	24.3	-118.5	0.4
METHYLPHENIDATE HYDROCHLORIDE	65.2	35.8	16.2	-0.7	68.5	37.7	-8.9	-1.0
TELENZEPINE HYDROCHLORIDE	44.4	19.6	20.6	0.4	60.6	18.2	-30.7	1.6
CLOFAZIMINE	64.2	40.5	3.9	-0.3	38.9	3.8	-20.1	-1.6
OMEGA-3-ACID ESTERS (EPA shown)	-0.3	-19.7	8.0	2.8	71.1	27.6	-22.5	-2.2
DENATONIUM BENZOATE	36.5	12.4	4.3	1.0	44.5	5.6	-37.5	1.6
BENFLUOREX HYDROCHLORIDE	55.0	-6.3	24.1	0.3	17.1	-25.7	-16.0	-0.1
ALPRENOLOL	17.7	9.5	19.3	-1.2	-8.8	-12.5	-17.8	-0.2
LUFENURON	56.3	18.7	6.2	-2.4	43.7	15.7	-53.7	-3.0
PENTYLENETETRAZOL	74.0	58.4	17.3	-1.4	62.6	38.5	-36.3	-2.6
CLOFOCTOL	80.1	58.4	28.5	-2.1	67.0	38.7	1.9	0.0
DEXPANTHENOL	68.7	56.4	28.9	-3.7	63.1	39.4	-2.5	-1.4
CHLORALOSE	68.2	27.9	9.8	-1.8	65.2	32.4	-12.1	-0.3
CLEMIZOLE HYDROCHLORIDE	75.5	54.7	22.9	-1.8	65.3	35.0	-23.7	-4.2
CHINIOFON	54.1	35.8	17.9	-1.1	56.5	12.8	1.0	-3.3
CLOFIBRIC ACID	43.9	23.7	19.0	-2.8	70.9	38.0	-8.4	-2.5
CLENBUTEROL HYDROCHLORIDE	66.1	34.4	21.4	2.3	60.5	10.0	-29.3	-1.0
ERYTHROMYCIN STEARATE	102.4	97.6	96.9	93.4	97.1	82.4	-15.6	-0.5
PRASTERONE ACETATE	41.9	0.3	14.8	2.6	37.6	-2.4	-20.4	-1.3
AMBROXOL HYDROCHLORIDE	-44.8	6.3	12.7	3.2	56.2	25.2	-18.1	0.0
QUININE ETHYL CARBONATE	56.2	1.3	18.3	-0.8	7.0	-21.9	6.2	2.1
OLEANOLIC ACID ACETATE	1.6	13.5	9.4	0.8	6.5	15.0	-52.3	-3.8
PIROMIDIC ACID	79.0	57.8	6.3	-3.0	54.9	26.5	-23.3	-1.0
NIPECOTIC ACID	61.6	51.9	14.9	-1.6	54.0	30.6	-20.2	-1.3
5alpha-CHOLESTANOL	67.9	49.5	21.4	-4.4	69.8	34.8	-8.4	-3.7

ESTRONE BENZOATE	60.7	42.9	27.9	-3.0	52.6	26.9	-19.3	-3.1
SORBITOL	58.3	34.8	16.8	-2.5	64.3	32.8	-15.4	-2.4
TRICHLORMETHINE	49.4	23.3	18.8	-1.8	56.4	35.3	-18.4	-3.2
MEGLUMINE	43.1	28.6	25.0	-2.6	71.8	27.3	-6.2	-0.2
BETAMETHAZONE SODIUM PHOSPHATE	19.9	7.4	30.1	-0.7	53.4	33.0	-5.2	-3.6
ALFAXALONE	55.2	11.9	23.2	2.4	49.0	8.4	-13.4	0.5
ITRACONAZOLE	29.6	-2.8	27.2	2.4	56.9	23.4	-19.4	-0.6
METAMECONINE	4.6	-3.8	24.8	-1.3	60.5	17.3	-7.2	-0.6
RABEPRAZOLE SODIUM	13.5	4.6	13.6	4.8	35.6	15.5	-13.4	-2.0
6-HYDROXYTROPINONE	42.5	-4.7	24.1	7.9	23.1	-14.0	-14.3	13.2
BENZYDAMINE HYDROCHLORIDE	-11.5	20.2	12.8	0.1	-2.3	-3.5	-13.6	-1.7
DICHLOROPHENE	58.3	49.1	47.9	1.6	71.5	41.3	-22.8	-1.7
MESALAMINE	70.4	60.3	-0.1	-1.4	49.6	31.4	-11.8	-4.3
6,2'-DIMETHOXYFLAVONE	56.2	36.5	8.7	-2.3	47.7	-14.2	-25.3	-2.1
AMIODARONE HYDROCHLORIDE	61.7	55.9	24.7	-2.6	47.7	28.6	9.7	-0.9
NIMESULIDE	29.3	24.6	7.2	-4.7	56.0	19.6	-10.7	-3.6
NICOTINE DITARTRATE	73.8	55.1	21.9	-3.3	54.9	32.2	-16.4	-3.7
XYLOSE	29.2	20.3	20.9	-1.8	45.8	4.7	-5.3	0.6
NONOXYNOL-9	55.3	29.8	25.4	-0.5	26.0	9.1	2.8	-2.1
CYSTINE	24.2	4.5	19.7	-0.4	52.6	9.4	-6.8	-0.9
HEXESTROL	62.5	43.1	18.4	1.7	24.8	9.7	-5.2	-2.7
PIRIBEDIL HYDROCHLORIDE	40.7	9.9	14.8	1.5	47.4	6.5	-21.4	-1.3
AVOCADENOFURAN	27.2	19.2	25.8	1.2	27.3	6.0	-14.5	0.6
CYPERMETHRIN	33.6	-14.5	16.2	1.2	3.7	-30.9	-6.7	1.5
SODIUM TETRADECYL SULFATE	21.2	31.6	3.5	-1.3	-5.9	-7.4	-9.1	-0.6
HYDROXYTOLUIC ACID	65.9	38.2	12.6	-3.5	24.8	-9.6	0.2	-1.7
CAPSANTHIN	53.4	44.7	11.8	0.4	44.6	28.0	-25.8	-1.2
CETRIMONIUM BROMIDE	101.7	98.4	94.5	89.9	54.0	25.6	-9.2	0.1
DEHYDROVARIABILIN	24.6	4.1	17.5	-2.1	33.1	18.1	-22.9	-1.0
AMRINONE	72.6	43.5	19.2	-2.8	51.7	16.3	-13.1	-0.7
1-(2-METHOXYPHENYL)PIERAZINE HYDROCHLORIDE	70.1	50.0	10.1	-3.1	42.1	24.8	-7.5	-1.7
OLSELTAMIVIR PHOSPHATE	58.2	33.6	20.9	-1.5	42.7	0.5	2.3	1.7
5-AMINOPENTANOIC ACID HYDROCHLORIDE	43.8	27.2	20.5	-1.5	32.0	20.2	4.1	1.0
BROMOPRIDE	69.9	43.9	19.0	0.2	48.8	8.3	-24.7	-0.9
3,6-DIMETHOXYFLAVONE	57.3	23.5	8.9	1.1	14.5	-3.1	4.1	-2.7
EFAROXAN HYDROCHLORIDE	47.4	7.8	21.3	1.8	47.3	5.6	-16.4	0.4
MELENGESTROL ACETATE	-4.8	-1.8	23.8	1.2	37.1	0.9	-11.4	1.0
DROFENINE HYDROCHLORIDE	55.3	13.3	11.2	1.1	14.2	-26.7	-2.0	7.7

OCTISALATE	5.7	0.7	23.8	-1.5	14.9	6.0	-25.9	-1.3
PYRITHYLDIONE	40.9	17.1	7.5	-0.9	45.5	16.6	-16.7	-2.9
RISEDRONATE SODIUM HYDRATE	51.3	44.4	10.6	-1.6	59.8	32.6	-21.9	-4.6
DICHLORISONE ACETATE	41.9	-2.7	30.7	0.8	40.9	22.4	9.5	-1.8
DOXAZOSIN MESYLATE	36.6	30.2	20.1	-3.8	57.0	35.2	-17.2	-2.6
CHICAGO SKY BLUE	72.1	64.8	19.5	-2.8	52.6	31.4	-4.1	-2.2
ETHAMIVAN	68.2	49.9	12.3	-1.4	68.1	40.3	-11.9	-3.6
FORMESTANE	49.1	26.3	17.1	-1.7	40.1	12.4	-16.7	0.4
BUSPIRONE HYDROCHLORIDE	53.9	36.7	20.9	-3.6	48.1	28.1	-11.5	-2.7
IDEBENONE	59.9	40.6	10.4	3.3	39.3	17.6	-22.4	-1.9
TACRINE HYDROCHLORIDE	55.9	41.0	27.4	1.6	46.9	15.2	-17.7	0.6
TRICLABENDAZOLE	31.1	14.0	34.4	0.1	30.9	6.2	2.6	0.7
DOCOSANOL	-34.1	3.1	15.5	1.4	34.4	10.3	-10.2	-0.9
UVAOL	48.9	12.4	2.9	5.4	16.6	-6.3	-127.6	-0.5
FTAXILIDE	-77.1	23.5	18.5	-0.7	-0.8	3.2	-16.4	-2.1
NIFLUMIC ACID	60.8	52.4	4.7	-3.0	56.7	30.3	-28.8	-3.2
DIBUTYL PHTHALATE	78.4	70.9	18.1	-0.2	59.7	41.5	-41.3	-1.5
MOXISYLYTE HYDROCHORIDE	47.3	14.3	30.8	0.2	44.9	13.7	10.9	-3.3
SILIBININ	67.4	55.8	15.0	0.1	41.0	21.8	-0.2	-1.4
XYLOCARPUS A	67.0	31.6	30.1	-1.3	45.0	20.8	-21.9	-2.9
3-HYDROXYTYRAMINE	72.8	55.0	11.0	-2.7	36.6	15.1	-15.7	-2.5
EUPHOL ACETATE	31.7	8.3	28.2	-0.7	58.0	20.8	-11.2	-2.2
4'-METHOXYCHALCONE	60.4	24.5	6.8	-0.5	21.1	-9.8	-8.8	3.0
L-DEOXYALLIIN	53.9	25.9	19.9	-0.6	62.2	17.2	-9.6	-0.2
4-HYDROXYANTIPYRINE	48.9	26.7	25.0	3.1	44.3	23.5	-15.6	0.1
MANNITOL	16.4	-15.7	17.7	-2.5	40.8	6.9	-5.3	0.3
ACETYLTRYPTOPHANAMIDE	17.7	-7.8	28.0	0.9	28.4	7.6	-1.2	0.7
L-BUTHIONINE SULFOXIMINE	49.3	1.5	5.5	3.2	7.2	-0.4	-4.8	3.4
3,4'-DIMETHOXYFLAVONE	-33.6	-5.0	11.4	-1.5	19.2	11.2	-99.0	-2.9
CHROMOCARB	62.7	44.7	10.9	0.3	43.7	9.0	-28.5	-2.9
PHTHALYLSULFACETAMIDE	42.4	39.5	13.0	-3.4	53.7	37.4	-32.3	-2.5
ETHAVERINE HYDROCHLORIDE	40.6	25.6	26.1	-0.4	48.6	3.5	-5.1	-2.7
RIBOFLAVIN 5-PHOSPHATE SODIUM	28.5	24.7	30.6	-1.0	58.9	38.4	-26.7	-2.0
IRIGENOL	54.5	29.8	14.7	-0.2	44.9	10.7	-43.0	-2.4
SUCRALOSE	36.2	13.5	27.4	-2.7	61.6	43.8	-22.9	-1.4
NIFUROXAZIDE	88.0	78.1	61.6	44.5	39.3	-0.2	-5.0	-1.8
ISOETHARINE MESYLATE	5.6	-15.2	28.7	2.1	64.5	38.2	-3.9	0.2
MECYSTEINE HYDROCHLORIDE	49.6	31.4	27.8	0.6	23.9	-2.0	-21.9	-0.3
AZTREONAM	10.6	1.9	26.7	1.3	60.4	36.2	-37.8	-0.6
BAMBUTEROL HYDROCHLORIDE	25.5	2.6	17.5	2.8	47.3	8.7	-14.6	-2.0

LOXAPINE SUCCINATE	0.9	-9.5	30.6	1.0	36.8	16.0	-15.5	-1.7
OXYPHENONIUM BROMIDE	45.8	5.3	20.8	2.7	7.4	-16.6	-8.3	1.8
DIMERCAPROL	-24.5	-4.3	25.5	1.8	1.7	-10.8	-30.9	-1.7
METAMPICILLIN SODIUM	39.0	33.0	15.9	-0.6	43.8	19.3	-45.0	-1.6
OCTODRINE	67.6	61.3	15.3	-0.1	51.1	39.3	-26.3	-5.2
CLORGILINE HYDROCHLORIDE	100.6	98.2	99.0	86.7	59.4	25.7	-5.0	-1.0
ACEXAMIC ACID	27.2	31.2	24.1	-1.3	56.5	32.4	-17.4	-1.4
METICRANE	68.6	41.4	32.1	-2.1	50.4	25.0	-20.0	-4.2
TIMONACIC	31.6	17.6	23.2	0.0	34.9	25.0	-7.5	-4.2
BARBITAL	30.9	-5.8	28.6	2.1	45.0	7.8	-11.2	-1.5
HEXETIDINE	25.6	8.9	32.7	-0.8	47.7	23.8	-12.1	-2.3
DOXIFLURIDINE	97.4	86.8	80.1	53.9	84.5	59.6	-1.8	-2.0
CELASTROL	100.1	97.2	100.4	96.1	54.3	10.1	-4.3	-2.2
2,4-DINITROPHENOL	14.2	-3.8	17.8	1.7	40.9	0.1	-16.2	-0.2
2-ETHOXYCARBONYL-2-ETHOXYOXALOYLOXYDIHYDROCHRYSIN DIMETHYL ETHER	10.5	-3.4	22.4	0.0	24.4	-3.9	-24.3	-0.4
SOTALOL HYDROCHLORIDE	15.2	-12.7	18.7	6.1	13.5	-4.9	-7.0	0.6
IPRIFLAVONE	-71.3	-5.1	24.2	-1.9	8.5	1.3	-5.7	-1.4
THEAFLAVIN MONOGALLATES	89.8	79.1	40.8	-2.5	61.8	37.7	-19.3	-5.7
ACETYLTRYPTOPHAN	44.7	40.2	19.6	0.4	59.0	33.1	-36.1	-3.7
ALEXIDINE HYDROCHLORIDE	64.7	39.3	30.2	-1.9	42.8	8.0	-13.8	-1.9
FURALTADONE	73.7	55.2	26.7	-1.7	49.5	34.4	-19.6	-2.4
PIPEMIDIC ACID	33.2	11.4	23.4	0.4	38.7	12.7	-10.6	-1.4
TRICHLORFON	49.7	26.5	31.8	0.3	62.8	42.9	-16.3	-2.6
FENDILINE HYDROCHLORIDE	43.2	8.5	34.0	0.5	30.3	3.2	-12.8	-2.5
SELEGILINE HYDROCHLORIDE	15.1	19.1	27.2	1.5	44.5	23.5	-11.5	0.8
PASINIAZID	22.2	-21.4	29.6	2.8	28.6	-10.2	-11.8	-1.9
COLISTIN SULFATE	42.6	-18.0	27.3	2.1	46.0	14.3	-33.2	-1.7
2-METHYLENE-5-(2,5-DIOXOTETRAHYDROFURAN-3-YL)-6-OXO--10,10-DIMETHYLBICYCLO[7: 2: 0]UNDECANE	4.5	-11.5	27.2	0.5	27.2	3.5	-17.9	-2.2
FLORFENICOL	99.9	96.2	97.1	84.7	77.8	68.5	-13.4	-2.5
BUFLOMEDIL HYDROCHLORIDE	17.1	-8.0	22.2	1.3	9.4	-22.2	-1.5	2.0
TYLOXAPOL	-6.4	9.3	25.6	1.6	-8.4	-1.6	-29.5	-2.9
DROPROPIZINE	31.1	37.2	21.7	-1.3	56.4	25.9	-33.8	-1.5
DIAZOXIDE	54.1	44.6	18.0	-0.4	56.7	38.1	-54.0	-0.7
PEMPIDINE TARTRATE	13.2	26.1	26.9	-0.9	40.2	22.7	-3.9	-2.9
METOLAZONE	11.3	26.4	29.0	-1.8	56.8	33.8	-6.5	-1.0
ETICLOPRIDE HYDROCHLORIDE	42.6	24.8	38.9	0.7	40.4	1.4	-19.5	-1.2

ANIRACETAM	69.1	50.7	34.7	0.7	54.4	29.3	-10.4	-1.7
EPIESTRIOL	15.9	18.1	27.4	-0.2	46.5	10.2	-13.4	-1.6
DIPYROCETYL	68.1	49.2	23.8	0.4	44.9	25.6	-11.8	-0.5
PROTOVERATRINE B	28.4	22.5	34.2	2.4	33.6	8.5	-2.1	-0.2
METHYL ORSELLINATE	55.5	29.8	23.1	0.8	49.6	17.3	-25.9	0.0
OXEDRINE	-17.2	-19.8	25.9	3.0	38.2	13.5	-19.1	-2.6
FIPEXIDE HYDROCHLORIDE	-17.3	-22.5	22.5	1.2	29.5	5.9	-22.6	1.1
EFLOXATE	-14.4	-11.5	20.1	9.5	8.4	-17.1	-35.5	0.1
DIALLYL SULFIDE	-40.8	1.0	26.4	-1.5	-7.0	5.9	-9.7	-2.5
TARTARIC ACID	39.0	25.5	20.0	0.0	47.7	18.6	-40.4	-3.5
IDAZOXAN HYDROCHLORIDE	39.3	34.3	18.5	0.9	50.8	32.7	-17.2	-1.0
PROPARACAINE HYDROCHLORIDE	3.1	-4.9	30.8	0.2	38.6	-4.3	-22.4	-2.9
CYCLOCREATINE	6.7	28.6	33.6	-0.1	42.8	26.9	-17.6	-1.6
PYRITINOL	35.8	14.6	33.0	1.4	43.9	8.4	-17.8	-1.7
ROSOLIC ACID	44.7	29.2	23.8	-2.0	42.0	23.4	-43.1	-3.8
AVOCADYNE ACETATE	-7.5	-3.4	37.4	2.9	38.2	3.7	-6.9	-1.9
ARGININE HYDROCHLORIDE	23.7	12.1	38.3	-1.1	48.2	20.1	-4.7	-0.3
CARZENIDE	15.5	4.9	33.6	4.1	50.1	6.0	-16.2	-2.2
BEPHENIUM HYDROXYNAPTHOATE	32.8	28.1	19.5	1.0	52.8	25.2	-47.0	-1.3
PIMETHIXENE MALEATE	10.3	-4.4	44.5	0.0	24.7	-5.4	-10.5	-2.8
CEFTAZIDIME	-22.8	-10.9	30.2	1.8	29.0	10.4	-20.0	-1.2
3-ACETYLCOUMARIN	19.5	-4.5	33.4	11.7	9.2	-29.8	-12.4	2.4
ARSENIC TRIOXIDE	-50.8	-1.8	32.9	5.1	-12.0	-1.2	-21.7	-1.3
beta-CARYOPHYLLENE ALCOHOL	8.3	10.0	24.9	-1.1	40.2	12.1	-9.2	-2.0
ETHYNODIOL DIACETATE	43.0	32.0	17.1	0.5	42.7	16.7	-41.1	-2.2
PRONETALOL HYDROCHLORIDE	9.8	11.2	28.5	-0.5	48.7	13.4	-5.0	-1.6
THIAMYLAL SODIUM	-2.3	10.8	31.9	0.5	45.8	29.9	-53.5	-1.6
DINITOLMIDE	53.9	27.8	19.7	1.9	50.3	16.1	-24.4	-2.0
DILTIAZEM HYDROCHLORIDE	65.4	38.9	20.4	0.4	45.2	25.9	-26.2	-1.9
IPRONIAZID SULFATE	27.8	10.2	26.1	1.0	53.0	20.1	-31.1	-2.5
AMOXAPINE	46.9	28.9	33.0	0.6	39.2	16.4	-18.6	0.1
CHAULMOSULFONE	3.3	9.6	32.4	-0.4	47.4	6.8	-114.5	-3.0
PENTOXIFYLLINE	58.0	29.0	32.5	1.5	45.9	12.2	-25.6	-1.5
GLUTAMINE (L)	-42.2	3.9	12.8	1.8	39.7	7.0	-36.0	-2.0
ESTRAGOLE	47.6	23.5	29.3	-0.2	25.3	8.9	-26.7	1.1
ACRIFLAVINIUM HYDROCHLORIDE	22.1	-4.0	25.5	0.8	20.5	-2.2	-19.7	7.4
DIBEKACIN	-113.2	17.4	26.2	1.7	-11.3	19.6	-35.7	-2.8
NAFTOPIDIL DIHYDROCHLORIDE	26.5	18.9	18.3	-0.3	40.7	12.6	-58.0	-2.1
EMODIN	70.2	62.4	29.5	1.9	65.5	38.2	-31.8	-1.3
BITOSCANATE	40.4	21.8	23.5	-0.3	42.0	6.7	-32.3	-0.2

PEUCENIN	58.1	67.9	14.2	-0.3	55.4	39.0	-12.6	-1.4
BUCETIN	44.2	19.5	20.8	0.6	36.3	7.5	-34.1	-1.8
BECLAMIDE	42.4	-26.8	-13.1	-3.3	-30.6	-14.8	-49.6	-0.4
CHLOROPYRAMINE HYDROCHLORIDE	31.1	-0.5	-6.1	-0.9	4.9	-13.5	-56.4	-2.5
alpha-CYANO-4-HYDROXYCINNAMIC ACID	68.6	22.4	-11.7	-4.1	11.4	-3.1	-56.3	3.8
PROTOPORPHYRIN IX	6.4	-3.8	-19.8	0.0	21.3	-31.9	-50.4	-0.4
2-AMINOBENZENESULFONAMIDE	40.5	-17.1	10.2	-2.5	24.6	-2.3	-9.3	1.6
CANAVANINE	10.1	-7.7	1.7	-2.5	30.0	-46.6	-28.6	1.5
SPARTEINE HYDROIODIDE	35.5	-19.1	-0.5	-1.9	34.1	5.4	-14.2	2.9
DIHYDROCELASTROL	36.6	-25.7	21.6	-0.4	28.5	-27.6	-76.3	12.9
DIPERODON HYDROCHLORIDE	17.5	-18.8	10.7	-2.4	11.6	-9.8	-7.8	3.8
SULMAZOLE	-0.4	-33.7	1.2	12.1	32.9	-22.2	3.1	17.2
GABAPENTIN	20.5	-14.0	1.3	-0.4	17.4	-7.4	-0.9	5.0
3-ACETAMIDOCOUMARIN	7.8	-27.4	16.6	9.8	30.0	-32.2	6.8	1.6
CLONAZEPAM	-11.0	-23.9	18.4	4.2	12.0	-5.0	-11.4	-1.8
FUSARIC ACID	9.9	-16.7	14.6	47.4	-7.3	-71.3	1.4	22.3
ORNIDAZOLE	13.2	-22.4	7.8	-1.5	9.8	-12.4	-48.9	0.4
HEMICHOLINIUM BROMIDE	10.1	-12.7	3.9	4.2	27.2	-3.9	-66.0	2.8
CHLORDIAZEPOXIDE	32.3	11.2	-5.4	-0.9	26.4	12.0	-59.6	-4.1
TROXERUTIN	24.3	-1.0	4.5	-0.9	38.0	0.6	-7.8	-0.6
GLYBURIDE	21.1	1.1	-3.2	-1.3	35.0	9.7	-33.4	-0.7
DIMETHYL 4,4-o-PHENYLENE-BIS (3-THIOPHANATE)	17.5	-25.9	-0.6	-2.6	49.4	9.6	-14.1	-2.0
BUTYL PARABEN	31.3	-5.6	-6.0	-1.9	47.9	19.5	-31.4	0.2
DEFERIPRONE	13.5	-17.3	21.7	2.0	25.5	-3.9	16.1	3.7
AZTREONAM	22.7	-14.8	16.6	-3.0	25.0	4.8	8.7	1.4
OXALAMINE CITRATE	27.3	-9.1	0.7	14.6	44.1	2.1	-6.7	-0.6
AMINOTHIAZOLE	20.4	-8.2	5.0	-2.8	40.2	17.7	-29.7	-0.8
3-BROMO-4-METHYL-3,4-HEXAMETHYLENE-3,4-DIHYDRODIAZETE-1.2-DIOXIDE	38.3	-2.7	12.6	0.6	30.8	7.4	-9.6	-2.0
CLOPERASTINE HYDROCHLORIDE	9.5	-8.4	23.7	15.7	20.8	8.6	-59.2	0.5
ZARDAVERINE	1.8	-19.0	6.8	27.7	-3.8	-49.1	-5.8	5.5
ETHACRIDINE LACTATE	3.7	-2.8	8.6	-1.0	12.1	2.7	-30.5	4.7
DIACETAMATE	28.6	0.7	2.0	-2.5	36.7	14.1	-37.7	-1.0
5alpha-ANDROSTAN-3,17-DIONE	49.0	12.9	-4.3	-1.7	35.1	22.5	-27.4	-2.6
REBAMIPIDE	40.3	0.6	7.0	-2.2	57.7	22.4	-22.4	-0.7
1,3,5-TRIMETHOXYBENZENE	36.5	4.0	9.5	-1.3	45.0	19.8	-18.6	0.0
6-AMINONICOTINAMIDE	38.7	-0.1	-11.0	-2.6	57.8	20.6	-29.9	0.8
TROLOX	41.7	-8.2	1.1	-2.1	50.7	23.4	-22.5	-0.4
2-THIOURACIL	26.4	-8.3	12.7	-1.9	35.5	4.3	-7.9	0.1

ISAXONINE	24.1	-17.3	17.9	-2.4	23.7	17.6	0.8	-1.0
DIACERIN	34.1	5.1	-5.0	-2.8	56.4	18.0	-36.2	-0.1
3alpha-HYDROXY-3-DEOXYANGOLENSIC ACID METHYL ESTER	30.1	-4.2	3.5	-0.7	41.9	24.5	-10.6	-0.3
TOTAROL-19-CARBOXYLIC ACID, METHYL ESTER	43.1	5.8	17.2	-3.0	30.9	12.2	-26.2	3.9
DIATRIZOIC ACID	6.4	-20.0	14.0	6.8	29.9	12.6	-19.6	3.0
ACETAMINOSALOL	25.1	3.7	15.3	-0.4	-11.4	-13.0	-20.8	0.7
ELETRIPTAN HYDROBROMIDE	32.2	-9.7	18.8	2.0	-4.6	7.6	-37.7	-0.8
GABOXADOL HYDROCHLORIDE	36.4	3.0	2.0	-1.7	34.6	7.9	-44.5	-2.7
BENZBROMARONE	92.7	83.9	44.8	-0.3	72.7	64.4	9.4	-2.2
3-AMINO-beta-PINENE	42.6	2.4	0.0	-2.8	41.3	2.0	-16.8	7.6
OXANTEL PAMOATE	50.0	17.7	6.1	-3.9	48.8	25.9	-29.7	-0.7
HOMIDIUM BROMIDE	89.7	94.0	90.9	77.8	69.5	21.5	-3.7	-2.7
ASTEMIZOLE	46.8	-5.6	20.5	-2.7	46.6	16.0	-32.9	-3.0
PIPENZOLATE BROMIDE	33.6	-2.1	2.7	-2.1	34.0	-2.5	-1.0	1.8
MIANSERIN HYDROCHLORIDE	36.0	5.1	29.9	-3.7	36.8	15.0	-3.7	6.8
LUPININE	36.3	-0.6	2.4	-0.1	38.8	9.6	-14.6	2.1
DECAMETHONIUM BROMIDE	22.0	-2.4	16.3	-1.3	38.9	17.0	-18.0	-2.5
TIRATRICOL	57.0	20.0	9.2	-2.0	28.4	13.6	-19.8	-2.4
CEFAZOLIN SODIUM	30.7	-13.8	28.3	-1.5	37.1	20.3	-17.8	-2.4
URAPIDIL HYDROCHLORIDE	12.3	-27.4	25.9	14.3	12.1	-28.5	-7.4	15.1
BENFOTIAMINE	26.6	-9.1	4.4	-2.5	19.3	8.3	-25.6	2.5
TIOXOLONE	60.4	11.3	5.9	3.6	41.0	13.4	-41.3	-2.2
DIHYDROJASMONIC ACID	55.4	26.8	-6.7	-2.6	49.1	36.6	-25.9	-1.9
CYCLANDELATE	44.3	11.4	21.9	-3.2	52.3	17.5	-32.2	-2.3
meta-CRESYL ACETATE	44.9	10.3	1.8	-4.9	46.9	31.2	-14.7	0.5
METACETAMOL	48.6	-13.4	18.9	-3.9	46.7	17.4	-10.4	-4.5
CHLORQUINALDOL	45.7	8.6	4.6	-3.0	54.3	33.1	-23.1	-2.4
TYROSINE	35.0	-1.1	24.1	-4.2	36.3	12.1	1.9	0.5
3beta-HYDROXY-23,24-BISNORCHOL-5-ENIC ACID	38.7	22.8	15.6	-2.5	24.0	8.5	17.9	0.9
QUINOLINIC ACID	27.2	-13.3	9.2	0.9	44.6	15.1	-24.0	-1.3
SODIUM FLUOROACETATE	22.9	-5.6	1.8	2.3	49.0	29.4	-41.4	-1.0
COTARNINE CHLORIDE	45.4	3.1	18.6	-1.6	34.4	16.6	-8.8	-2.5
PYROGALLIN	45.6	-3.3	19.1	-0.8	27.5	12.3	-15.3	0.3
CRESOPIRINE	31.5	-7.9	13.9	5.2	19.0	-25.8	11.2	25.2
OXIGLUTATIONE DISODIUM SALT	59.7	3.9	4.4	-2.5	12.4	0.7	-18.0	2.7
CHAULMOOGRIC ACID	54.4	16.8	-12.0	-0.9	18.8	-2.3	-64.9	-3.8
PANTOTHENIC ACID(d) Na salt	78.6	50.8	-3.5	-2.8	37.8	24.1	-41.0	-1.2

EBSELEN	70.0	16.7	22.7	-1.9	47.2	15.8	-14.9	-2.3
ARIPIPRAZOLE	57.6	19.1	16.4	-4.0	34.2	18.1	-34.7	-0.9
RAMIFENAZONE	60.0	24.6	6.7	-4.7	60.3	29.1	-11.6	-3.7
BROMPERIDOL	60.3	9.8	14.2	-3.2	48.1	24.3	-8.1	-1.1
PURPUROGALLIN	58.8	25.5	22.0	-4.9	27.7	6.3	6.8	0.7
PROTRYPTYLINE HYDROCHLORIDE	51.7	6.7	18.8	-1.1	30.8	17.6	13.7	-0.4
PREGNENOLONE	48.1	14.6	9.7	-2.0	35.9	10.9	-18.5	-1.2
ACECAINIDE HYDROCHLORIDE	33.9	0.2	9.8	-2.5	41.5	23.4	-16.3	-3.0
CARBIMAZOLE	54.7	12.1	9.5	-2.3	28.4	13.6	-12.1	-0.7
VESAMICOL HYDROCHLORIDE	29.2	-7.2	25.4	-0.7	27.6	21.7	-34.7	-0.9
TETRACHLOROISOPHTHALONITRILE	33.2	-44.0	30.6	12.0	11.0	-49.3	-138.7	0.1
CARBADOX	30.4	0.1	13.0	-1.8	39.7	2.6	-28.3	-4.7
CARSALAM	3.7	9.9	36.1	-2.5	6.8	16.9	-2.3	-0.9
TUBOCURARINE CHLORIDE	55.5	22.1	9.7	-4.2	38.6	14.3	-35.8	-3.7
TOSYLCHLORAMIDE SODIUM	51.5	20.5	17.2	-3.7	44.1	33.0	-21.5	-4.8
AMINOPTERIN	30.0	-0.8	17.6	-3.8	45.3	32.4	-41.3	-2.4
DEOXYCHOLIC ACID	46.3	11.2	-6.2	-5.2	55.8	26.1	-97.3	-2.6
PENTAMIDINE ISETHIONATE	48.2	7.0	18.3	-3.4	53.4	30.1	-18.8	-2.5
HALOTHANE	49.0	-1.7	17.5	-4.1	36.4	9.4	11.1	0.0
PURPURIN	43.5	-2.3	-2.0	-4.3	29.8	-0.2	9.2	2.2
DEACETOXY(7)-7-OXOKHIVORINIC ACID	47.9	5.9	9.8	-4.5	46.0	23.1	-17.7	-1.4
ORBIFLOXACIN	98.7	97.1	97.8	34.8	78.7	66.4	-9.7	-2.0
PRISTIMERIN	46.6	0.0	17.8	-2.6	17.7	5.7	-19.9	-1.3
PHYSCION	13.6	-7.6	23.5	-2.0	34.7	20.5	-7.1	-1.6
CITIOLONE	10.7	-13.4	3.8	10.2	15.9	-29.9	6.4	14.1
HYDROQUININE HYDROBROMIDE HYDRATE	1.8	-12.3	21.9	3.0	-19.4	21.3	-32.1	-2.4
DIHYDROGEDUNIC ACID, METHYL ESTER	60.1	21.1	13.2	-2.2	37.5	17.4	-33.2	-4.5
METHYL 7-DESHYDROXYPYROGALLIN-4-CARBOXYLATE	45.5	22.8	11.4	-0.7	58.0	49.5	-35.4	-2.5
DIHYDROCELASTRYL DIACETATE	57.5	20.6	44.6	0.5	38.5	9.8	-121.7	-0.2
POMIFERIN	52.5	22.8	26.2	-2.7	35.9	33.2	-6.4	-2.9
NORSTICTIC ACID	27.5	-11.7	21.1	-1.1	50.7	15.5	-60.7	-4.4
DESONIDE	40.0	2.3	-0.4	0.5	49.0	35.1	1.6	-2.5
ACETYL-L-LEUCINE	33.3	0.4	23.2	-2.6	30.6	1.0	11.2	1.1
ZILEUTON	35.0	2.7	16.3	-3.3	33.0	19.5	0.8	-0.8
METHOPRENE (S)	12.3	-24.1	27.2	-1.3	44.4	16.0	-16.5	-0.1
CYPROHEPTADINE HYDROCHLORIDE	35.8	-6.7	21.3	-2.7	35.8	27.0	-14.3	-2.6
METERGOLINE	21.9	-13.1	16.4	-0.9	36.7	17.8	-8.8	-4.0
PHYTONADIONE	39.2	-10.6	18.5	0.0	35.8	13.3	5.4	1.2
PHENOTHRIN	13.0	-10.6	3.8	-1.6	8.9	-29.5	-140.1	1.0

FLUROTHYL	5.3	-4.8	0.8	-1.7	-8.3	-0.6	-25.5	-1.7
NIFENAZONE	73.8	40.1	-8.0	-2.8	20.4	41.5	-27.4	-3.5
NIZATIDINE	64.4	33.8	9.7	-2.8	37.5	29.5	-35.2	-2.8
SARMENTOSIDE B	70.0	34.3	-0.9	-2.2	51.0	18.5	-64.9	-0.6
ENROFLOXACIN	99.4	98.9	98.9	64.9	84.4	62.5	39.3	-2.8
beta-NAPHTHOL	71.6	35.2	-1.3	-2.1	55.8	23.8	-15.3	-1.5
TOLMETIN SODIUM	66.5	39.1	2.6	-2.1	41.8	20.3	-62.4	-4.1
CINCHOPHEN	52.0	20.1	12.2	-2.3	31.1	8.3	9.4	-0.8
RUTILANTINONE	53.3	9.6	11.3	-4.2	26.1	7.5	5.7	1.2
CHLORINDIONE	44.3	1.1	4.8	0.2	41.1	15.4	-10.6	-1.9
HYDRASTININE HYDROCHLORIDE	33.1	-4.5	23.8	-1.8	39.8	21.1	-23.6	-1.8
VINCAMINE	26.4	-10.0	7.0	-1.3	37.5	6.7	-4.5	-1.7
FOSFOSAL	35.1	-4.9	23.2	-3.7	28.4	14.2	-3.6	0.6
MIZORIBINE	14.1	-24.5	14.8	2.9	16.5	-14.4	4.5	5.0
ZAPRINAST	44.7	-13.5	7.0	-2.0	-5.6	4.4	-20.7	0.3
LIPOAMIDE	73.8	55.3	-4.6	-4.1	30.8	7.5	-41.7	-1.9
RIBOSTAMYCIN SULFATE	58.4	27.8	8.8	-2.3	42.0	34.0	-19.7	-4.2
STROPHANTHIDINIC ACID LACTONE ACETATE	62.0	16.8	2.8	-5.2	38.5	10.1	-56.4	-4.4
OXYTHIAMINE CHLORIDE HYDROCHLORIDE	72.2	34.2	11.6	-3.4	49.9	9.4	-57.6	-1.6
SUMATRIPTAN	55.3	15.4	9.5	-4.0	42.8	18.9	-6.9	-3.5
CYCLOBENZAPRINE HYDROCHLORIDE	43.9	12.8	17.0	-3.5	46.7	26.2	-31.4	-4.1
MEPIROXOL	38.7	4.9	21.9	-2.7	31.6	9.0	5.8	1.3
GLYCOCHOLIC ACID	37.8	-6.6	20.9	0.6	33.8	17.5	3.7	-0.9
2,6-DIMETHOXYQUINONE	49.5	9.4	4.4	0.5	40.2	22.2	10.2	2.7
DECAHYDROGAMBOGIC ACID	27.9	-6.0	14.1	-1.6	40.5	26.7	-21.6	0.9
ROCCELLIC ACID	48.8	16.6	1.8	-0.9	23.9	13.1	7.5	-0.4
KHAYANTHONE	30.3	-10.1	33.9	7.4	25.9	15.6	-10.6	-1.9
IRETOL	16.2	-11.6	20.7	4.4	21.2	-15.0	7.9	22.5
3-DEACETYLKHIVORIN	27.4	-10.0	7.6	-2.2	-14.1	14.0	-21.5	-1.3
DEHYDROROTENONE	53.3	29.4	7.5	-4.3	37.7	16.1	-34.0	-2.6
1,2alpha-EPOXYDEACETOXYDIHYDROGEDUNIN	70.9	49.5	-1.8	-3.8	42.6	35.0	-57.0	-4.6
MUNDOSERONE	39.8	10.8	10.3	-3.8	47.0	17.0	-34.8	-2.0
3-DEOXO-3beta-ACETOXYDEOXYDIHYDROGEDUNIN	51.2	11.7	8.2	-4.6	37.2	28.0	-33.0	-2.0
ORSELLINIC ACID	16.9	6.5	16.4	-4.3	48.7	18.9	-5.6	-2.7
DIHYDROGEDUNIN	52.9	17.2	21.8	-5.8	50.7	35.2	-10.5	-3.1
1-MONOPALMITIN	32.0	3.8	20.4	-4.0	37.5	18.0	2.4	1.4
ALPINETIN METHYL ETHER	44.8	7.2	26.5	-3.1	33.3	21.1	-5.3	-1.1

2-HYDROXY-5 (6)EPOXY-TETRAHYDROCARYOPHYLLENE	34.0	0.0	18.8	-1.9	46.1	17.9	-2.9	1.4
ARTEMISININ	35.3	-4.9	16.7	-0.5	34.4	28.0	-11.9	-0.9
HYMECROMONE METHYL ETHER	25.8	-7.0	14.3	0.0	33.1	13.4	6.2	-2.2
PALMATINE CHLORIDE	27.6	-5.7	31.7	-1.6	39.6	28.9	-12.5	0.9
CATECHIN TETRAMETHYLETHER	18.3	-14.2	12.4	6.2	20.6	-8.9	-7.4	12.7
PANTETHINE	-6.3	2.3	3.4	-2.6	-6.5	0.4	-10.6	0.2
VISNAGIN	78.5	55.5	13.1	-5.0	39.2	9.0	-34.3	-4.1
LAPACHOL	56.6	20.0	12.9	-4.1	46.3	24.0	-29.3	-0.9
CHRYSANTHEMIC ACID, ETHYL ESTER	57.6	33.3	26.7	-2.5	53.5	34.6	-25.0	-1.8
SPAGLUMIC ACID	35.8	10.5	19.3	-4.1	35.0	28.1	-24.9	-1.1
4-HYDROXY-6-METHYLPYRAN-2-ONE	66.8	33.7	18.5	-4.6	63.1	32.2	-15.5	-0.4
2,6-DIHYDROXY-4-METHOXYTOLUENE	39.7	4.9	24.4	-3.1	53.1	39.2	-12.6	-1.7
PAEONOL	39.6	-2.3	24.7	-3.6	35.1	14.3	0.2	0.8
alpha-DIHYDROGEDUNOL	37.3	4.9	22.7	-1.8	19.8	9.6	-3.0	1.8
DERRUSNIN	42.7	10.9	23.1	-2.5	43.7	12.4	-23.0	0.9
2-METHYL-5,7,8-TRIMETHOXYISOFLAVONE	28.2	4.1	17.9	-1.9	44.4	29.5	-14.1	-0.6
5,7,4'-TRIMETHOXYFLAVONE	42.0	-7.1	8.1	-0.4	30.1	11.2	-94.0	-1.9
MUNDULONE	5.8	9.7	21.1	1.3	27.3	12.9	-71.7	-0.4
3,7-DIMETHOXYFLAVONE	38.9	-3.2	9.3	-1.6	11.7	-28.6	-81.6	3.1
OSAJIN	24.2	14.5	7.8	-2.6	-12.4	12.3	-20.4	-1.3
METHYLXANTHOXYLIN	61.5	22.4	16.1	-1.6	35.2	11.6	-36.4	-2.1
BRAZILEIN	72.2	47.7	14.1	-2.9	65.8	57.9	-13.9	-5.6
IRIGENIN, 7-BENZYL ETHER	51.9	24.1	18.1	-1.3	35.3	14.5	-15.3	-2.5
7-DEACETYLKHIVORIN	65.0	25.3	19.2	-5.7	48.3	27.8	-40.1	-2.0
DUARTIN (-)	45.5	24.9	36.2	-4.7	42.8	23.1	-13.6	-1.6
8beta-HYDROXYCARAPIN, 3,8-HEMIACETAL	48.2	28.5	21.7	-3.5	60.7	45.0	-45.7	-2.9
PACHYRRHIZIN	32.8	1.8	16.2	-3.8	27.2	9.8	-8.0	0.7
MEROGEDUNIN	40.6	5.6	23.6	-5.3	37.6	27.7	-1.0	-1.2
SOLIDAGENONE	27.4	0.0	31.1	-0.7	38.6	10.8	-22.8	-0.1
TOTAROL	49.7	-1.4	42.2	-2.1	27.7	28.2	-22.6	0.0
EPI(13)TORULOSOL	52.2	13.3	8.4	-1.3	21.3	12.7	-7.6	-1.7
SALIDROSIDE	36.2	-3.1	31.2	0.3	18.2	20.3	16.0	-1.8
3,7-EPOXYCARYOPHYLLAN-6-ONE	31.9	-7.0	16.3	-0.9	17.7	-26.4	-8.8	0.1
7,4'-DIHYDROXYFLAVONE	-9.1	-11.3	16.6	-1.5	-23.1	16.3	-18.7	-1.5
XANTHOPTERIN	75.9	51.5	-3.5	-4.8	37.1	18.3	-27.6	-3.7
CRYPTOTANSHINONE	77.4	62.5	-13.2	-1.9	27.5	44.1	-121.8	-3.0
ACACETIN	58.2	38.8	11.0	-2.7	42.7	24.9	-80.3	-3.6
CARNOSINE	48.2	32.9	11.0	-0.9	43.0	34.8	-20.8	-2.9
GLUCOSAMINIC ACID	62.7	25.2	24.3	-2.6	49.7	20.9	-26.2	-6.1

PATULIN	45.9	25.4	23.7	-2.7	60.1	41.7	-18.3	-4.0
ANABASINE HYDROCHLORIDE	47.8	9.6	26.5	-1.8	31.0	11.5	0.3	-0.7
7-DESHYDROXYPYROGALLIN-4-CARBOXYLIC ACID	44.7	16.2	22.9	-2.7	32.1	19.2	-0.5	0.5
2-HYDROXY-3,4-DIMETHOXYBENZOIC ACID	43.6	7.7	31.8	-0.7	53.7	24.9	-24.2	-0.7
QUERCETIN PENTAMETHYL ETHER	50.0	9.5	6.3	2.7	40.0	17.6	-5.6	0.9
HEPTAMINOL HYDROCHLORIDE	39.2	16.1	23.3	-2.7	45.9	21.9	11.9	-1.2
DIHYDRODEOXYGEDUNIN	59.3	7.5	10.0	0.0	27.5	14.4	-17.8	-0.9
DERRUSTONE	23.9	-3.8	25.7	18.4	10.4	-22.3	-28.8	0.8
GRISEOFULVIC ACID	45.6	12.5	3.0	-3.8	-9.6	19.0	-19.2	-2.0
PHLORACETOPHENONE	46.9	42.9	0.9	0.2	38.5	11.3	-39.1	-2.9
BRAZILIN	78.6	66.2	17.5	-5.3	60.2	56.0	-7.9	-4.5
4-METHYLDAPHNETIN	42.4	30.6	16.5	-3.9	41.8	20.0	-29.0	-4.5
BISANHYDRORUTILANTINONE	51.7	41.5	17.6	-4.1	45.4	40.1	-26.5	-1.7
ANHYDROBRAZILIC ACID	36.9	6.5	21.5	-2.0	53.8	26.3	-4.3	-3.4
CARAPIN	43.2	16.0	17.7	-4.2	45.7	34.1	-15.7	-1.5
IRIDIN	27.7	0.9	18.5	-2.2	31.4	10.3	-3.6	-1.1
1,7-DIDEACETOXY-1,7-DIOXO-3-DEACETYLKHIVORIN	32.6	12.7	23.9	-3.1	36.0	24.6	-15.5	1.3
IRIGENIN, DIBENZYL ETHER	-2.7	-22.7	27.6	-3.3	40.8	16.5	-22.8	1.8
3,16-DIDEOXYMEXICANOLIDE-3beta-DIOL	20.0	-9.7	17.8	-2.5	36.4	26.9	-23.7	-0.7
D-PERSEITOL	32.7	-14.2	19.9	-0.8	28.4	13.1	-4.0	3.0
ENTANDROPHRAGMIN	22.5	-11.1	17.1	-1.9	22.4	21.6	-8.2	-1.5
SPHONDIN	31.0	-13.4	4.4	-0.6	20.2	-15.2	-0.9	11.0
FISSINOLIDE	43.1	8.3	-1.8	-2.3	6.5	39.1	-22.5	-2.4
CADIN-4-EN-10-OL	75.0	49.6	1.6	-3.8	31.4	15.9	-41.2	-1.4
N-METHYLBENZYLAMINE HYDROCHLORIDE	80.3	64.6	-1.7	-2.5	64.3	58.6	-21.9	-4.3
CLOVANEDIOL DIACETATE	51.6	39.6	12.7	-2.4	46.0	11.6	-24.9	-1.9
LOBELINE HYDROCHLORIDE	47.1	27.6	18.8	-3.4	43.4	31.2	-34.0	-2.8
SANTONIN	54.8	30.4	13.3	-4.9	49.9	27.2	1.2	-2.9
XANTHURENIC ACID	40.9	5.9	22.4	-1.5	49.8	38.2	0.6	-5.0
EPICATECHIN PENTAACETATE	39.9	15.4	22.0	-1.3	31.4	15.0	8.8	1.1
GLUTAMINE (D)	25.1	4.6	23.0	-3.9	50.2	34.6	-25.4	-1.6
RHODINYL ACETATE	45.4	4.1	11.5	-0.7	30.7	10.7	16.3	0.2
ENOXOLONE	25.2	11.6	13.8	-0.9	28.0	18.5	-6.7	-1.6
ACONITIC ACID	42.1	6.8	14.3	4.8	40.2	13.4	-4.9	2.4
6-METHOXYHARMALAN	25.9	-0.1	25.3	4.7	17.2	16.6	4.1	-0.4
HAEMATOMMIC ACID	24.0	4.2	27.6	16.2	13.3	-9.7	-13.6	2.2
3-METHOXYCATECHOL	43.7	0.5	3.1	-4.4	-10.9	3.8	-37.7	-3.1
5alpha-CHOLESTAN-3beta-OL-6-ONE	66.8	52.7	4.7	-5.3	35.7	11.7	-20.9	-4.7

CHOLESTAN-3-ONE	58.9	46.9	9.1	-3.2	51.6	44.2	-24.6	-4.0
METHYL ROBUSTONE	50.3	40.2	16.9	-4.7	36.3	18.9	1.0	-4.7
2,3,4'-TRIHYDROXY-4-METHOXYBENZOPHENONE	56.2	33.7	7.0	-1.8	49.9	53.1	-41.1	-3.1
CARNITINE (dl) HYDROCHLORIDE	37.6	5.5	22.5	-0.3	54.8	36.1	-20.2	0.5
FUMARPROTOCETRARIC ACID	71.0	35.9	23.5	-1.7	59.8	53.9	4.5	-0.8
CHRYSIN DIMETHYL ETHER	30.8	14.2	5.8	-0.8	24.2	10.7	-35.0	-2.7
HAEMATOMMIC ACID, ETHYL ESTER	38.5	18.2	16.3	-0.5	38.0	30.3	-20.7	0.8
DEOXYSAPPANONE B 7,4'-DIMETHYL ETHER	33.2	8.7	14.1	-0.8	49.4	25.8	-34.0	-1.7
SHIKIMIC ACID	42.6	17.0	6.0	-1.3	46.8	37.4	-18.5	-1.2
DALBERGIONE, 4-METHOXY-4'-HYDROXY-	39.4	1.9	18.0	-1.8	33.6	13.4	-20.4	-2.6
PICROTIN	53.1	10.8	25.7	-1.6	27.9	18.0	0.7	-1.3
NONIC ACID	19.8	3.0	26.8	1.6	13.5	-12.4	-5.8	3.4
TOTAROL ACETATE	41.9	-1.3	2.8	-1.2	-13.3	25.0	-22.4	-2.2
EPIAFZELECHIN (2R,3R)(-)	74.5	54.3	-0.4	-3.5	55.4	41.8	-46.3	-1.6
1,3-DIDEACETYL-7-DEACETOXY-7-OXOKHIVORIN	73.0	54.1	8.0	-2.6	37.9	43.6	-30.0	-1.3
EUPARIN	43.0	29.1	6.4	-2.1	44.6	20.2	-29.6	-3.3
DEOXYGEDUNIN	48.3	27.1	18.0	-3.0	43.1	37.6	-16.2	-1.0
LARIXOL	38.8	27.6	18.4	-0.8	46.9	32.3	-46.8	-2.1
3-DESHYDROXYSAPPANOL TRIMETHYL ETHER	51.9	11.9	28.3	-1.8	51.9	47.3	-16.5	-1.7
3-OXOURSAN (28-13)OLIDE	43.8	14.9	12.2	-2.2	35.1	22.4	-52.9	-2.6
4-O-METHYLPHLORACETOPHENONE	46.1	21.1	17.7	-1.0	33.3	30.7	-4.4	1.7
PHLORIDZIN	34.4	11.2	11.5	0.2	40.2	21.7	-55.0	-0.7
RUBESCENSIN A	22.8	-1.9	8.4	-1.2	41.1	35.9	-102.8	-1.7
EPIGALLOCATECHIN	33.0	5.1	28.0	0.0	37.3	17.3	-20.5	-2.7
CADAVERINE TARTRATE	34.6	-1.9	29.5	-0.4	28.9	31.7	-7.6	1.2
HELENINE	13.6	-7.3	21.4	4.0	15.6	-8.1	-35.3	-1.2
TANSHINONE IIA	52.1	22.9	0.4	-3.4	-5.2	-1.6	-18.1	-2.6
HESPERIDIN	69.5	52.4	-4.1	-2.4	45.6	22.6	-34.7	-3.5
ANDROSTERONE ACETATE	75.4	59.5	0.8	-3.0	41.9	40.4	-30.3	-0.5
ISOPEONOL	47.2	29.6	16.6	-1.0	35.9	16.8	-2.9	-2.6
MENAQUINONE-4	51.4	37.9	15.9	-2.4	36.8	34.2	-16.0	-1.7
PERSITOL HEPTAACETATE	43.1	8.4	16.0	-1.4	46.2	29.8	-10.5	-1.6
SPERMINE	50.6	23.7	21.3	-4.8	45.3	32.4	0.3	-3.7
7-DEACETOXY-7-OXOKHIVORIN	36.8	5.9	17.0	-1.9	33.6	16.7	3.5	-3.3
KOPARIN	34.0	17.5	24.9	-3.6	36.5	29.0	-7.6	-0.4
QUERCETIN	32.2	0.4	16.4	0.0	37.1	15.5	-1.8	-1.0
GANGALEOIDIN	47.0	33.3	29.2	9.2	48.6	47.6	-5.2	-0.6

NARINGENIN	26.3	-7.6	15.9	-2.6	27.6	8.9	6.5	-3.0
LANOSTEROL ACETATE	13.1	-2.8	24.1	-1.0	30.4	30.3	-2.1	-0.2
XANTHONE	3.2	7.1	15.9	15.0	11.2	-21.2	-3.0	0.1
PODOPHYLLIN ACETATE	12.0	47.9	-6.1	-1.2	4.3	47.1	-35.9	-1.4
ACACETIN DIACETATE	77.1	58.0	2.3	-3.9	36.7	16.8	-57.0	-2.9
3-DEOXO-3beta-HYDROXYMEXICANOLIDE 16-ENOL ETHER	72.3	60.3	-3.1	-1.0	53.4	55.4	-43.0	-0.9
DALBERGIONE	67.2	45.1	11.4	-2.8	32.0	9.8	-3.2	-1.9
HYDROLYSIS PRODUCT OF BUSSEIN	63.2	49.7	13.5	-2.2	41.6	42.5	-25.2	-1.6
EVERNINIC ACID	52.8	21.3	20.2	2.9	55.2	27.3	-9.9	-2.3
3beta-ACETOXYDEOXODIHYDROGEDUNIN	61.8	38.1	14.4	-1.9	44.6	35.3	-13.2	-1.6
STICTIC ACID	49.7	31.9	14.8	-1.5	29.6	11.8	-16.7	-3.0
DEOXYKHIVORIN	34.5	23.0	18.1	-1.9	32.5	28.0	-10.2	0.4
LARIXOL ACETATE	29.9	-3.2	12.1	11.5	28.0	-4.3	3.4	-3.6
beta-ESCIN	23.3	-2.1	26.0	-4.2	34.5	28.7	-9.2	-1.3
DEHYDROABIETAMIDE	31.5	0.1	12.5	-0.6	24.5	0.6	-39.7	-3.7
ARCAINE SULFATE	30.4	-0.6	22.5	2.5	26.7	33.6	12.9	-0.5
GEDUNOL	7.2	3.6	24.9	14.0	4.3	-29.2	8.3	-1.2
FERULIC ACID	-36.8	31.4	-1.2	-2.6	-18.8	4.5	-21.5	-1.6
CATECHIN PENTAACETATE	65.2	65.9	2.7	-4.4	32.0	13.6	-38.2	-1.0
ELLAGIC ACID	40.9	41.5	11.0	-1.5	48.8	51.7	-37.2	-1.4
HESPERETIN	25.2	33.0	1.1	-2.4	34.0	12.9	-18.8	0.1
PEONIFLORIN	49.7	35.8	17.2	-2.2	36.2	38.5	-45.7	-0.4
LARIXINIC ACID	48.7	34.9	3.4	-3.4	39.4	20.6	-8.7	-3.0
alpha-HYDROXYDEOXYCHOLIC ACID	38.0	20.4	-8.6	-3.0	-18.7	5.3	-20.9	4.0
N-METHYLISOLEUCINE	36.5	9.0	-0.9	-0.4	36.8	-7.0	-54.5	-9.1
GRAMINE	51.5	22.4	-4.6	-8.0	34.7	20.3	-50.7	-4.9
3,4-DIDESMETHYL-5-DESHYDROXY-3'-ETHOXYSCLEROIN	17.8	-7.4	9.6	2.6	39.4	-2.1	6.3	-7.4
HETEROPEUCENIN, METHYL ETHER	43.4	19.4	-7.2	-11.2	40.2	19.6	-43.7	-3.6
DESACETYL (7)KHIVORINIC ACID, METHYL ESTER	39.2	0.5	-4.6	0.0	55.4	6.0	-51.7	-7.8
2,3-DIHYDROXY-4-METHOXY-4'-ETHOXYBENZOPHENONE	54.5	19.4	-16.0	-11.3	51.4	27.1	-40.7	-2.5
gamma-AMINOBUTYRIC ACID	24.4	-18.2	14.5	5.9	30.3	-10.2	-2.8	-6.8
LECANORIC ACID	36.3	6.2	7.9	-11.6	28.7	13.9	17.5	-0.4
QUERCITRIN	30.7	-20.8	-15.7	7.6	40.8	2.6	-84.9	-7.8
7-AMINOCEPHALOSPORANIC ACID	36.2	-10.7	-4.5	-1.6	49.7	15.6	-28.4	-8.8
3-METHYLORSELLINIC ACID	26.3	-33.7	10.9	17.7	31.2	-2.0	-15.5	-9.2
DIFUCOL HEXAMETHYL ETHER	24.5	-13.4	-2.5	9.3	30.5	10.2	-9.7	-9.0

APIGENIN	-17.0	-31.8	1.7	-3.5	-6.0	-11.8	-24.8	2.1
EPOXYGEDUNIN	26.5	7.0	-1.1	-3.5	2.7	1.3	-25.1	-7.0
2'-METHOXYFORMONETIN	73.3	38.4	0.9	-9.6	49.0	35.7	-64.0	-4.8
OLEANOIC ACID	48.7	28.6	-12.1	-9.8	52.8	46.5	-97.1	-7.5
EPIAFZELECHIN TRIMETHYL ETHER	71.2	38.5	7.7	-11.2	50.1	34.2	-29.0	-5.8
3beta-HYDROXYISOALLOSPIROST-9(11)-ENE	45.8	26.8	2.0	-9.5	40.8	29.3	-43.9	-1.0
AGELASINE	100.7	97.4	100.3	96.4	55.8	28.7	-51.4	-7.4
SMILAGENIN ACETATE	34.8	-11.2	-3.1	-10.1	53.0	31.6	-20.5	-2.8
VULPINIC ACID	55.1	21.7	10.8	-11.3	34.3	19.2	11.4	-7.9
6,4'-DIMETHOXYFLAVONE	40.7	-0.3	10.2	-9.9	30.8	21.1	-11.8	-6.5
MUUROLLADIE-3-ONE	54.6	-2.5	-2.0	-6.3	52.1	19.0	-25.3	-2.4
3,4-DIHYDROXYCARANE	28.1	-0.7	1.1	-10.5	58.4	32.3	-25.0	-4.5
CHRYSANTHEMYL ALCOHOL	48.9	7.8	3.6	-5.4	48.2	14.5	-33.7	-2.2
GOSSYPOL	46.3	10.5	23.7	-5.4	48.4	21.3	-24.0	9.7
EPIGALLOCATECHIN 3,5-DIGALLATE	50.4	29.6	-32.7	-9.9	-12.7	-6.1	-35.8	-8.6
CAMPTOTHECIN	16.7	-1.9	7.1	0.7	25.6	11.4	-9.9	-7.2
EPIANDROSTERONE	35.8	8.1	3.8	-4.0	51.5	38.9	-53.5	-7.8
7-DESACETOXY-6,7-DEHYDROGEDUNIN	52.1	45.6	1.9	-6.5	55.9	42.8	-35.8	-7.5
QUINIC ACID	44.3	13.1	9.9	-6.9	47.1	36.5	-31.2	-4.8
BIOCHANIN A	41.8	37.7	0.9	3.0	56.1	44.0	-35.2	-4.8
CREATININE	42.9	-25.8	2.8	-5.9	61.3	35.0	-39.1	-6.0
ESEROLINE FUMARATE	52.2	23.0	0.8	-8.1	60.6	37.8	-29.3	-6.0
PIPERONYLIC ACID	37.1	2.0	18.7	10.8	37.2	18.3	7.5	-4.6
PECTOLINARIN	39.3	20.9	13.7	-6.0	43.5	27.7	9.7	-5.8
FRAXIDIN METHYL ETHER	37.6	-0.9	-8.2	-10.1	67.4	33.4	-34.1	-5.6
OBTUSAQUINONE	49.6	13.3	-6.3	-11.7	50.0	28.3	-32.4	-4.8
MELATONIN	36.9	6.7	7.5	5.2	64.6	25.0	-11.6	0.1
GITOXIGENIN DIACETATE	29.3	-2.7	3.7	-1.2	41.3	15.1	-18.3	6.7
KINETIN	19.0	-20.2	11.8	14.6	-20.6	6.5	8.8	-6.2
GARCINOLIC ACID	33.1	3.1	-1.4	-7.6	10.9	0.3	-21.2	11.0
2-BENZOYL-5-METHOXYBENZOQUINONE	56.3	25.4	-0.8	-10.3	54.5	39.5	-41.4	-9.5
ANTHOTHECOL	65.2	47.4	4.6	-11.1	51.2	41.1	-28.3	-2.2
DEOXYSAPPANONE B 7,3'-DIMETHYL ETHER	59.0	26.2	-8.5	-4.5	59.2	39.9	-36.2	-7.7
PRENYLETIN	38.5	13.1	0.8	-10.7	44.1	33.5	-15.6	-4.8
DEOXYSAPPANONE B TRIMETHYL ETHER	54.0	6.1	-3.6	-13.2	65.3	27.2	-45.1	-8.3
LUNARINE	45.7	5.9	-2.7	-9.1	59.5	39.4	-32.6	-8.6
GENISTEIN	37.9	12.5	7.2	-7.3	38.4	16.9	16.1	-3.0
DIPTERYXIN	35.8	-0.8	18.4	3.9	38.2	30.0	10.4	-9.0
ISOPIMPINELLIN	45.0	-6.3	-10.0	-6.4	64.2	38.8	-22.8	-6.2
DEACETOXY-7-OXOGEDUNIN	31.5	-2.8	-3.6	-12.4	61.6	39.8	-36.4	-0.1

3-NOR-3-OXOPANASINSAN-6-OL	41.7	4.7	-2.1	-10.3	50.7	29.9	-14.1	-8.9
CARNOSIC ACID	24.5	-1.6	5.3	4.3	43.6	6.3	-47.6	-8.7
3-PINANONE OXIME	17.3	-27.3	5.6	-2.3	4.8	-0.9	-3.0	-5.6
MUNDULONE ACETATE	1.3	0.9	7.7	2.7	11.4	6.3	-10.7	4.4
MENTHONE	64.9	40.8	4.3	-10.6	59.8	40.9	-59.6	-7.2
BERBERINE CHLORIDE	53.5	36.9	5.9	-8.7	60.5	53.7	-37.6	-6.5
EPICATECHIN	50.9	29.5	10.0	-13.2	55.2	42.3	-30.0	-9.3
BAICALEIN	51.2	23.7	1.4	-6.8	52.9	43.1	-44.9	-6.7
DJENKOLIC ACID	53.2	16.5	3.3	-12.8	61.6	31.7	-27.6	-7.8
HARMINE	53.2	21.9	3.4	-7.6	50.0	35.8	-18.3	-7.1
QUASSIN	44.5	10.0	16.0	-9.6	46.9	20.9	-6.7	-7.9
METHYL DEOXYCHOLATE	39.6	6.7	19.5	-1.3	29.9	21.9	-46.0	-6.8
MEVASTATIN	46.1	-4.4	-8.8	4.8	64.8	27.7	-20.3	-8.4
GEDUNIN	27.2	6.4	-6.4	-9.9	47.5	20.1	-6.6	-5.0
PHENACYLAMINE HYDROCHLORIDE	46.3	9.8	8.8	7.7	56.0	16.1	-19.8	-7.4
ISOTECTORIGENIN, 7-METHYL ETHER	26.4	-4.3	10.9	-6.4	42.8	5.6	7.0	-6.1
DIHYDROXY (3alpha,12alpha)PREGNAN-20-ONE	45.0	-4.8	3.7	-9.8	9.7	-3.9	-11.6	-6.4
PISCIDIC ACID	37.3	1.6	16.1	-5.3	5.4	21.7	-9.0	9.5
CITROPTEN	48.1	12.1	3.5	-12.6	35.5	25.3	-29.9	-4.9
2-METHYL GRAMINE	62.8	48.2	-1.9	-10.4	57.1	54.7	-46.5	-6.8
NARINGIN	50.5	21.7	1.3	-6.2	51.9	31.9	-38.6	-6.9
GALLIC ACID	50.9	25.7	13.8	-11.1	46.3	40.9	-20.3	-6.0
ERGOSTEROL	55.1	16.0	4.4	-14.2	55.0	28.6	-42.0	-8.7
CEDRELONE	54.1	25.6	37.3	-12.2	52.7	31.8	-20.4	-7.1
IRIGENIN TRIMETHYL ETHER	45.0	20.1	11.7	-11.0	43.6	18.8	27.2	-1.6
MEXICANOLIDE	42.3	12.2	14.3	-3.5	41.0	31.9	10.4	-7.3
DEOXYSAPPANONE B 7,3'-DIMETHYL ETHER ACETATE	45.2	-7.8	0.1	-9.0	55.8	21.2	-9.1	-0.7
FRIEDELIN	47.8	2.4	2.7	-12.5	49.8	31.6	-21.7	-9.0
ISOOSAJIN	41.2	4.5	9.8	-1.8	51.5	9.9	-13.6	-7.6
PODOTOTARIN	41.1	5.0	14.8	-0.3	39.8	16.9	-20.6	-3.6
ISOBERGAPTENE	19.2	-15.4	5.3	-2.2	35.4	-3.2	2.4	-4.5
KHIVORIN	45.8	7.3	12.5	-12.7	49.3	27.4	-29.0	-8.1
2-METHOXY-5 (6)EPOXY-TETRAHYDROCARYOPHYLLENE	44.3	-1.1	12.1	0.8	1.7	22.9	-22.2	-8.2
BOVINOCIDIN (3-nitropropionic acid)	55.4	31.1	10.0	-13.0	55.9	39.1	-53.8	-10.1
MELEZITOSE	74.6	60.3	-3.8	-12.6	60.1	53.5	-34.7	-7.4
AJMALINE	66.7	52.7	8.8	-11.6	48.1	40.1	-28.8	-6.5
CHRYSAROBIN	56.2	8.9	2.3	-11.7	58.6	26.6	-37.6	-8.5

AGMATINE SULFATE	70.3	44.0	10.2	-10.8	53.0	36.6	-24.5	-7.6
EPIGALLOCATECHIN-3-MONOGALLATE	27.5	14.3	-2.1	-9.4	61.3	38.9	-24.6	-7.8
BERGENIN	48.4	15.2	8.3	-9.3	42.6	32.1	6.3	-7.1
CEDRYL ACETATE	48.8	3.9	-2.5	-9.1	50.1	24.9	-26.0	-9.2
CARAPIN-8(9)-ENE	46.8	4.1	0.1	-6.1	45.0	31.3	-16.4	-6.2
APIIN	33.7	-5.1	12.1	-6.0	41.6	14.6	-29.2	-7.7
JUGLONE	44.4	3.9	13.4	-6.9	35.1	25.9	-19.8	-6.6
N-METHYLANTHRANILIC ACID	14.9	-16.1	6.8	-4.7	19.8	-17.1	-3.3	3.8
3alpha-HYDROXY-4,4-BISNOR-8,11,13-PODOCARPATRIENE	43.1	17.2	2.1	-12.8	21.7	13.1	-26.6	-5.9
CYCLOVERATRYLENE	76.1	43.7	-12.6	-12.5	33.6	17.7	-37.9	-7.2
2',4'-DIHYDROXYCHALCONE 4'-GLUCOSIDE	73.1	58.9	10.2	-11.0	54.5	49.6	-42.2	-7.4
LEOIDIN	83.6	69.2	32.5	-10.3	62.2	55.9	-11.6	-6.0
ANTIAROL	54.2	44.2	6.9	-11.8	53.8	45.3	-27.5	-8.9
6,4'-DIHYDROXYFLAVONE	56.1	11.2	8.7	-11.6	50.5	23.3	-28.6	2.1
XANTHYLETIN	57.1	33.2	-1.3	-6.2	52.1	34.5	-60.9	-7.2
CANTHARIDIN	46.3	1.2	15.4	-11.9	41.5	28.9	14.6	-2.9
BUSSEIN	34.1	20.0	17.3	-4.0	31.8	27.5	2.1	-5.0
ERGOSTEROL ACETATE	36.3	-5.4	-0.3	-8.5	37.5	13.0	-9.5	-5.8
ALLOPREGNANOLONE	47.3	8.8	16.4	-9.1	56.7	42.6	-27.3	-7.7
SAPPANONE A DIMETHYL ETHER	39.6	-3.6	16.6	-6.9	38.5	12.1	-6.0	-8.6
TIGOGENIN	34.1	-0.7	12.5	10.7	43.8	27.5	-36.1	-6.5
DUARTIN, DIMETHYL ETHER	45.7	-8.1	11.1	-11.5	15.5	-16.5	9.4	-7.0
3-DEOXY-3beta-HYDROXYANGOLENSIC ACID METHYL ESTER	8.7	21.6	11.4	-4.6	10.2	6.5	-2.2	2.5
APOTOXICAROL	68.8	40.4	1.1	-13.3	48.6	38.2	-29.4	-8.7
beta-AMYRIN	69.9	48.6	9.1	-11.3	60.3	55.9	-36.6	-8.4
GIBBERELLIC ACID	48.0	17.3	4.9	-11.7	46.0	38.5	-17.9	-5.5
ORNITHINE	69.3	44.4	3.9	-13.5	47.2	46.2	-29.5	-8.4
ISOKOBUSONE	58.3	21.8	3.5	-11.1	54.3	26.9	-29.7	-7.0
CHRYSIN	60.4	42.2	-8.7	-13.2	45.0	33.7	-42.7	-8.0
ARBUTIN	43.0	2.0	16.9	-7.7	36.7	22.8	13.6	-7.3
DIOSMIN	51.3	31.0	7.5	-7.1	46.4	34.4	-10.5	-7.6
PELLETIERINE HYDROCHLORIDE	38.8	-15.3	18.2	-5.7	52.6	19.6	-28.9	-6.1
SALINOMYCIN, SODIUM	98.8	95.0	77.8	30.4	84.2	81.9	50.0	-7.9
2',2'-BISEPIGALLOCATECHIN DIGALLATE	54.6	13.2	6.7	-10.1	56.2	23.2	-24.4	-6.4
LITHOCHOLIC ACID	42.6	49.2	-23.9	-6.3	10.7	22.5	5.9	-5.6
HYPOXANTHINE	34.8	-24.0	12.3	-6.6	20.3	-9.2	13.4	-5.6
OROTIC ACID	4.5	27.4	10.3	-12.5	16.5	11.1	-1.5	-0.3
BIXIN	72.7	36.8	8.8	-12.0	43.7	35.8	-28.4	-9.0

STIGMASTEROL	67.8	48.6	-1.0	-11.5	53.2	49.4	-31.0	-7.9
TETRAHYDROSAPPANONE A TRIMETHYL ETHER	57.2	21.7	5.4	-16.1	44.8	34.0	-40.2	-9.1
STROPHANTHIDIN	64.9	38.0	3.0	-13.7	40.9	43.5	-11.1	-9.2
d,l-threo-3-HYDROXYASPARTIC ACID	61.2	23.2	-0.1	2.9	49.4	24.3	-37.9	-9.0
12a-HYDROXY-9-DEMETHYLMUNDUSERONE-8-CARBOXYLIC ACID	60.5	37.5	5.7	-6.5	43.3	34.8	-39.5	-8.3
USNIC ACID	55.6	18.3	15.8	-7.7	41.9	22.7	2.9	-7.4
THEAFLAVIN	39.9	19.7	17.6	-11.6	38.8	36.2	-6.5	-8.1
7,2'-DIHYDROXYFLAVONE	45.6	-4.2	0.5	-0.4	40.4	18.6	-39.0	-6.5
CHUKRASIN METHYL ETHER	43.8	-9.6	-2.3	-9.4	34.3	33.2	-61.0	-6.6
BETULIN	30.3	3.7	-6.7	1.8	40.4	17.1	-102.8	-7.3
GLUTATHIONE	42.8	15.3	5.6	-0.9	36.9	25.2	-38.4	-2.5
NORSTICTIC ACID PENTAACETATE	24.9	-13.8	18.6	11.9	22.9	-0.4	10.6	-2.6
DEACETYLGEDUNIN	47.8	44.6	9.6	-9.9	3.0	4.0	-3.2	-0.8
VIOLASTYRENE	54.3	20.1	13.7	-8.7	46.6	43.2	-28.0	-9.2
PTAEROXYLIN	54.1	40.3	10.3	-11.9	50.2	47.0	-37.3	-8.5
4,4'-DIMETHOXYDALBERGIONE	58.3	18.7	3.4	-12.6	46.6	39.3	-28.9	-5.6
UTILIN	50.8	23.3	25.4	-10.4	48.8	43.3	-12.3	-8.2
SITOSTERYL ACETATE	35.5	-10.8	6.7	-7.0	48.7	33.9	-31.4	-8.8
8-HYDROXYCARAPINIC ACID	48.2	21.5	20.2	-0.7	43.7	41.8	-17.7	-8.3
PIMPINELLIN	48.4	22.7	8.6	-9.0	35.2	27.4	16.5	-5.9
LUPEOL	36.5	3.7	22.9	-8.7	38.0	39.4	7.5	-4.6
3,7-EPOXYCARYOPHYLLAN-6-OL	41.2	-4.0	-0.6	17.7	48.4	25.8	-23.9	-5.4
BENZYL ISOTHIOCYANATE	37.3	-4.5	15.7	17.0	45.7	37.7	-9.4	-7.0
ISOSAFROLE	49.4	1.2	3.2	1.1	38.6	15.5	31.2	-7.2
HECOGENIN	34.1	6.9	28.0	11.8	43.2	27.3	5.0	-5.9
PHLORETIN	14.3	-21.3	8.3	6.9	5.3	-30.3	-55.5	-4.8
KASUGAMYCIN HYDROCHLORIDE	26.0	6.0	17.5	-9.1	-0.7	31.9	5.0	0.6
EPICATECHIN MONOGALLATE	30.2	0.4	14.6	-10.5	65.8	52.9	-50.7	-9.0
AESCULIN	56.5	39.0	11.9	-10.9	51.1	54.0	-24.6	-7.3
PHYTOL	44.9	12.1	22.4	-11.4	46.8	34.0	-24.1	-5.5
ANISODAMINE HYDROBROMIDE	56.3	32.4	13.2	-11.9	46.8	44.8	-19.8	-6.2
JUAREZIC ACID	44.1	20.1	-1.5	-11.2	46.8	28.2	-30.6	-8.0
LACTOBIONIC ACID	43.5	12.0	13.5	-11.1	52.4	46.1	-28.3	7.1
ACETOSYRINGONE	27.0	3.9	22.1	-9.3	38.1	33.4	10.0	-5.8
DIOSGENIN	23.7	-2.0	21.1	-7.1	37.9	41.8	10.9	-2.3
APIOLE	44.5	19.0	12.0	-4.1	43.9	32.4	-25.3	-6.3
ASARYLALDEHYDE	38.2	7.0	18.4	-11.5	54.7	43.5	-27.3	15.9
CEDROL	25.7	-2.4	15.9	2.9	36.4	21.6	-20.9	-5.5

HAEMATOXYLIN	33.2	2.8	13.6	-4.1	39.3	44.1	-1.1	-4.0
4-METHYLESCULETIN	10.1	-8.3	14.7	-5.1	19.0	-5.7	7.7	-5.4
ANGOLENSIN (R)	21.8	5.1	8.1	-11.4	13.7	12.8	4.5	-5.7
CARMINIC ACID	58.8	23.8	6.0	-12.1	48.3	46.1	-41.2	-8.1
BICUCULLINE (+)	48.9	34.7	8.9	-12.8	50.6	56.5	-38.6	-9.3
PIPERIC ACID	56.3	25.3	14.5	-11.8	46.9	39.4	-18.3	-6.9
BILIRUBIN	53.6	27.1	14.9	-13.6	44.0	45.6	-34.8	-7.6
ANDROSTA-1,4-DIEN-3,17-DIONE	49.9	16.2	13.9	1.2	52.9	37.5	-3.9	-8.2
KYNURENINE	52.4	22.6	8.3	-12.3	51.9	47.3	-50.7	-8.8
UMBELLIFERONE	38.9	-1.3	21.5	-11.2	39.2	35.5	6.3	-6.1
PRIMULETIN	33.1	4.9	15.6	-10.8	32.2	37.2	7.0	-7.4
GARDENIN B	49.7	17.8	9.9	0.5	51.1	27.4	-28.4	-6.9
DEOXYADENOSINE	33.0	5.5	16.5	-8.2	55.6	50.4	-5.2	-5.2
ONONETIN	35.1	17.8	21.2	6.7	38.4	20.8	-4.2	-5.4
RAUWOLSCINE HYDROCHLORIDE	30.1	7.5	13.9	-6.3	38.2	34.3	-26.6	-5.4
HAEMATOXYLIN PENTAACETATE	19.4	-3.1	22.1	-1.0	17.0	-2.4	16.0	-4.9
CONESSINE	26.9	10.4	12.7	-8.5	15.1	18.0	-0.6	10.0
ACETYL ISOGAMBOGIC ACID	74.5	43.9	26.8	-13.5	50.6	34.1	-16.1	-8.8
RESVERATROL 4'-METHYL ETHER	54.6	46.3	5.2	-9.0	51.6	53.2	-41.4	-6.5
CYTIDINE	42.8	11.1	17.3	-13.8	51.9	38.3	-33.8	-5.5
BETULINIC ACID	52.9	40.7	1.1	-10.5	34.7	50.7	-96.0	-9.1
beta-AMYRIN ACETATE	46.8	9.3	15.9	-12.1	57.0	38.2	-41.3	-8.3
URSINOIC ACID	54.0	28.9	12.2	-7.3	51.3	43.7	-29.4	-7.0
4-ACETOXYPHENOL	41.4	2.7	20.2	-9.7	41.5	17.2	8.9	-7.2
CHRYSANTHEMIC ACID	38.5	11.2	17.8	2.9	37.9	41.3	-30.8	-5.9
ATRANORIN	36.5	-4.3	16.9	-10.8	57.8	37.9	-36.5	-6.2
GOSSYPIN	47.3	19.3	14.0	-5.8	46.2	42.3	-26.8	-4.8
MYOSMINE	34.4	-5.9	26.0	-4.9	42.2	9.1	11.0	-5.9
HARPAGOSIDE	20.7	11.8	17.3	22.9	41.4	26.6	-4.3	-5.1
ESTRADIOL METHYL ETHER	18.7	-23.6	19.8	-8.2	22.1	-15.5	-26.5	-3.9
AVOCADYNE	21.5	-1.6	13.0	-9.5	14.1	9.2	-4.1	-7.8
ANTHRAQUINONE	66.0	35.2	-12.2	-12.0	56.2	47.7	-43.2	-7.2
SOLANESYL ACETATE	59.0	47.9	10.2	-14.5	39.6	48.9	-24.0	-10.1
PRIDINOL METHANESULFONATE	42.6	10.0	18.2	-11.1	44.0	30.9	-23.2	-7.4
HELICIN	55.9	36.1	17.8	-11.2	45.7	47.8	-23.7	-6.6
N-METHYL-D-ASPARTIC ACID (NMDA)	46.6	-0.1	15.9	-9.9	54.1	34.4	-34.8	-5.3
CARYLOPHYLLENE OXIDE	53.4	36.5	1.0	-14.2	50.9	47.5	-58.3	-6.9
LACCAIC ACID A	39.3	11.3	18.5	-12.2	33.7	29.8	12.1	-8.0
S-ISOCORYDINE (+)	38.3	11.1	17.4	-6.1	38.7	36.3	-0.9	-4.6
HYDROXYPROGESTERONE	30.9	-1.8	8.7	-11.4	55.7	29.2	-29.4	-5.7

ESCULETIN	47.0	25.9	10.3	-6.1	59.3	47.9	-18.9	-2.9
XANTHOXYLIN	40.9	7.1	24.5	0.6	46.0	17.3	13.3	-7.2
KINETIN RIBOSIDE	30.5	7.8	23.3	-5.7	51.2	38.6	0.4	7.2
beta-SITOSTEROL	16.3	-22.5	25.4	-3.5	5.1	-9.8	11.3	-3.7
SCOPOLETIN	-13.3	7.7	15.4	3.2	-3.7	13.2	-16.0	-0.1
ARABITOL(D)	55.2	29.2	7.4	-9.8	31.5	21.8	-30.5	-6.4
PARTHENOLIDE	71.5	66.1	1.6	-12.0	49.6	50.1	-37.6	-6.4
DIHYDROTANSHINONE I	70.2	66.4	-6.8	-10.2	51.1	43.0	-85.0	-6.0
CONVALLATOXIN	52.8	33.7	23.1	-13.4	46.5	47.1	-19.5	0.7
3,4-DIMETHOXYDALBERGIONE	56.3	27.3	7.4	-13.7	53.1	38.7	-32.8	-8.4
LAGOCHILIN	42.8	25.1	16.5	-12.9	50.5	48.5	-20.6	7.7
DIGITONIN	45.7	15.5	19.6	-5.3	28.3	12.1	11.1	-2.1
HIERACIN	26.6	5.0	17.1	-12.4	32.8	38.2	14.8	6.3
ISOROTENONE	43.9	-0.2	8.1	-7.9	58.4	36.0	-17.4	-4.7
RHETSININE	26.2	-1.9	13.4	-1.0	52.6	45.1	-30.7	10.4
CHOLESTANE	37.2	4.2	13.0	-2.1	45.1	26.2	-45.6	10.5
LAPPACONITINE	33.1	12.6	12.6	-1.9	35.6	31.0	-14.7	12.2
3alpha-ACETOXYDIHYDRODEOXYGEDUNIN	0.9	-11.0	15.4	-1.6	6.2	27.5	-32.8	-4.7
PLUMBAGIN	27.5	3.2	16.0	-8.4	2.8	17.1	6.1	-1.1
GLAFENINE	72.0	43.5	-6.6	-15.2	46.0	41.1	-41.3	-8.9
2',4'-DIHYDROXY-4-METHOXYCHALCONE	63.6	52.8	6.6	-12.0	47.2	55.2	-23.6	-6.5
TOLFENAMIC ACID	83.6	42.6	-0.3	-14.6	43.3	27.6	-63.0	-8.2
LUPANYL ACID HYDROCHLORIDE	54.9	45.5	21.4	-11.0	51.2	52.6	-16.4	-6.1
AMINOCYCLOPROPANECARBOXYLIC ACID	65.0	23.1	15.8	-9.9	47.3	24.0	-40.2	-6.3
INDOLE-3-CARBINOL	76.1	65.6	18.2	-12.4	47.3	45.0	-39.5	-5.7
BUCLADESINE	42.6	6.7	22.0	-10.2	31.6	15.9	-2.2	-7.6
RHIZOCARPIC ACID	37.5	13.3	24.5	-10.1	40.4	43.9	-13.1	-4.6
RETINYL ACETATE	41.9	19.6	11.8	-10.4	46.3	22.0	-34.9	-6.4
URSOCHOLANIC ACID	43.2	20.1	5.9	-9.1	54.8	48.1	-36.1	-6.2
STRYCHNINE METHIODIDE	31.3	5.7	17.0	-3.4	35.7	16.4	-20.4	-7.9
CORALYNE CHLORIDE	45.4	31.9	17.9	-6.8	40.0	38.3	-16.7	-6.6
LIMONIN	13.1	-11.8	19.5	-5.0	16.7	6.5	3.1	-4.6
CHOLIC ACID, METHYL ESTER	29.6	8.0	13.9	-10.3	-1.8	21.4	3.7	1.3
DEGUELIN(-)	63.3	53.6	21.6	-10.9	41.9	39.8	-32.1	-6.8
KARANJIN	62.2	51.3	8.1	-10.4	49.5	45.0	-21.1	-5.6
FORMONONETIN	43.0	28.9	12.4	-12.4	48.0	43.9	-37.6	-7.4
AMYGDALIN	39.9	31.9	23.2	-10.9	43.0	47.2	-9.8	-6.7
7,4'-DIMETHOXYISOFLAVONE	49.9	15.1	16.7	-9.9	49.7	29.1	-29.0	-5.6
AZADIRACHTIN	49.7	30.1	9.9	-12.9	49.2	51.2	-31.1	-6.5
KUHLMANNIN	33.0	8.8	21.8	-10.6	37.5	24.7	0.6	-6.2

CAFESTOL	31.2	19.7	14.7	-10.0	33.1	53.0	3.2	-5.7
HYDROQUINIDINE	46.1	13.2	15.8	-8.6	56.5	37.7	-15.9	-7.0
SALSOLINE	41.3	10.8	7.8	-9.9	41.8	42.6	-8.9	-5.5
LITHOCHOL-11-ENIC ACID	36.5	35.1	-10.3	-10.0	35.8	20.1	-5.7	-3.7
DIFFRACTAIC ACID	27.4	30.4	-8.6	-1.9	28.6	39.6	-79.0	-5.3
SOLASODINE	1.8	-8.5	22.4	-5.6	19.7	-3.0	-16.6	-6.9
ASARININ (-)	-12.9	29.6	-9.1	-11.7	-5.2	9.4	-65.9	-4.3
DIGOXIGENIN	42.0	15.3	8.6	-10.4	40.0	30.7	-34.1	-7.5
DIHYDROMYRISTICIN	42.0	41.1	7.2	-9.1	39.2	50.1	-21.5	-6.2
CAPERATIC ACID	41.7	15.7	18.9	-11.3	38.8	28.9	-5.3	-3.9
3,4-DIMETHOXYCINNAMIC ACID	48.8	37.6	26.6	-11.4	41.5	47.3	-17.0	-6.2
MEBHYDROLIN NAPHTHALENESULFONATE	45.2	8.8	13.3	-11.0	45.2	27.5	-17.5	-2.3
DESOXYPEGANINE HYDROCHLORIDE	30.0	7.9	16.7	-12.4	48.8	50.5	-103.4	-9.1
ESTRADIOL-3-SULFATE, SODIUM SALT	23.9	5.3	26.1	-10.4	38.2	24.9	17.6	-2.6
LUPANINE PERCHLORATE	30.4	22.0	23.3	-10.1	32.3	42.0	10.3	-6.5
CYCLOLEUCINE	38.2	10.4	18.0	-10.8	43.9	30.5	-17.6	3.5
SOLANESOL	40.8	12.5	15.5	-7.0	43.6	43.6	-13.9	-7.7
MORIN	24.9	1.1	22.4	-12.0	55.1	34.7	-32.3	6.9
ANDROSTERONE	32.9	9.6	16.7	4.3	22.4	31.7	-7.3	-3.6
PERICIAZINE	5.6	-11.5	17.1	-4.0	21.3	-16.4	-17.9	-5.2
CEPHALOSPORIN C SODIUM	-26.4	13.6	20.6	5.1	3.5	1.6	15.7	22.0
PANGAMIC ACID SODIUM	-5.0	-3.7	14.6	-9.9	39.5	37.1	-38.4	-7.9
BOLDINE	44.8	48.4	10.5	-9.7	54.7	57.4	-41.4	-6.1
GUAIOL(-)	39.7	18.6	2.5	-12.0	39.3	34.2	-27.8	-5.7
GINKGOLIDE A	38.8	37.6	19.0	-8.9	35.9	42.4	-26.3	-7.6
7,8-DIHYDROXYFLAVONE	56.0	27.4	20.1	-9.9	48.0	25.9	-72.0	-5.9
LINALOOL (+)	45.5	34.5	21.1	-9.6	48.7	45.2	-47.9	-7.2
CHRYSOPHANOL	30.1	-3.2	15.6	-11.5	39.5	25.2	-5.8	-7.0
HEMATEIN	12.5	17.8	14.0	1.0	55.8	55.6	-16.1	-5.8
IRIGINOL HEXAACEATATE	36.6	-1.8	13.5	-12.5	43.4	20.9	-52.9	-8.9
DESACETYLCOLFORSIN	40.3	15.1	9.9	-11.5	48.1	44.9	-39.3	-2.1
beta-TOXICAROL	36.9	31.6	17.2	-9.2	29.6	8.1	-21.9	9.7
TRIPTOPHENOLIDE	31.0	19.3	14.2	9.3	23.7	31.9	-6.4	9.0
OCTOPAMINE HYDROCHLORIDE	11.0	-8.3	18.3	-2.1	4.7	-12.3	-23.0	-7.3
RHOIFOLIN	-8.3	25.7	11.1	-9.1	5.0	35.3	-16.8	1.4
BROMO-3-HYDROXY-4-(SUCCIN-2-YL)-CARYOLANE gamma-LACTONE	45.5	14.8	-9.2	-10.3	19.7	14.0	-68.5	-7.0
10-HYDROXYCAMPTOTHECIN	51.3	41.2	9.3	-5.8	43.4	54.7	-40.4	-5.9
DICTAMNINE	28.5	-5.7	3.3	-9.2	32.6	15.5	-44.1	-1.2
TETRANDRINE	49.1	37.4	24.3	-8.7	38.8	47.3	-21.4	-6.5

IRIGENIN	53.4	9.1	10.1	-7.3	40.3	28.4	-38.0	-6.8
EUPHORBIASTEROID	16.4	15.1	2.9	32.2	-12.5	-26.4	-18.2	15.4
3-HYDROXY-4-(SUCCIN-2-YL)-CARYOLANE delta-LACTONE	45.2	-8.2	-18.7	-43.7	26.3	-1.0	-16.0	-13.1
2',4'-DIHYDROXYCHALCONE	37.6	-0.1	-10.9	-15.8	33.2	3.5	-20.8	-11.3
PERUVOSIDE	38.0	-20.1	-3.5	-46.4	28.5	-10.1	10.7	-6.4
2',3-DIHYDROXY-4,4',6'-TRIMETHOXYCHALCONE	38.0	-2.1	7.7	-23.0	40.6	25.8	-21.5	-3.6
6,7-DICHLORO-3-HYDROXY-2-QUINOXALINECARBOXYLIC ACID	35.7	-36.7	-4.8	-31.4	37.0	-27.8	12.9	6.9
THEANINE	41.8	-10.4	-1.7	-39.9	49.4	15.2	-28.2	-13.6
ISOGUVACINE HYDROCHLORIDE	30.1	-62.4	11.9	-2.8	39.1	-28.2	20.7	-5.9
GENETICIN	47.2	-1.8	-4.2	0.0	46.4	3.2	3.5	-5.2
D-PHENYLALANINE	33.2	-32.7	-11.0	10.4	42.7	-26.9	-17.0	-12.6
SEROTONIN HYDROCHLORIDE	28.8	-20.7	-11.3	4.6	49.7	2.4	-28.5	1.1
ANISOMYCIN	43.7	-56.8	-10.0	-43.0	54.3	-11.3	-6.6	-18.9
CARYOPHYLLENE [t(-)]	16.8	-19.2	0.2	4.6	35.3	2.7	-39.2	-16.4
1-HYDROXY-3,6,7-TRIMETHOXY-2,8-DIPRENYLXANTHONE	29.1	-65.6	15.0	15.2	38.3	-36.5	5.2	-8.3
HARMALINE	34.0	8.9	6.5	-32.1	0.1	-0.7	-28.1	-5.7
MENTHYL BENZOATE	44.9	-16.0	-6.5	-28.0	40.6	4.2	-18.9	-12.7
GITOXIN	55.7	21.4	-6.1	-39.5	49.1	28.9	-42.8	-11.8
MANDELIC ACID, METHYL ESTER	52.5	-12.8	1.4	-44.4	48.1	19.9	-6.3	-13.2
AZASERINE	59.4	28.3	2.8	-39.6	39.1	19.0	-1.1	-12.1
5,7-DIHYDROXYISOFLAVONE	68.4	4.1	-5.7	-46.0	45.5	2.5	-24.7	1.8
FISETIN	38.2	-6.7	1.9	-38.8	41.3	8.7	-26.0	-12.4
2-METHOXYRESORCINOL	48.4	-35.1	8.7	-23.3	32.0	-7.1	28.6	-7.5
CEVADINE	44.4	-14.8	5.8	-20.2	31.7	0.9	1.1	-6.9
6-HYDROXYANGOLENSIC ACID METHYL ESTER	57.6	-24.8	-5.2	-7.5	33.1	-13.7	-16.8	9.7
UBIDECARENEONE	46.8	-16.0	-10.7	-25.2	37.7	3.9	-31.8	-11.0
STIGMASTA-4,22-DIEN-3-ONE	49.7	-18.6	-1.6	-33.1	47.1	-0.3	-28.2	5.4
THERMOPSINE PERCHLORATE	40.9	-11.2	0.6	-20.6	20.2	1.7	-16.4	-7.9
FARNESOL	40.0	-59.5	3.8	-36.8	9.9	-59.3	20.6	-1.1
LINAMARIN	30.0	2.4	5.3	-22.9	14.6	-7.9	-5.9	-1.5
ROTENONE	60.8	19.0	-4.4	-19.6	22.6	2.2	-24.3	-14.6
ISOLIQUIRITIGENIN	63.0	36.4	-9.1	-44.0	32.4	16.3	-21.9	-13.5
APIGENIN DIMETHYL ETHER	58.2	25.8	-13.4	-43.8	43.5	16.6	-56.2	-14.7
ASTRAGALOSIDE IV	55.1	8.7	0.6	-42.2	26.1	10.4	-11.8	-10.6
OXONITINE	56.2	18.0	-4.2	-46.0	53.3	5.2	-23.0	-13.4

MANGIFERIN	58.2	4.5	-2.7	-45.1	52.4	19.1	-15.0	-15.9
PROTOVERATRINE A	44.6	-13.4	10.3	-29.8	28.9	3.0	11.7	0.6
GARLICIN	50.7	-17.1	5.7	-35.2	35.9	11.4	7.3	-17.3
4-NAPHTHALIMIDOBUTYRIC ACID	49.5	-5.9	-13.1	-42.7	61.3	17.7	-10.7	-13.8
HUPERZINE A	51.1	-10.0	-9.7	-44.4	53.2	19.3	-39.0	-8.9
p-CHLOROPHENYLALANINE	50.4	19.9	6.0	-34.5	57.7	18.3	-11.8	-16.4
SECURININE	42.2	-22.5	11.5	-39.3	30.1	8.0	-24.1	-13.4
EXALAMIDE	36.8	-38.9	-3.2	-34.7	-2.7	-27.5	9.7	2.1
SYRINGIC ACID	28.0	3.7	14.2	-15.5	16.8	-13.2	-7.6	-5.6
CACODYLIC ACID	55.6	17.1	-13.0	-24.7	36.8	22.5	-17.7	-14.0
CHOLEST-5-EN-3-ONE	65.1	27.6	-14.8	-37.5	45.6	22.4	-9.0	-16.7
MANGOSTIN TRIMETHYL ETHER	63.3	20.1	1.8	-23.3	36.8	7.1	-23.9	-13.8
HARMANE	62.5	24.2	-13.6	-37.0	37.4	21.5	-17.1	-15.0
1R,2S-PHENYLPROPYLAMINE	58.1	13.6	-2.1	-31.2	46.8	12.8	-22.0	-16.8
18alpha-GLYCYRRHETINIC ACID	62.1	19.6	-7.0	-45.2	40.6	9.7	-27.7	-8.5
CITRININ	49.9	-13.9	11.1	-20.1	30.8	7.6	12.4	-14.2
CHLORPYRIFOS	50.7	-4.3	14.1	-35.2	33.6	8.9	6.0	-6.9
CINEOLE	55.8	4.6	-10.3	-35.9	44.4	8.2	-25.2	-14.4
MONOCROTALINE	48.2	-6.4	-13.0	-46.2	44.4	11.8	-43.3	-15.0
KOBUSONE	57.6	7.8	3.4	-36.6	45.9	2.8	-33.2	-16.3
TRYPTAMINE	46.9	-12.0	17.9	-22.7	36.3	11.8	-19.2	-16.5
LIGUSTILIDE	26.6	-43.3	6.0	-24.4	-11.3	-16.4	-10.3	3.6
alpha-MANGOSTIN	12.9	14.2	15.8	-20.0	8.6	-3.9	-30.9	-12.7
LATHOSTEROL	59.2	9.1	-4.1	-31.2	36.5	15.1	-25.4	-11.0
FUCOSTANOL	77.4	49.0	-21.1	-45.1	56.1	42.1	9.6	-13.2
NEROL	64.5	16.0	4.0	-41.7	38.6	16.3	-16.6	-12.1
3,7-DIHYDROXYFLAVONE	74.5	45.2	-15.0	-47.9	45.2	22.9	-19.7	-13.6
GAMBOGIC ACID	76.5	6.8	34.0	-43.0	34.3	-7.8	-11.6	-17.4
CANTHAXANTHIN (euglenanone)	76.8	36.9	-5.1	-46.9	46.4	20.4	-23.8	-15.4
5beta-12-METHOXY-4,4-BISNOR-8,11,13-PODOCARPATRIEN-3-ONE	44.1	-13.3	10.2	-32.0	34.7	10.1	-26.2	-14.8
DEMETHYLNOBILETIN	69.0	11.8	3.2	-45.4	38.7	15.5	7.7	-5.6
ARTHONIOIC ACID	47.5	-18.2	-11.2	-38.7	35.0	-9.7	-39.4	-13.7
PIPLARTINE	57.7	6.5	-17.0	-43.6	36.5	15.3	-32.3	-3.8
3-AMINOPROPANESULPHONIC ACID	63.0	-0.9	10.7	-24.1	42.7	-4.0	-20.3	-14.3
ASIATIC ACID	54.8	-3.0	-6.9	-44.0	41.9	22.3	-93.2	-12.1
IMIDAZOL-4-YLACETIC ACID SODIUM SALT	39.7	-28.4	24.1	-21.1	6.5	-30.5	-3.3	7.5
ICARIIN	34.0	2.0	11.4	-22.2	18.3	-11.1	-8.4	-3.9
ABIETIC ACID	60.3	23.0	-14.3	-45.9	17.6	14.6	-21.4	-14.8
CURCUMIN	58.7	32.3	-3.2	-37.4	45.1	42.8	-27.5	-14.3

HEXAMETHONIUM BROMIDE	52.1	8.6	-12.6	-38.7	35.5	14.2	-13.9	-12.8
AGARIC ACID	61.0	30.1	6.0	-41.7	50.2	33.6	-17.8	-14.7
THIOCTIC ACID	60.2	4.9	-6.1	-43.8	46.4	17.7	-25.9	-16.6
CHOLEST-4,6-DIEN-3-ONE	58.0	14.7	-3.8	-41.3	43.8	20.3	-13.3	-13.6
KAEMPFEROL	49.1	1.3	4.6	-40.5	20.7	5.1	-46.4	-4.1
MIMOSINE	45.6	-14.5	7.8	-37.5	30.3	5.7	-15.0	-10.3
KAINIC ACID	55.5	-6.0	-13.0	-45.8	47.0	13.3	-33.1	-7.5
3-HYDROXYFLAVONE	59.0	-2.9	-25.5	-45.0	54.3	4.9	-53.0	-17.5
ACTINONIN	79.9	38.4	24.4	-26.1	47.7	6.6	-52.8	-12.5
RESVERATROL	48.1	-21.8	3.8	-38.2	36.7	15.0	-31.4	-9.8
HUMULENE (alpha)	38.8	-20.6	2.9	-34.4	19.5	-24.1	-33.5	-8.3
QUEBRACHITOL	74.2	45.6	-1.2	-41.3	37.6	27.3	-11.2	-16.6
RETUSIN 7-METHYL ETHER	23.0	-9.1	5.4	-29.4	0.6	13.4	-39.1	-10.2
BATYL ALCOHOL	84.0	59.9	-10.6	-43.4	39.5	14.7	-36.1	-17.5
ANDROGRAPHOLIDE	72.3	35.4	-4.9	-44.6	49.6	37.4	-17.2	-16.1
HEDERAGENIN	70.9	39.5	-6.0	-45.5	32.7	26.3	-66.8	-14.3
TRIGONELLINE	72.4	39.0	-7.4	-48.1	44.6	19.0	-41.3	-18.3
TOMATINE	54.7	-12.8	-4.4	-45.3	41.8	23.0	-33.6	-12.6
ROSMARINIC ACID	74.2	23.3	2.1	-45.5	36.7	6.0	-0.2	11.5
6,3'-DIMETHOXYFLAVONE	41.6	-14.1	3.1	-42.1	25.7	0.1	-52.2	-14.8
ORSELLINIC ACID, ETHYL ESTER	57.1	3.9	-5.9	-38.9	40.1	3.6	-28.4	0.8
18-AMINOABIETA-8,11,13-TRIENE SULFATE	55.1	-0.4	-2.9	-45.0	40.7	19.3	-11.2	11.4
2,3,4-TRIHYDROXY-4'-ETHOXYBENZOPHENONE	65.8	19.0	15.6	-38.4	45.7	11.5	-25.3	-11.8
BACCATIN III	58.6	-13.1	6.8	-39.5	34.7	20.2	-13.7	-1.6
ADONITOL	53.5	-10.3	-19.8	-38.3	18.8	-17.8	-1.1	8.8
2',4-DIHYDROXY-3,4',6'-TRIMETHOXYCHALCONE	28.1	-6.5	10.2	-21.9	12.6	-4.9	-24.7	-11.7
SULFAPHENAZOLE	78.5	44.5	5.5	-46.4	34.3	26.0	-24.0	-1.9
AURAPTENE	66.1	49.4	-16.2	-45.0	34.3	14.1	-14.0	-15.9
N- (9-FLUORENYLMETHOXYCARBONYL)-L-LEUCINE	59.7	25.7	-3.6	-44.4	36.9	25.6	-10.3	-6.8
NOBILETIN	66.9	28.5	8.3	-44.9	47.9	26.3	-7.2	-11.6
PHENETHYL CAFFEATE (CAPE)	50.6	0.5	4.9	-47.3	37.8	10.3	-23.5	-12.5
3,4',5,6,7-PENTAMETHOXYFLAVONE	60.6	3.0	13.6	-43.4	51.7	17.4	-20.9	-5.8
ZOPICLONE	52.2	-7.5	17.4	-43.0	29.8	4.3	12.3	4.9
SKATOLE	52.3	-9.8	22.0	-41.8	34.5	4.4	-9.9	-13.0
5-METHYLFURMETHIDE	60.4	-2.6	-4.5	-44.2	44.7	13.9	-10.1	-13.6
7-OXOCHOLESTEROL	51.5	-11.1	4.8	-26.1	40.5	19.7	-22.6	-16.1
MEVALONIC ACID LACTONE	60.7	-3.9	13.5	-44.5	28.1	4.9	8.0	-15.8

NORHARMAN	51.0	-16.9	6.2	-43.3	40.1	13.8	-32.0	-12.1
LOBARIC ACID	31.9	-21.4	4.9	-33.9	-14.8	-18.1	-4.2	-11.3
BERBAMINE HYDROCHLORIDE	21.4	-8.8	15.5	0.3	11.3	5.7	-17.3	-0.5
DIMETHYLCAFFEIC ACID	71.1	31.1	4.6	-27.8	36.5	11.8	12.7	-9.3
CELLOBIOSE (D[+])	60.1	23.5	-8.2	-42.1	38.9	34.5	-38.6	-15.7
YOHIMBIC ACID HYDRATE	62.9	16.4	9.7	-44.9	44.6	21.2	-19.0	5.3
URIDINE TRIPHOSPHATE TRISODIUM	46.5	-0.5	16.6	-47.3	41.2	32.5	-3.2	-14.8
ROBUSTIC ACID	78.1	53.0	-25.1	-42.8	27.5	-4.8	-30.4	-12.7
CAFESTOL ACETATE	49.0	-10.1	12.4	-44.5	41.4	22.3	-30.3	-14.7
TRIDESACETOXYKHIVORIN	44.8	-19.6	18.9	-39.2	30.0	11.9	-22.5	-12.1
HEDERACOSIDE C	43.3	-19.1	24.7	-39.0	31.0	8.7	1.1	-14.0
ARTEMISIN	50.8	-10.8	4.4	-29.5	48.3	9.9	-32.6	-4.1
DIOSMETIN	30.8	0.7	1.7	-47.3	32.9	4.2	-23.5	-16.7
BUFEXAMAC	59.4	-1.6	7.6	-39.4	33.8	19.6	-9.9	-12.1
LEUCODIN	48.1	-17.3	6.7	-38.5	34.9	20.8	-21.9	-13.0
PYRROMYCIN	45.7	-45.6	20.8	-13.2	9.6	-29.6	15.4	-10.8
PICROPODOPHYLLIN	23.2	-9.6	18.3	-5.5	9.7	-2.4	3.2	-6.6
THYMOQUINONE	68.1	30.4	1.7	-43.1	39.3	20.1	-33.7	7.5
SALVINORIN A	63.8	33.8	-0.9	-33.0	48.5	40.2	-30.1	-15.2
2-METHOXYXANTHONE	53.3	2.2	12.4	-45.1	39.6	5.8	-29.1	-16.2
2',4-DIHYDROXYCHALCONE	54.8	19.8	7.3	-40.4	37.8	27.5	-18.7	-15.5
SUXIBUZONE	58.4	15.9	-3.9	-46.6	47.5	16.2	-40.6	-9.4
L(+/-)-ALLIIN	58.4	7.7	-3.4	-43.6	37.0	23.2	-26.9	-16.9
alpha-CYANO-3-HYDROXYCINNAMIC ACID	50.5	-7.4	22.5	-35.8	35.3	-2.4	12.4	-7.7
TANGERITIN	45.7	-23.4	22.4	-34.9	40.5	13.9	0.2	-15.6
L-LEUCYL-L-ALANINE	52.4	-10.2	1.8	-40.8	36.7	7.0	-18.3	-1.8
SINENSETIN	53.5	-2.9	-0.9	-40.4	52.2	30.8	-16.0	-14.6
METITEPINE MALEATE	53.6	2.2	9.1	-43.6	34.1	13.4	-16.7	-15.2
MELIBIOSE	41.5	-8.7	10.0	-29.2	28.2	13.4	-9.6	-13.6
LEVULINIC ACID, 3-BENZYLIDENYL-	51.7	-8.4	11.5	-36.3	30.6	-9.1	-33.3	-5.5
CITRULLINE	31.6	-5.1	10.0	-34.6	8.4	-10.2	-3.6	9.3
N-ACETYLMURAMIC ACID	60.7	23.7	0.9	-44.0	38.3	36.8	-27.2	-14.7
CORYNANTHINE	63.7	38.2	-10.2	-43.6	42.2	38.5	-29.5	-16.8
HOMOPTEROCARPIN	50.0	2.3	11.6	-39.6	43.8	21.8	-9.1	-15.7
PICROTOXININ	47.6	7.5	21.8	-37.9	37.4	26.6	-11.8	-12.4
EUPHOL	43.3	9.7	-0.3	-42.0	30.6	0.8	-19.3	-14.7
ASTAXANTHIN	45.6	-8.6	15.6	-42.2	40.6	25.9	-26.5	-13.8
12a-HYDROXY-5-DEOXYDEHYDROMUNDUSERONE	38.0	-11.7	13.4	-42.5	38.9	10.0	-6.3	-14.1
ALOIN	47.2	0.8	13.1	-41.8	32.4	15.6	4.8	-1.1

PSEUDO-ANISATIN	54.2	-14.9	-2.7	-36.9	45.2	15.7	-25.0	-11.8
CAFFEIC ACID	59.1	-6.3	-1.1	-40.0	42.5	20.8	-27.1	-14.7
DIHYDROFISSINOLIDE	60.6	1.9	-0.1	-35.5	39.6	13.4	-27.1	-12.0
EVOXINE	45.4	-10.6	7.5	-35.2	31.2	18.8	-31.9	-12.7
ANTIMYCIN A (A1 shown)	32.0	-40.7	17.1	-9.6	23.7	2.5	-22.0	-6.1
TRIACETYLRESVERATROL	25.2	-6.2	10.2	-11.9	9.5	-0.8	-9.9	2.5
HECOGENIN ACETATE	51.7	12.3	-2.0	-39.4	37.8	19.5	-17.5	-17.2
APHYLLIC ACID	63.1	30.8	0.0	-42.5	49.8	39.1	-13.6	-13.6
OBLIQUIN	40.7	-0.7	2.1	-45.2	32.4	11.3	-19.0	-14.5
7-HYDROXYFLAVONE	45.0	1.8	-3.1	-44.9	26.9	16.9	-7.0	-11.9
SMILAGENIN	52.8	3.0	1.7	-40.9	40.5	12.6	-2.7	-15.5
11a-ACETOXYPROGESTERONE	48.0	3.3	-14.1	-43.5	32.0	20.6	-6.4	-13.1
ALANYL-dl-LEUCINE	32.4	-25.9	20.4	-38.6	28.1	-1.2	6.4	-9.6
2',5'-DIHYDROXY-4-METHOXYCHALCONE	36.7	-8.3	22.2	-36.7	33.5	10.6	-2.1	-9.1
AZOBENZENE	38.8	-22.7	4.5	-35.8	40.0	4.1	-10.0	-13.3
EPITESTOSTERONE	58.6	-1.9	1.2	-33.7	39.3	18.1	-2.9	-9.4
5-FLUOROINDOLE-2-CARBOXYLIC ACID	54.1	6.3	-3.9	-34.3	15.1	5.5	-34.2	-12.6
3-HYDROXY-3',4'-DIMETHOXYFLAVONE	37.9	-15.1	12.0	-28.3	23.1	16.9	-11.4	-12.3
6alpha-METHYLPREDNISOLONE ACETATE	34.8	-39.7	6.8	-34.8	12.9	-13.8	-6.9	-11.9
5-HYDROXY-2',4',7,8-TETRAMETHOXYFLAVONE	2.1	-13.7	11.6	0.7	16.2	-7.8	0.1	5.7
1-PHENYLBIGUANIDE HYDROCHLORIDE	50.2	2.5	6.7	-36.7	37.2	22.6	-27.2	-12.3
ALEURETIC ACID	60.1	27.9	4.8	-38.1	32.9	29.3	-11.2	-14.4
METHOXYAMINE HYDROCHLORIDE	46.5	-4.4	14.4	-43.0	41.5	22.0	-16.3	-13.6
CHOL-11-ENIC ACID	36.8	4.1	10.7	-46.5	21.2	12.4	-10.7	-15.4
KAWAIN	52.6	3.3	1.4	-34.4	47.3	22.0	-25.2	-13.9
KYNURAMINE	45.7	-13.5	18.7	-47.5	37.8	17.8	-17.0	-7.9
4-METHOXYDALBERGIONE	51.7	-5.2	18.4	-41.4	27.0	2.1	5.5	-5.9
CYTISINE	39.5	-10.1	29.0	-34.2	37.5	18.7	-0.9	-11.3
THEAFLAVIN DIGALLATE	53.6	2.7	0.1	12.5	63.8	30.9	-68.2	-12.9
HARMOL HYDROCHLORIDE	54.6	-13.1	0.3	-36.9	38.0	18.6	-15.4	-9.5
PYROCATECHUIC ACID	49.3	3.1	16.0	-41.3	29.5	8.4	-8.6	5.2
SAFROLE	45.7	-11.1	13.1	-37.5	28.4	17.5	-23.2	-13.0
4'-METHOXYFLAVONE	41.8	-28.6	7.3	-34.9	-9.2	-56.4	-24.0	-2.2
GLUCITOL-4-GUCOPYANOSIDE	21.1	-12.8	8.5	-39.6	5.1	9.1	-24.2	-1.0
alpha-TOXICAROL	47.2	38.8	6.1	-40.9	43.5	23.6	-55.3	-17.7
SALSOLIDINE	60.8	46.1	0.1	-40.0	39.6	35.1	-30.8	-15.2
N-ACETYLPROLINE	48.4	-9.1	11.0	-44.1	38.4	20.5	-26.6	-10.4
MYRICETIN	51.8	11.6	7.2	-50.1	36.0	29.8	-8.7	-14.2
PERILLIC ACID (-)	49.9	9.1	-7.5	-45.5	40.5	19.4	-41.7	-15.4

ANABASAMINE HYDROCHLORIDE	54.0	7.9	-14.0	-47.1	39.0	21.6	-19.9	-15.7
HAEMATOPORPHYRIN	36.1	-26.3	-9.8	-46.6	28.9	5.8	-13.7	-13.9
GYROMITRIN	52.1	1.7	12.9	-42.5	39.7	29.3	5.1	-9.5
4'-HYDROXYCHALCONE	34.0	-25.9	9.1	-43.3	36.6	-0.5	-39.2	-14.0
21-ACETOXYPREGNENOLONE	57.2	-4.3	2.6	-36.4	43.7	25.6	-31.4	-9.7
HARMALOL HYDROCHLORIDE	45.6	-21.3	18.7	-28.6	40.4	5.4	-20.8	-15.1
GOSSYPETIN	55.7	-6.9	17.1	-20.0	37.0	24.1	-32.2	-14.4
TODRALAZINE HYDROCHLORIDE	39.5	-24.3	19.0	-25.6	8.7	-18.9	-22.3	-11.3
RHODOCLADONIC ACID	16.9	-0.5	16.3	-0.1	16.7	-2.4	-32.0	-13.2
p-FLUOROPHENYLALANINE	49.0	8.5	15.6	-32.9	39.6	18.5	-33.8	-14.6
PERILLYL ALCOHOL	54.2	41.1	2.3	-40.8	32.8	40.0	-4.2	-18.1
DEQUALINIUM CHLORIDE	75.2	52.2	70.6	2.3	39.9	13.5	-1.4	-16.4
HEXAMETHYLQUERCETAGETIN	50.0	1.0	9.3	-40.6	47.9	38.7	-9.4	-13.1
SODIUM THIOGLYCOLATE	54.2	5.5	6.6	-43.7	28.9	-8.2	-6.6	-12.2
alpha-ERGOCRYPTINE	42.3	1.4	1.3	-39.8	42.3	22.4	-19.3	-15.5
TULOBUTEROL	35.9	-16.5	11.2	-13.8	24.7	-3.6	3.4	-12.5
TRIMEDLURE	37.0	-11.5	11.1	-20.2	32.9	18.6	7.3	-10.7
2-MERCAPTOBENZOTHIAZOLE	46.0	-24.0	3.7	-29.7	38.8	0.5	-2.0	-7.3
TANSHINONE IIA SULFONATE SODIUM	57.9	4.6	-3.0	-30.2	56.5	27.6	-48.4	-11.5
3-AMINO-1,2,4-TRIAZOLE	52.6	-11.9	14.0	-36.9	31.2	4.5	-18.2	-6.1
4'-DEMETHYLEPIPODOPHYLLOTOXIN	59.6	-1.3	13.1	-28.2	45.7	10.4	-16.8	-12.7
PROXYPHYLLINE	35.6	-38.0	27.8	4.1	9.9	-16.9	-2.6	9.5
METHIONINE SULFOXIMINE (L)	16.7	10.7	12.8	6.7	13.2	-2.5	-5.4	9.8
DIHYDROFOLIC ACID	61.8	27.3	2.3	-44.6	34.3	15.0	-17.5	-11.6
ARTENIMOL	50.2	35.1	4.1	-42.3	32.8	36.2	-22.3	-5.2
CRUSTECDYSONE	48.8	-9.1	14.4	-43.2	43.5	25.8	-13.3	-13.6
2,5-DI-t-BUTYL-4-HYDROXYANISOLE	59.4	22.6	8.8	-47.6	44.5	38.2	-14.7	-12.9
MADECASSIC ACID	59.2	18.6	-6.9	-48.2	52.8	19.8	-78.6	-16.5
L-PHENYLALANINOL	53.0	10.4	10.8	-46.8	45.1	24.2	-20.1	-12.1
CAMYLOFINE DIHYDROCHLORIDE	37.8	-16.3	26.3	-41.6	25.0	0.8	8.1	-12.1
SALSOLINOL HYDROBROMIDE	51.2	-6.8	24.1	-45.7	32.7	14.2	0.6	-14.4
ACETYLGLUTAMIC ACID	42.5	-18.0	9.1	-19.1	39.4	1.3	-40.1	-4.5
GLICLAZIDE	44.8	0.5	11.5	-44.0	43.9	20.5	-19.4	-11.1
N,N-HEXAMETHYLENEAMILORIDE	53.5	-8.4	11.0	-30.3	36.5	11.9	-29.5	-2.3
VALINOMYCIN	44.4	-14.1	16.6	-37.3	36.8	19.8	-14.5	-10.4
AURIN TRICARBOXYLIC ACID	20.6	-22.2	-26.8	-25.0	-0.1	-10.5	-54.4	-13.2
VALERYL SALYCILATE	-3.1	-22.7	6.9	-19.4	10.6	-11.3	-0.7	16.6
ETHIONINE	61.6	21.9	7.6	-48.6	36.0	23.2	-40.5	-1.6
TRIMETHYLCOLCHICINIC ACID	70.0	47.1	-6.4	-46.2	39.2	38.5	-27.2	-5.5
ETHOXYQUIN	52.6	-15.8	4.2	-47.7	45.3	17.6	-16.3	-16.2

ANCITABINE HYDROCHLORIDE	41.5	5.7	25.2	-41.2	41.1	31.3	-20.5	-13.1
FENBUTYRAMIDE	58.6	-2.7	5.8	-43.7	40.6	8.2	-7.4	-11.3
BISPHENOL A	56.4	15.6	2.4	-40.8	34.9	16.7	-28.3	-12.3
ACADESINE	40.3	-18.3	15.5	-44.7	24.6	4.8	0.4	-2.0
3,3'-DIINDOLYLMETHANE	30.9	-1.2	29.8	-35.1	28.1	18.2	-11.8	-9.3
11alpha-HYDROXYPROGESTERONE HEMISUCCINATE	50.0	-14.2	10.8	-44.0	36.1	13.3	-23.8	-7.9
THIOGUANOSINE	47.8	14.2	-1.4	-44.0	35.6	17.9	-25.5	-13.1
O-BENZYL-l-SERINE	50.4	-9.7	23.4	-42.9	36.8	10.8	-27.2	-15.8
CHOLIC ACID	43.4	-11.1	19.4	-30.9	24.1	18.7	-66.7	-14.5
2,3-DIMERCAPTOSUCCINIC ACID	33.9	-40.7	28.3	-32.6	12.8	-22.3	-5.4	-9.4
4-(3-BUTOXY-4-METHOXYBENZYL)IMIDAZOLIDIN-2-ONE	23.1	3.1	14.6	-26.7	16.9	-7.6	-18.5	1.8
2-METHYL-4-(PIPERIDIN-1-YLCARBOXY)-5-ISOPROPYLPHENYLTRIMETHYLAMMONIUM CHLORIDE	51.6	16.1	8.3	-33.0	35.9	23.3	-34.9	-15.9
ELAIDYLPHOSPHOCHOLINE	60.4	37.0	8.7	-43.0	44.8	40.7	-5.9	-14.0
DIFLORASONE DIACETATE	45.5	-5.1	24.5	-47.9	42.7	25.4	-21.3	-15.0
MOROXYDINE HYDROCHLORIDE	55.4	21.4	15.6	-44.8	52.7	33.7	-2.7	-11.5
ACETYLGLUCOSAMINE	47.7	-0.9	14.9	-43.6	43.3	13.5	-23.9	-11.7
3-METHYLXANTHINE	53.5	10.1	-22.1	-45.5	63.7	42.3	1.5	-13.2
PUERARIN	34.9	-7.9	33.6	-40.5	28.4	5.7	13.1	-15.5
MANGANESE TETRAKIS(4-CARBOXYPHENYL)PORPHYRIN CHLORDE	46.7	5.4	22.8	-39.4	28.9	15.6	5.7	-11.4
4,4'-DIISOTHIOCYANOSTILBENE-2,2'-SUFONIC ACID SODIUM SALT	37.5	-26.6	2.7	-43.8	44.0	21.2	-36.6	-12.5
7-NITROINDAZOLE	50.1	-5.2	3.1	-39.1	54.7	30.8	-1.7	-9.6
NIALAMIDE	50.3	-19.3	20.8	-24.6	36.6	16.0	-40.1	-13.0
TRIMETAZIDINE DIHYDROCHLORIDE	44.6	-0.6	17.1	-35.8	46.0	14.5	-15.8	-9.0
ACETYLPHENYLALANINE	34.1	-33.6	21.7	-25.5	24.7	-11.1	-19.5	-12.4
NIMUSTINE	34.5	6.2	5.0	-42.8	2.1	15.1	-16.3	19.0
BETAMIPRON	60.8	9.1	-8.4	-41.3	53.9	24.9	13.5	-14.2
PRASTERONE	65.5	50.8	14.1	-38.9	42.8	44.0	0.8	-6.2
3-ISOBUTYL-1-METHYLXANTHINE (IBMX)	49.3	-1.9	8.5	-45.3	47.5	17.1	4.0	-13.5
SINOMENINE	49.6	14.4	17.6	-35.7	44.9	47.4	-2.1	-9.0
AMPYRONE	53.0	-2.8	-5.3	-43.3	40.8	11.8	-30.7	-8.1
DIETHYLTOLUAMIDE	58.4	14.9	7.8	-47.5	47.2	39.9	-0.8	-11.8
5,7-DIHYDROXY-4-METHYLCOUMARIN	39.2	-10.5	18.4	-39.9	30.5	-0.5	-0.7	-13.5
NORCANTHARIDIN	40.9	-4.6	20.2	-43.9	29.1	25.1	-2.4	-4.8

2,3-DIHYDROXY-6,7-DICHLOROQUINOXALINE	43.0	-15.1	12.6	-44.7	46.0	17.3	-28.4	-0.7
2,3-DICHLORO-5,8-DIHYDROXYNAPTHOQUINONE	49.6	-1.7	6.8	-45.5	45.4	28.0	-46.4	-7.1
BISSALICYL FUMARATE	53.8	-0.5	12.2	-42.2	38.8	11.2	-35.5	-13.7
PALMATINE	44.1	-9.6	16.1	-27.8	44.4	19.8	5.5	12.5
ASCORBYL PALMITATE	23.2	-31.3	22.0	-34.0	16.2	-30.5	15.9	4.5
METHOXYVONE	-0.4	-5.3	8.4	-35.0	0.1	0.2	1.8	3.5
ALLOXAN	45.6	-3.2	-4.8	-46.4	36.2	17.9	-37.3	-12.4
DIPLOSALSALATE	58.6	39.1	1.8	-41.7	44.8	42.1	-28.9	-13.9
3H-1,2-DITHIOLE-3-THIONE	40.6	-15.1	16.6	-45.7	32.9	22.4	-24.3	-10.1
2,2'-AZO-bis-2-AMINOPROPANE	49.4	27.1	13.0	-23.3	41.9	41.9	-31.2	-12.6
PROTIONAMIDE	48.6	-9.1	4.4	-40.9	63.7	42.5	0.3	-12.6
BICUCULLINE(-) METHIODIDE	57.9	18.3	7.7	-42.3	37.5	30.0	-30.2	-6.1
FAMPROFAZONE	67.7	34.1	57.4	8.8	32.3	-5.4	-11.5	-4.3
FLOPROPIONE	34.6	-17.4	22.5	-20.1	31.3	15.3	-9.2	-7.8
SODIUM DEOXYCHOLATE	75.4	35.5	40.0	-41.7	34.0	18.4	-130.2	-16.5
PHENYLBUTYRATE SODIUM	52.0	-23.1	6.2	-25.0	44.2	26.0	-43.0	-13.8
TRIMEBUTINE MALEATE	46.3	-13.6	16.7	-39.1	34.7	18.1	-49.2	-8.6
N-PHENYLANTHRANILIC ACID	50.3	-9.2	7.4	-24.1	20.9	14.8	-17.1	-11.7
CARMOFUR	99.1	93.6	89.6	88.6	64.8	20.4	20.5	1.4
SR-2640	26.6	0.3	6.5	1.3	-15.2	33.5	-80.1	-8.1
OXELAIDIN CITRATE	35.5	-5.4	-0.6	-41.0	31.7	7.4	-47.8	-9.5
ACEMETACIN	63.6	52.3	-9.8	-30.8	49.8	46.8	-24.9	-8.8
TOLPERISONE HYDROCHLORIDE	38.2	-1.4	13.2	-39.3	34.5	27.7	-37.5	-7.3

[1] Results are expressed as the average percentage of inhibition from two biological replicates.
