# Supplementary figures and images for: Optimization of a High-Throughput 384-Well Plate-Based Screening Platform with Staphylococcus aureus ATCC 25923 and Pseudomonas aeruginosa ATCC 15442 Biofilms
Source: Int J Mol Sci. 2020 Apr 25;21(9):3034. doi: 10.3390/ijms21093034 (PMC7246797; doi:10.3390/ijms21093034)

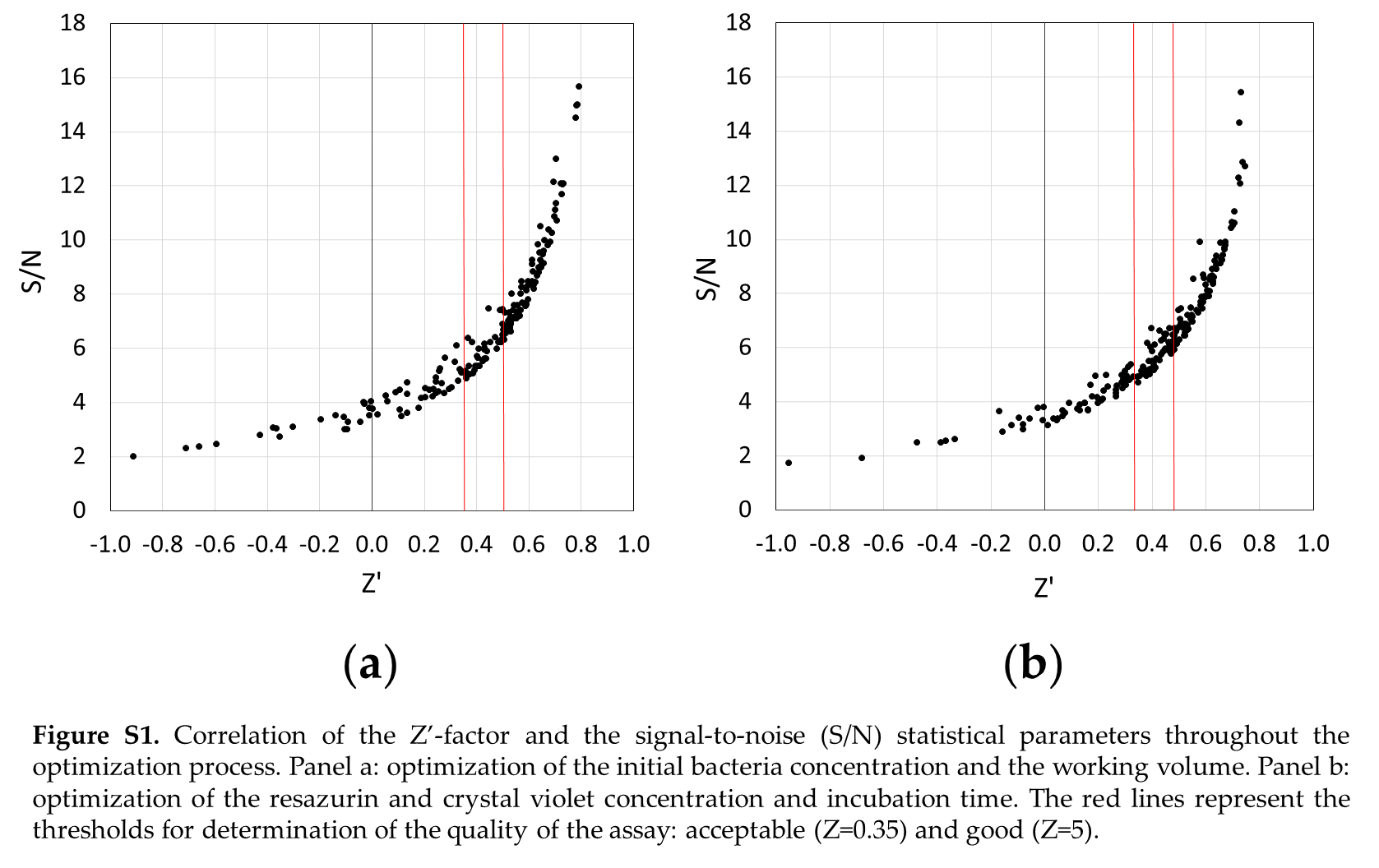

Supplement: Supplementary file 1 [file ijms-21-03034-s001.zip › ijms-782205-SI/Supplementary files_Gilbert-Girard et al/Supplementary Figure 1.jpg]

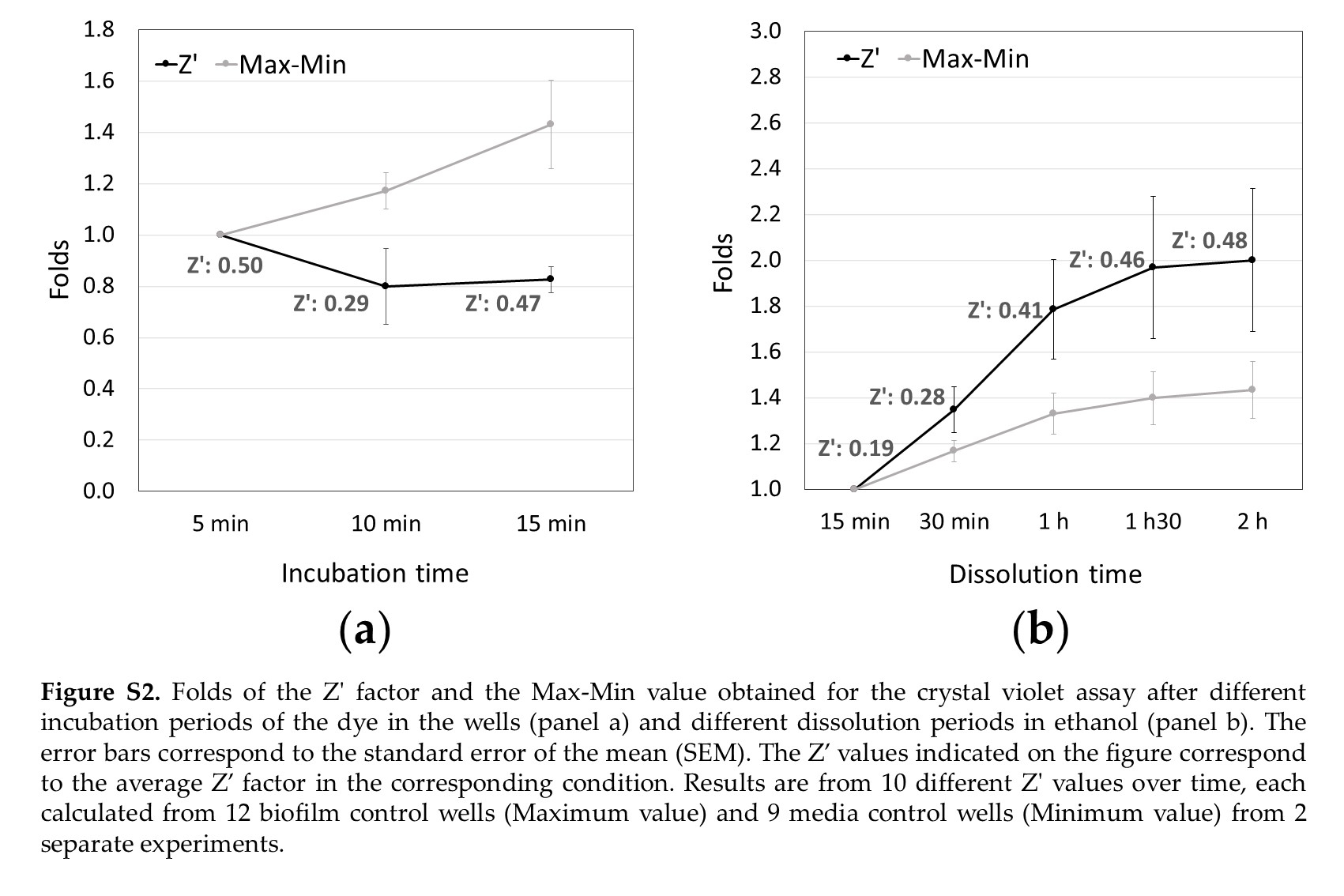

Supplement: Supplementary file 1 [file ijms-21-03034-s001.zip › ijms-782205-SI/Supplementary files_Gilbert-Girard et al/Supplementary Figure 2.jpg]
